# Parental bereavement – impact of death of neonates and children under 12 years on personhood of parents: a systematic scoping review

**DOI:** 10.1186/s12904-021-00831-1

**Published:** 2021-09-04

**Authors:** Prachi Simran Vig, Jia Yin Lim, Randal Wei Liang Lee, Huixin Huang, Xiu Hui Tan, Wei Qiang Lim, Marie Bernadette Xin Yi Lim, Alexia Sze Inn Lee, Min Chiam, Crystal Lim, Vijayendra Ranjan Baral, Lalit Kumar Radha Krishna

**Affiliations:** 1 Yong Loo Lin School of Medicine, National University of Singapore, 1E Kent Ridge Road NUHS Tower Block, Level 11, Singapore, 119228 Singapore; 2 Division of Palliative and Supportive Care, National Cancer Centre Singapore, 11 Hospital Crescent, Singapore, 169610 Singapore; 3 Division of Cancer Education, National Cancer Centre Singapore, 11 Hospital Crescent, Singapore, 169610 Singapore; 4 Medical Social Services, Singapore General Hospital, Outram Road, Block 3 Level 1, Singapore, 169608 Singapore; 5 Duke-NUS Medical School, 8 College Road, Singapore, 169857 Singapore; 6 Division of Neonatal and Developmental Medicine, Singapore General Hospital, Outram Road, Block 5 Level 4, Singapore, 169608 Singapore; 7 Palliative Care Institute Liverpool, Academic Palliative & End of Life Care Centre, University of Liverpool, Liverpool, UK; 8 Centre for Biomedical Ethics, National University of Singapore, Blk MD11, 10 Medical Drive, #02-03, Singapore, 117597 Singapore; 9 PalC, The Palliative Care Centre for Excellence in Research and Education, PalC c/o Dover Park Hospice, 10 Jalan Tan Tock Seng, Singapore, 308436 Singapore; 10 Cancer Research Centre, University of Liverpool, 200 London Rd, Liverpool, L3 9TA UK

**Keywords:** End of life, Palliative care, Death, Neonate, Infant, Paediatrics, Parents, Ring theory of personhood, Personhood, Bereavement

## Abstract

**Background:**

Losing a child tragically impacts the well-being and functioning of parents. With these effects extending beyond emotional, physical morbidity and compromising self-perceptions, appropriate, longitudinal, timely and personalised support is key to effective care of bereaved parents. However, in the absence of a comprehensive understanding of parental bereavement, effective support of bereaved parents remains suboptimal. To address this gap, we scrutinise prevailing data on the effects of a child’s death, aged 0–12 years, through the lens of the Ring Theory of Personhood (RToP).

**Methods:**

To study prevailing accounts of bereaved parents following the death of a child, we adopt Krishna’s Systematic Evidence Based Approach (SEBA) to structure our Systematic Scoping Review (SSR in SEBA).

**Results:**

Three thousand seventy-four abstracts were reviewed, 160 full text articles were evaluated, and 111 articles were included and analysed using thematic and content analysis. Four themes/categories were identified relating to the four rings of the RToP. Findings reveal that static concepts of protective and risk factors for grief are misplaced and that the support of healthcare professionals is key to assisting bereaved parents.

**Conclusion:**

In the absence of consistent support of bereaved parents, this study highlights the need for effective training of healthcare professionals, beginning with an appreciation that every aspect of an individual parent’s personhood is impacted by the loss of their child. Acknowledging grief as a complex, evolving and personalised process subjected to parental characteristics, settings, context and available support, this SSR in SEBA calls attention to effective nurturing of the relationship between parents and healthcare professionals, and suggests use of the RToP to assess and direct personalised, timely, specific support of parents in evolving conditions. We believe the findings of this review also call for further studies to support healthcare professionals as they journey with bereaved parents.

**Supplementary Information:**

The online version contains supplementary material available at 10.1186/s12904-021-00831-1.

## Background

The loss of a child has tragic implications upon a parent’s wellbeing [1, 2] and social function [3]. Evidence of protracted emotional distress [4], higher divorce rates [5], increased psychiatric [6] and medical admissions [7, 8], greater physical [9] and emotional [10] morbidity and higher mortality [11] also suggests impact upon the bereaved parent’s beliefs, values, principles [12], spiritual concepts [13], their existential, spiritual, individual, relational, medical and societal roles, needs and goals [8, 14–17], and their relationships with family members, close friends, and members of society [18]. Some authors have suggested that such deep and diverse change may be framed as a change in the bereaved parent’s sense of self [19–23]. Such a posit finds support from Bartel [24]’s account of grieving families, Mahat-Shamir [25]’s report on parental experiences following the loss of their child and Einarsdóttir [26]’s article on maternal grief.

It is to this sense of disruption of a parent’s concept of personhood or “what makes ‘you’ you” [27–35] that we turn our attention to in order to better understand the impact of such loss, and to better direct timely, personalised, appropriate, holistic and longitudinal support to bereaved parents. Thus, a review of current data on the effects of a child's death on a parent through the lens of the Ring Theory of Personhood (henceforth RToP) [27, 35] was carried out.

### Krishna’s Ring Theory of Personhood

The employ of the RToP to capture the impact of bereavement is not new [24–32]. The RToP has been used within other Palliative Medicine settings to study changes in thinking, values, beliefs, roles and relationships amongst terminally ill patients [24–32]. Here extrapolating its use to bereaved parents finds support from Kuek, Ngiam [32]’s study of the impact of caring for dying patients upon physicians in the intensive care.

**Fig. 1:**
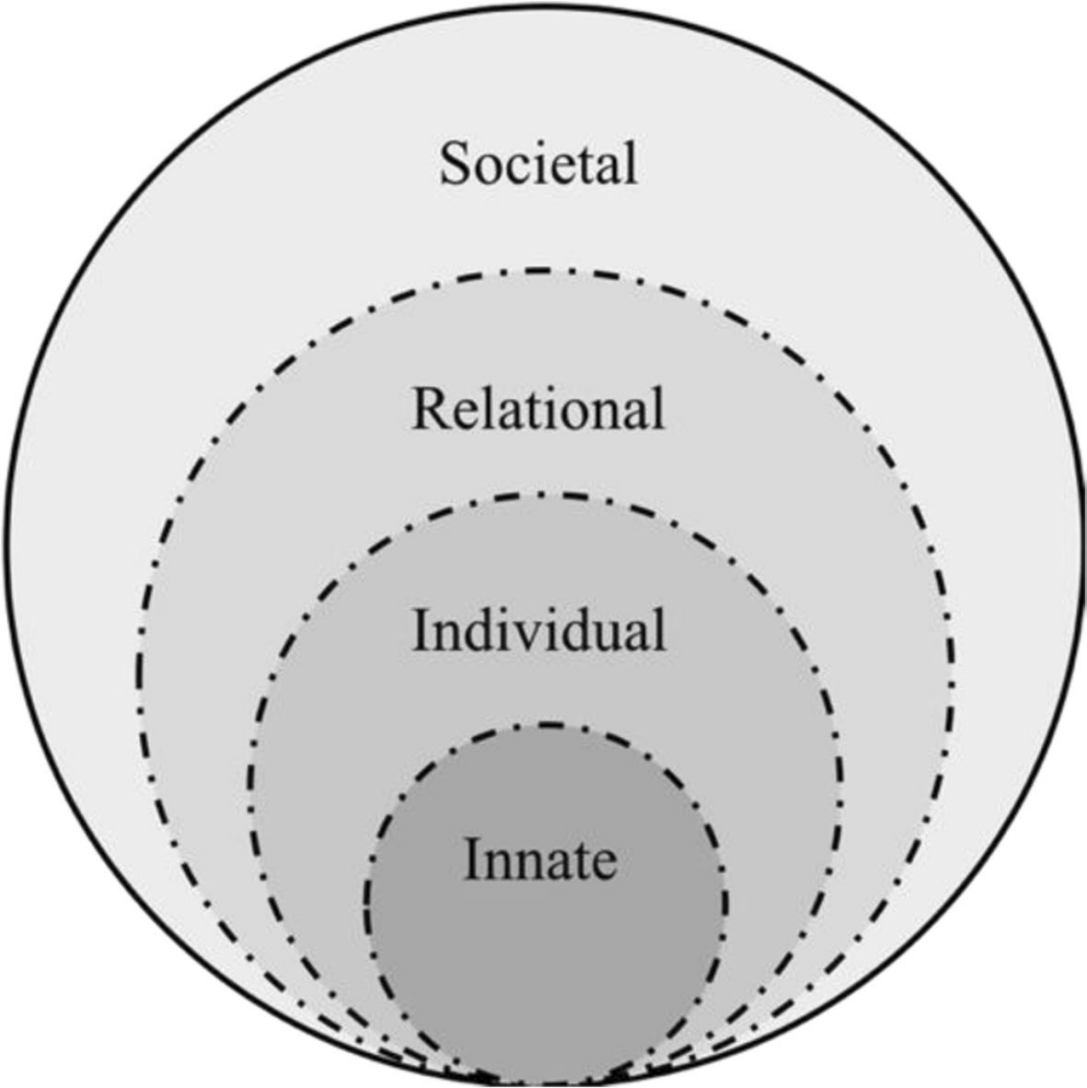
The Ring Theory of Personhood

Here, the RToP’s unique ability to capture change in the parent’s perspective of themselves and their relationships [36], roles in the family and in society [26] also leaves it best placed to capture liminality which Turner [37] defines as “entities ... neither here nor there; they are betwixt and between the positions assigned and arrayed by law, custom, convention, and ceremony” [25]. The insights provided will greatly enhance support of bereaved parents. Here, a better understanding of the RToP’s four domains depicted as four interconnected rings – the Innate, Individual, Relational and Societal rings is required (Fig. 1).

The Innate Ring is the innermost ring of the RToP and may be seen to derive itself from the genes that define oneas human and the individual’s religious beliefs, such as their ties with a Higher Power. The Innate Ring is also shaped by historical factors such as the person’s gender, race, culture, religion and heritage that they are born into. The combination of all these considerations confers the individual with respect and rights reserved for all human beings until their death. Here, the RToP's dynamic nature may be used to capture change in the bereaved parent’s [38] spiritual beliefs and existential coping.

The Individual Ring builds upon the Innate Ring and relates to the person’s conscious function, ability to think, feel, communicate, act and maintain his or her own personhood. The loss of conscious function eliminates the Individual Ring. The Individual Ring guides the motivations, inclinations, thoughts, traits and actions behind their individual identity. The RToP’s ability to detect change in the Individual Ring will help healthcare professionals better understand the bereaved parent’s thought process, emotions and coping mechanisms [39, 40].

The Relational Ring contains relationships that the individual determines to be important to them. These relationships may be with family members or friends. For bereaved parents, it is members of the Relational Ring that often provide comfort and support. Changes within the Relational Ring will highlight support and stressors upon the bereaved parent [41].

The Societal Ring, which is the outermost ring, contains relationships with colleagues, acquaintances, contacts and members of networks that the individual does not have significant personal ties with. The Societal Ring also houses cultural norms, professional standards, and societal obligations such as familial, professional, and societal expectations prescribed to the individual within their role in the community. The Societal Ring will also capture society’s support and consideration for the bereaved parent as well as the parent’s perception of their cultural and social roles and responsibilities [42, 43].

Perhaps more significantly, each ring contains specific values, beliefs, principles, and expectations that come together within the Individual Ring and influence preferences, motivations, decisions and biases, thoughts, and actions. This highlights the interrelatedness of the rings and the central role of the Individual Ring. Concurrently, changing conditions [44], evolving contextual [45], existential, personal, relational, and societal considerations also impact the individual’s thoughts and actions. This underlines the importance of the RToP’s ability to capture changes in thinking, coping mechanisms[39, 40], needs, motivations in the parent and explain their decisions [46] and actions [47] which will then guide their timely, personalised and targeted support [48].

## Methodology

A systematic scoping review (SSR) has been undertaken to study the scope and depth of current data on the complex multidimensional aspects of grief [49, 50] and ‘meaning-making’ [51–53] upon the personhood of bereaved parents. The SSR’s flexible approach facilitates identification of patterns, relationships, and disagreements within regnant quantitative and qualitative data drawn from a wide range of study formats and settings.

However, the reproducibility and transparency of current forms of SSRs are subject to concern due to a lack a consistent approach to structuring, reporting and analysis of the included data. To counter these issues, Krishna’s Systematic Evidence Based Approach (SEBA) guided SSR (henceforth SSR in SEBA) is adopted. Built on a constructivist perspective and a relativist lens, SSRs in SEBA are able to effectively contend with the notion of psychological constructivism [54] used to describe ‘meaning construction’ in grief [55] and provide a longitudinal, context dependent [56, 57], socioculturally and ideologically appropriate understanding [58, 59] of the grieving process and its sequalae [60, 61]. A holistic approach also helps address ethical concerns [62] surrounding research on bereaved parents.

In keeping with the SEBA methodology, a team of experts was engaged to oversee and advise the research team at all stages of the research process. This expert team included a medical librarian from the Yong Loo Lin School of Medicine (YLLSoM) at the National University of Singapore (NUS) and local educational experts and clinicians at the National Cancer Centre Singapore (NCCS), Palliative Care Institute Liverpool, YLLSoM and Duke-NUS Medical School. They served to enhance the accountability of the SSR in SEBA findings.

**Fig. 2:**
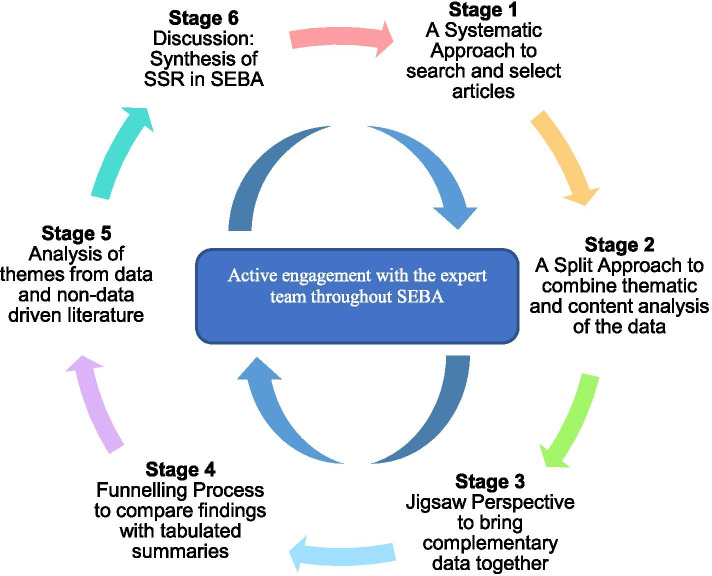
The SEBA Process

Conforming to the SEBA methodology, the research and expert teams adopted the principles of interpretivist analysis and immersed themselves in the data through repeated reading, analysis and reflexive discussions so as to piece the qualitative data together in a meaningful manner [63–66]. The SEBA process comprises of the following six stages: 1) Systematic Approach, 2) Split Approach, 3) Jigsaw Perspective, 4) Funnelling Process 5) Analysis of themes from data and non-data driven literature, and 6) Discussion: Synthesis of SSR in SEBA (Fig. 2). These are applied and elaborated upon below.

### STAGE 1 of SEBA: Systematic Approach

- i.Determining the title and background of the review
  In order to ensure a systematic, reproducible and transparent approach to the review, the expert and research team discussed and agreed upon the overall goals, study population, context and concept to be evaluated.
- ii.Identifying the research question
  The four members of the research team discussed the research question with a medical librarian from the expert team. Guided by the Population Concept, Context (PCC) elements of the inclusion criteria, the research question was determined to be: “How is the personhood of parents affected by the death of their child (aged 0 to 12 years)?” The secondary research questions were determined to be: “What are the key characteristics of the bereavement process for parents?” and “How do their relationships with others change following the death of their child?” To help focus attention upon the loss of a child we adopted the World Health Organisation’s classification of a child and a young child [67].
- iii.Inclusion criteria
  All grey literature, peer reviewed articles, narrative reviews, systematic, scoping and systematic scoping reviews published between 1^st^ January 2000 and 31^st^ December 2020 were included in the PICOs [68, 69] outlined in Table 1.
- iv.Searching
  Four members of the research team carried out independent searches of four bibliographic databases (Pubmed, EMbase, Psychinfo, CINAHL) and a grey literature database (Google Scholar). In keeping with Pham, Rajić [70]’s recommendations on ensuring a viable and sustainable research process, the research team confined the searches to articles published between 1^st^ January 2000 and 31^st^ December 2020. The searches were carried out between 18^th^ October 2020 and 17^th^ January 2021 using the following search terms: “personhood”, “selfhood” AND “end of life”, “palliative care” AND “death”, “sudden death”, “neonate” AND “infant”, “paediatrics’” and their combinations. The search strategies are presented in Additional file 1.
- v.Extracting and charting
  Using an abstract screening tool, the research team independently reviewed the titles and abstracts to identify a list of relevant articles believed to be of relevance that met the inclusion criteria that were set out in Table 1. Next, they individually evaluated full text articles within this filtered list in a second sieving process, resulting in a final list of included articles. These individual lists were discussed amongst the researchers at online meetings and Sandelowski and Barroso [71]’s ‘negotiated consensual validation’ was used to achieve consensus on the final list of articles to be included. Here, negotiated validity sees “*research team members articulate, defend, and persuade others of the “cogency” or “incisiveness” of their points of view or show their willingness to abandon views that are no longer tenable. The essence of negotiated validity is consensus*.” (p.229) This final list was then reviewed by the last author.

**Table 1:** PICOs inclusion and exclusion criteria

	**Inclusion criteria**	**Exclusion criteria**
Population	● Biological parents and/or legal guardians of deceased neonates and/or children up to the age of 12 years of life [67]	● Parents who are not related by the law or by blood ● Non-1st degree relatives ● Patients who are adolescents above the age of 12
Intervention	● Intervention programmes for parents of deceased neonates up to the age of 12 between 1st January 2000 and 31st December 2020	NA
Comparison Outcome	● Outcomes of intervention programmes ● Changes in parents’ personhood ● Programme evaluation results, from forms and questionnaires done by bereaved parents ● Gaps and improvements to current intervention programmes ● Views of bereaved parents on healthcare and intervention programmes	● Observations and recounts of parental behaviour by healthcare staff
Study design	● All study designs and article types were included (observational studies, randomised controlled trials, cohort studies, cross-sectional studies, longitudinal studies and case studies, ancestry approach/review) in the English language	● Non-English language articles

### STAGE 2 of SEBA: Split Approach

The research team was divided into two teams to concurrently analyse the included articles using Braun and Clarke [72]’s approach to thematic analysis and Hsieh and Shannon [73]’s approach to directed content analysis. Also known as the Split Approach, this method allows focus on key aspects of the “*entire experience of anticipating a death, the death itself and the subsequent adjust to living*” [12]. All 111 articles were read and reviewed by both research teams independently.

#### Thematic analysis

In Phase 1 of Braun and Clarke approach, an iterative step-by-step thematic analysis was carried out by a team of three researchers who who independently and actively read the included articles to identify meaning and patterns. In Phase 2, ‘codes’ were constructed from the ‘surface’ meaning and collated into a code book to code and analyse the rest of the articles using an iterative step-by-step process. As new codes emerged, these were associated with previous codes and concepts. In Phase 3, codes were organised into themes that *“represent some level of patterned response or meaning within the data set*” [74]. In Phase 4, each member of the research team refined their themes to ensure they were coherent and representative of the whole data set. After completing the first 4 phases, the team came together in Phase 5. In this phase, the team discussed the results of their independent analysis online and at reviewer meetings. ‘Negotiated consensual validation’ was used to determine the final list of themes.

#### Directed content analysis

Hsieh and Shannon’s approach to directed content analysis was employed to enhance the validity of the findings, add ‘consistency’ to the delineation of themes by drawing upon prevailing codes and categories, and address the relative failure of thematic analysis to address contradictory data [73].

The first stage saw three reviewers draw codes and categories from Krishna [35]’s article entitled “*Accounting for personhood in palliative sedation: the Ring Theory of Personhood*” which was chosen due to its holistic study of various aspects of personhood amongst terminally ill patients. Each code was defined in the code book and used in the second stage to independently extract and code relevant data from the included articles. In keeping with deductive category application, any relevant data not captured by these codes were assigned a new code. “Negotiated consensual validation*”* was used to achieve consensus on the codes, and this code book was then used to code the rest of the articles [75].

### STAGE 3 of SEBA: Jigsaw Perspective

Here, the Jigsaw Perspective saw the themes and categories viewed as pieces of a jigsaw puzzle where areas of overlap allowed for these pieces to be combined to create a bigger picture of the overlying data. The combined themes and categories are referred to as themes/categories. The Jigsaw Perspective employs Phases 4 to 6 of France, Wells [76]’s adaptation of Noblit and Hare [77]’s seven phases of meta-ethnography.

As per France, Wells adaptation, the themes and categories identified in the Split Approach were grouped according to their focus. Each theme and category within the group were contextualised by reviewing the articles from which they were drawn. Reciprocal translation determined if the new data provided by the respective themes and categories could be used interchangeably

### STAGE 4 of SEBA: Funnelling Process

The funnelling process sees the themes/categories identified in the Jigsaw Perspective compared with the tabulated summaries created, in keeping with recommendations set out by Wong, Greenhalgh [55]’s RAMESES publication standards: meta-narrative reviews and Popay, Roberts [78]’s “Guidance on the conduct of narrative synthesis in systematic reviews”. The tabulated summaries ensured that the themes/categories identified provided an accurate representation of existing data (see Additional file 2). They also included quality appraisals using the Medical Education Research Study Quality Instrument (MERSQI) [79] and the Consolidated Criteria for Reporting Qualitative Studies (COREQ) [80].

The Funnelling Process employed Phases 3 to 5 from France, Wells adaptation where the themes/categories identified in the Jigsaw Perspective were juxtaposed with key messages identified in the tabulated summaries. The funnelled themes/categories formed the basis for the discussion narrative’s ‘line of argument’ in Stage 6 of SEBA.

## Results

**Fig. 3:**
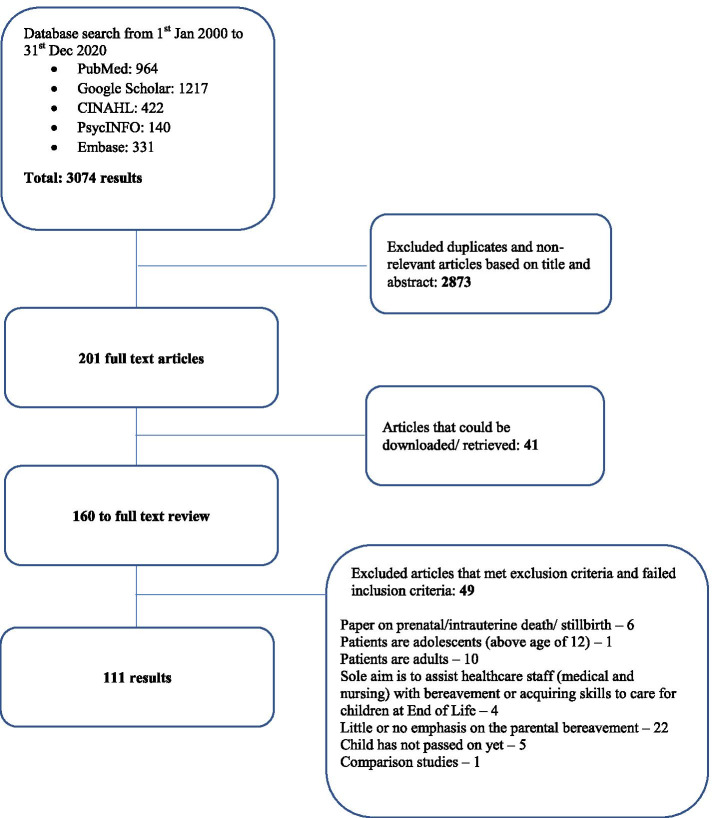
PRISMA flow chart

A total of 3074 abstracts were reviewed, 160 full text articles evaluated, and 111 articles included as outlined in Fig. 3 below. Of the included articles, 14 were quantitative studies, 52 were qualitative studies and 20 were mixed studies.

In the interest of space and ease of review, the themes and categories identified are summarised in Table 2.

**Table 2:** Themes and categories identified

Themes identified through Thematic Analysis	Categories identified through Directed Content Analysis
1) Honour memory oPreserving deceased child’s presence in family life o Living in a way that honours their deceased child o Remembering their child/keeping memory of child alive o Forgetting memory of child 2) Spirituality/ Religious mores/ practices 3) Personality 4) Attitude to Life and Death o Grief-alleviating factors o Grief worsening factors o Anticipatory Grief o Behavioural Manifestations o Coping Mechanism 5) Strengthening relationship ▪ between parents and children, other family members, ▪ and healthcare staff o Weakening relationship ▪ between parents and children, other family members, and healthcare staff o Familial duties o Changing membership (promotion/ severing of ties)/ new memberships 6) Societal relationships o Professional relationships o Interaction with healthcare professionals o Acquaintances o Societal expectations	1) Innate Ring 2) Individual Ring 3) Relational Ring 4) Societal Ring

The Funnelled themes/categories were as follows.

- 1) Innate Ring
  - ▪ Spirituality/Religion
- 2) Individual Ring
  - ▪ Attitude to Life and Death
    - ▪ Positive thinking
    - ▪ Setting and timing of child’s death
  - ▪ Coping Mechanism
    - ▪ Positive coping
    - ▪ Development of psychosocial morbidities
- 3) Relational Ring
- 4) Societal Ring

### Funnelled Themes/ Categories 1: The Innate Ring

#### Spirituality/ Religion

Whilst spiritual support is generally seen to help a parent’s coping and grieving process [81–85], attenuate shock, disbelief, and yearning [86], reduce parental grief (despair, detachment, and disorganisation), improve mental health (depression, post-traumatic stress), enhance personal growth [85], remain connected to their deceased child [28] and find meaning in their loss [84, 87–90], being aware of how the individual parent is addressing his or her grief is critical.

Falkenburg, van Dijk [91] found that many parents had their faith profoundly shaken [91–94]. Frei-Landau, Hasson-Ohayon [59] suggest that there are three manifestations of existential or divine struggles. They are: absence of divine struggle, an explicit divine struggle where the parent seeks explanations, and an implicit divine struggle where the parent does not discuss religiosity [61]. Understanding these states is critical to the provision of effective support, since there is no consistency as to how religious affiliations across cultures impact grief in parents. For instance, South American families reported that their spirituality [95] was compromised by evangelical family and/or friends [96] whilst self-reported nonreligious participants in Beijing found religious mores a source of support that enhanced spirituality [97]. Similarly, whilst Hedayat [98] suggested that in Muslim societies the death of a child a reinforced their religious faith [92], some bereaved parents believed that their child’s death was a punishment from a Higher Power [89, 99, 100] and turned their disappointment inward and or towards the Higher Power [99].

van der Geest, van den Heuvel-Eibrink [100] found that religious beliefs were less affected in some societies like the Netherlands where religion appears to play a less significant role in the society as compared to the United States where religion is seen as a significant source of support.

### Funnelled Themes/ Categories 2: The Individual Ring

Bereavement is influenced by personality traits [59], culture, demographics [101], attitudes, values, and beliefs as well as previous stressful encounters and one's physical and emotional health [102–104].

### Attitude to Life and Death

- i.Positive thinking towards death
  Some parents viewed their child’s death as relief from pain and suffering [92, 105], an inevitable part of life [106] or a ‘greater plan’ [107], thus reducing fears of their own mortality [108, 109]. These provided them with a source of courage [106, 109], a newfound appreciation for life [92] and even hope and solace [86, 93].
- ii.Setting and timing of child’s death

The setting and timing of a child’s death greatly impacts the parent’s bereavement. A death following a protracted illness predisposed them to complicated grief [110] and desensitisation [111]. On the other hand, being aware of the child’s prognosis helped prepare some parents [110, 112]. Direct, timely, and personalised communications [113], effective end of life care [111, 114–124], respect for advance care planning (ACP) [111] and parental involvement in care determinations also assuaged parental grief [84, 125, 126].

### Coping

- i.Positive Coping
  Parents who could share their feelings [127, 128], and received guidance [116, 117, 119, 127, 129, 130] and frequent updates on their child’s prognosis and health status [131, 132] in an honest [120], understandable [133], consistent [114, 117, 128], compassionate [90, 111, 115, 118, 119, 134] and culturally sensitive manner [87, 117, 128, 135, 136] coped better and suffered less grief [88, 90, 120, 127, 129, 131, 133, 136–142]. Parents found that being present at their child’s death [140, 143] ameliorated the grieving process [134, 140] as “*seeing and holding or being allowed to touch and hold helps the bereaved person adapt to the loss and say goodbye*” [98, 126, 144, 145].
  Also helpful were healthcare professionals who maintained contact with the parents after the child’s demise [81, 83, 88, 108, 114, 135, 138, 140, 146, 147]. These relationships created a social space that kept the child’s identity alive [139] and facilitated personalised home-based bereavement services [10, 87, 143, 146, 148, 149] both for the individual [82] and for the couple [82, 112, 149, 150].
  Positive coping facilitated personal growth [109], resilience [90, 106, 132], better self-understanding [89, 109], meaning making [87, 90] and motivation to invest in self-care [132]. Parents with positive coping mechanisms were also more appreciative of non-material values such as their health and family [90, 92, 95, 108]. Parents also recovered faster [87, 108, 148, 151] through personal and societal undertakings such as by giving back to society in their deceased child's name [106, 109, 134, 149, 152], donating their child’s organs [153], creating a trust fund or foundation [109, 142, 154], and/or organising support groups for other parents in similar situations [87, 90, 92, 109, 127, 138, 155]. On a personal level, parents coped better by commemorating their deceased child on their birthday or death anniversary [82, 89, 96, 138, 156], visiting their child’s grave, holding on to their child’s possessions or keeping photographs, footprints, locks of hair, quilts or toys [89, 95, 115, 116, 127, 156–158] and creating “memory boxes” [92, 115, 127] that serve to reaffirm the deceased child’s place within the family [140].
- ii.Development of psychosocial morbidities
  Psychosocial morbidities were precipitated by anticipatory grief [159], loss of parental role [113, 160], and a sudden death [161]. Exacerbation of parental grief [10, 146] was also noted when the child experienced poor symptom management [10, 108, 143, 146, 151, 152], prolonged illness [120], and a hospital death [162], particularly when the parent was separated from the child [131, 148], insufficiently prepared for the loss [92, 100, 113, 116, 131, 150, 163], and faced a lack of conducive environment to say goodbye [148, 164]. Psychosocial morbidities were exacerbated by poor transition of care [114, 127, 129, 140, 143, 148], poor communication [10, 82, 116, 117, 129, 131, 132, 135, 143, 148, 165–167], unrealistic prognostication [10, 113, 129, 132, 135, 138, 165, 166], role conflict between parents and healthcare providers during end of life decision making [167–169] and the healthcare providers’ lack of cultural sensitivity [170]. Anger, fear and guilt [96, 106, 117, 118, 134, 163, 171], anxiety, depression, post-traumatic stress symptoms [89, 158, 163, 168, 172, 173], insomnia [89], permanently damaged parental self-concept [168], role confusion [171], poor social function [10, 89, 99], functional impairment such as phobias or somatic problems [81, 92, 155, 163, 174], suicidal ideation and prolonged grief [10, 89] were also exacerbated by inadequate social [82, 88, 120, 148, 154, 161, 165, 175], spiritual [82, 88, 98, 176, 177] and bereavement support [82, 83, 89, 92, 108, 109, 127, 141, 143, 148, 150, 152, 154, 163, 178].
  Inadequate gender and personalised bereavement support also raised concerns [179]. Fathers were found to refrain from expressing their grief openly [95, 99], isolate themselves from family and friends, shun spiritual services [99], exhibit a greater need for respite from their child’s care [179] and succumb to delayed grief reactions [161]. Mothers were found to be more likely to become depressed [95], experience more physical ailments and social ill-health [174] and require longer recovery [116, 177].

### Funnelled Themes/ Categories 3: The Relational Ring

Scocco, Idotta [101] suggest that coping with bereavement is also influenced by the relationship shared with the child, the circumstances of the death and the consequences of the death. Bartel [24] noted that the death of the child brings about “*relational grieving*” for the individual, family unit and the larger community.

Whilst such loss can strengthen marital relationships [106, 155, 158] and provide distinct mutual spousal support [89, 90], spousal ties may be frayed by disagreements, stress and grief [39, 92, 112, 134]. Here, involvement of the parents’ other children in the grief-sharing process may help the family come closer together [92, 109] and give life meaning [106]. Support from friends and extended family may also help to strengthen these ties [88, 137, 140, 154, 155, 176].

Conversely, neglect of the other children jeopardised nuclear and extended familial ties and dynamics [82, 83, 95, 143, 148, 150, 155]. Ties between parents, friends and family may also be weakened when life-and-death decisions are not mutually supported [180] or misunderstood [90]. Relationships also suffer as a result of insensitive communications [85, 89, 90, 167] and when bereaved parents subconsciously isolate themselves from others [89].

### Funnelled Themes/ Categories 4: The Societal Ring

Honest, timely, personalised, empathetic, kind, culturally sensitive and respectful support enable healthcare professionals to provide more effective ‘external’ and longitudinal assistance to parents [115, 118, 120, 129, 130, 133, 137, 139, 146, 149, 150, 167, 181]. It also helps to affirm parents of their place in society [81, 82, 133, 136–139, 148, 163, 176]. Other bereaved parents may also serve as a further source of advice and guidance [89, 130, 182].

However, poor bereavement support may leave parents feeling ‘abandoned’ [82, 85, 114, 120, 127, 176, 183], resulting in the feeling of having suffered a ‘double loss’ [109] or ‘multiple losses’ [111, 184].

### STAGE 5 of SEBA: Analysis of themes from data and non-data driven literature

Acknowledging the potential impact of poor quality appraisal scores amongst the largely non-evidence based grey literature and opinions, perspectives, editorials, letters and non-primary data-based articles underlined the need to assess the impact of such data on the synthesis of the discussion portion of this SSR in SEBA. The research team found that the themes identified from separate thematic analysis of evidence-based and non-evidence-based data were similar, suggesting that the latter included in this review did not bias the analysis untowardly.

### Stage 6 of SEBA: Synthesis of SSR in SEBA

In keeping with SEBA, the discussion portion of this SSR in SEBA was guided by the Best Evidence Medical Education (BEME) Collaboration guide [185] and the STORIES (Structured apprOach to the Reporting In healthcare education of Evidence Synthesis) statement [186].

## Discussion

In answering its primary research question, this SSR in SEBA highlights three key findings in viewing *the impact of death of a child between 0 to 12 years on the personhood of a parent* through the lens of the RToP. To begin, this review highlights evidence that the loss of a child will impact every aspect of a parent’s life. These changes to their spiritual beliefs, psychoemotional state , relationships, roles and expectations are captured within the Innate, Individual, Relational and Societal domains of the RToP. Secondly, perhaps more significantly, this SSR in SEBA highlights the dynamic nature of the bereavement process and the influence of a variety of factors within each of the four rings of the RToP. These findings undergird the notion of a personalised grief experience that requires an individualised, holistic, and longitudinal support mechanism. Overall, this data underscores the significant role that healthcare professionals play in supporting bereaved parents and in assessing and engaging with different elements of the parent’s life to better support them [187].

However, this review also highlights a number of consistent factors that attenuate and exacerbate the acute effects of loss (Table 3) that may help to guide healthcare professionals in their assessment and support of bereaved parents. Reiterating the importance of forming  personalised relationships with them, this data underlines the critical need to support healthcare professionals in meeting their various roles and responsibilities [188–192].

**Table 3:** Protective and risk factors

	Protective factors	Risk factors
**Innate Ring**	Presence of spiritual support, guidance and counsel [81–85]	Perceptions of guilt, anger, desperation and Divine punishment [89, 99, 100]
	Strong spiritual beliefs [151]	
	Belief in reunion with child in the afterlife [86, 93]	
**Individual Ring**	Viewing death as positive outcome Relief from suffering [92, 105] Greater purpose [107] Reduced fear of death [106] Appreciation of own mortality [108, 109]	Anger, fear and guilt to changes in the child’s condition [96, 106, 117, 118, 134, 163, 171]
	Well symptomatically cared for [114–120]	Protracted dying process [111, 120] Poor symptom management [10, 108, 143, 146, 151, 152]
	Frequent personalised and timely updates on child’s prognosis and condition [131, 132]	Being unaware of prognosis [112] Ineffectual preparation by healthcare professionals [92, 100, 113, 116, 131, 150, 163] Unrealistic prognostication [10, 113, 129, 132, 135, 138, 165, 166],
	Personalised communication [113]	poor communication [10, 82, 116, 117, 129, 131, 132, 135, 143, 148, 165–167]
	Being present at the death [98, 126, 140, 143–145]	Not involved in end-of-life decision [84] Separated from the child [131, 148] Lack of a conducive environment to say goodbye [148, 164]
	Respect for advanced care plan [111] Effective end of life care [121–124]	Inadequate social [82, 88, 120, 148, 154, 161, 165, 175] Inadequate spiritual [82, 88, 98, 176, 177] Inadequate bereavement support [82, 83, 89, 92, 108, 109, 127, 141, 143, 148, 150, 152, 154, 163, 178]
	Positive means of coping including: Remembering the child [87, 89, 90, 106, 109, 132] (memory boxes, commemorating anniversaries) [82, 89, 92, 95, 96, 115, 116, 127, 138, 156–158] Greater self-care [152, 155] Giving back to society [106, 109, 134, 149, 152] Donating organs [87, 90, 92, 109, 127, 138, 155] Re-dedicating their lives [107, 109, 134]	Anxiety, depression, post-traumatic stress symptoms [89, 158, 163, 168, 172, 173] Insomnia [89] Permanently damaged parental self-concept [168] Role confusion [171] Poor social function [10, 89, 99] Functional impairment such as phobias, or somatic problems [81, 92, 155, 163, 174] Suicidal ideation and prolonged grief [10, 89]
**Relational Ring**	Spousal support [89, 90]	Spousal disagreements, stress, grief [39, 92, 112, 134]
	Support from family and friends [88, 137, 140, 154, 155, 176]	No family/friends to support [83] Insensitivity from family/friends [85, 90, 167]
	Support from remaining children [92, 109]	Previous neglect of other children [82, 83, 95, 143, 148, 150, 155]
**Societal Ring**	Continued support from healthcare professionals who knew family and the child [81, 83, 88, 108, 114, 135, 138, 140, 146, 147]	Feeling ‘abandoned’ by the hospital staff [82, 85, 114, 120, 127, 176, 183] Reporting feeling of having suffered a ‘double loss’[109] or ‘multiple losses’ [111] following poor bereavement support [184]
	Trusting relationship with healthcare professionals [114–119, 147]	
	Able to share feelings [127, 128] Receive guidance [116, 117, 119, 127, 129, 130] Receive culturally appropriate care [87, 117, 128, 135, 136] Receive compassionate care [90, 111, 115, 118, 119, 134]	Lack of professional support [84, 125] Poor transition of care [114, 127, 129, 140, 143, 148] Role conflict [167–169] Loss of parental role [113, 160] Lack of cultural sensitivity [170]

### Relationships with healthcare professionals

Whilst this SSR in SEBA brings to the fore several considerations, perhaps the most significant and thus the focus of our discussion is the role of the relationship between bereaved parents and the healthcare professional journeying with them. Built upon trust, individualised relationships allow for the provision of appropriate, specific, timely, accessible, holistic, and longitudinal support [114–119, 147, 188, 193]. This is cultivated by attending to the parents' needs, preparing them for anticipated loss, providing them with a ‘locus of control’ [17] and opportunities to say goodbye and grieve in a manner that best reflects their particular beliefs and contextual considerations. Journeying [191] with the parents through their child’s terminal stages of life is important, but equally critical is sharing in their loss and providing support in their bereavement. Jensen, Weng [194] found that such support was not always available. Here, this SSR in SEBA not only highlights the need for such consistent longitudinal support for families, but underscores the importance of sustained training, debriefs and holistic guidance for the healthcare professionals who journey with them.

### Change

To meet their longitudinal roles and responsibilities, healthcare professionals should be equipped with longitudinal support and training. This is underlined by the knowledge that factors previously deemed supportive to coping and meaning-making may turn into risk factors and vice versa. With Knapp and Contro [95], Jonas, Scanlon [96], van der Geest, van den Heuvel-Eibrink [151] and Cai, Guo [97] highlighting the interchangeability of factors listed in either column in Table 3, data here suggests that the RToP’s ability to capture such change ought to be used in the training of healthcare professionals so that they are better able to respond appropriately to each individual parent’s bereavement needs [27–30, 33, 34, 195]. Addressing change in the rings of the RToP foregrounds the import of timely [57] and context sensitive [7] assessments of parental coping [25, 89, 196, 197] and careful involvement of various members of the bereaved parents’ friends, relatives and community.

### Training

With present accounts suggesting grief support training to be inadequate [198] and thus limiting one's ability to identify and provide bereaved parents with individualised [191] and responsive support [25, 197], this SSR in SEBA underscores the importance of healthcare professionals being well-trained to discern when and how to support them and which family members, friends, and communities may help to supplement their support system. Important, too, is training in developing open, mindful, personalised, empathetic, kind, and culturally sensitive communications skills. Particularly on how and when parents are consulted on care determinations and offered honest, consistent, understandable, and compassionate guidance, clinical updates and prognostications. Such skills should be complemented with training in the appreciation of the parents’ religious beliefs [84, 86, 88–90], individual meaning-making proceses [86, 93, 106, 108, 109], and support provided or desired from their spouse, close friends, families [106, 155, 158] andcommunity [89, 130, 140, 143].

### Operationalising the RToP

To aid these endeavours, we believe that it is possible to adapt the RToP’s four domains into a framework to assess each parent’s state and needs (Table 4). Critically, the tool will allow transparency and accountability in bereavement risk assessments, convey critical information within the multi-disciplinary healthcare team [165, 191, 193, 199], and provide a robust structure for documentations and follow-up. Additionally, it will also help determine the intensity of support provided to prevent parents from feeling ‘abandoned’ [191, 200] by healthcare providers.

**Table 4:** Using RToP’s four domains to assess parent’s state and needs

Innate ring: Spiritual needs and importance placed on it Sources of spiritual support	Individual Ring: Self-support Couple’s therapy Family support Other available support mechanisms Stressors and changing situations in their life Provide avenues to seek help Determine the role that family and friends have in supporting the particular parent and their own coping mechanisms
Relational ring: The support from those near and dear to parents The importance placed on this	Societal Ring: Determine support within the parent’s work environment and the larger social circle determine support of the remaining children in their own school environment Engagement with the parent’s general practitioner, district nurses and counselling teams

## Limitations

This SSR in SEBA was limited by use of the RToP which remains unproven in this context despite it being utilised in other populations in palliative care. There is also an array of methodological weaknesses amongst the included articles including failure to detail sample populations, information on care settings, place of death, duration of illness, available support, research methodologies, validity of findings as well as a lack of longitudinal data. There could also be differences in individual approaches during data analysis, though this was minimised as much as possible through corroboration with the team at each stage of the analysis. This raises questions as to the veracity of conclusions drawn and applicability of recommendations made beyond North America and Europe as most of the included articles were from these English-speaking Western countries.

## Conclusion

This SSR in SEBA has important implications for neonate, paediatric and bereavement units highlighting the longitudinal and personalised expectations upon healthcare professionals in this field. It also emphasises the need for training and support of these healthcare professionals, forwarding the idea of the RToP as a training tool and as an assessment tool to evaluate change in the individual parent’s coping abilities. We envision that this tool will help to guide the provision of timely, personalised, appropriate, holistic and longitudinal support for both the healthcare professional and the bereaved parent. As we look forward to engaging in future discussions in this critical area of study, we believe it is vital for further research to be conducted to better elucidate ways in which healthcare professionals may be more comprehensively trained and supported as they journey with these parents. In addition, it is crucial to further determine the viability of the RToP as an education and assessment tool.

## Supplementary Information

**Additional file 1.** Search Strategy.

**Additional file 2.** Tabulated Summaries.

## Data Availability

All data generated or analysed during this review are included in this published article and its supplementary files.

## Abbreviations

SSR: Systematic Scoping Review
NUS: National University of Singapore
YLLSoM: Yong Loo Lin School of Medicine
NCCS: National Cancer Centre Singapore
PCC: Population, Concept and Context
PICOS: Population, Intervention, Comparison, Outcomes, Study Design
RToP: Ring Theory of Personhood
SEBA: Systematic Evidence Based Approach

## References

[1] McBride BA, Schoppe SJ, Rane TR (2002). Child characteristics, parenting stress, and parental involvement: fathers versus mothers. J Marriage Fam.

[2] Song J, Floyd FJ, Seltzer MM, Greenberg JS, Hong J. Long-term effects of child death on parents' health-related quality of life: a dyadic analysis. Fam Relat. 2010;59(3):269–82. 10.1111/j.1741-3729.2010.00601.x PMC2910450 20676393

[3] Rogers CH, Floyd FJ, Seltzer MM, Greenberg J, Hong J. Long-term effects of the death of a child on parents' adjustment in midlife. J Fam Psychol. 2008;22(2):203–11. 10.1037/0893-3200.22.2.203 PMC2841012 18410207

[4] Dyregrov K, Nordanger D, Dyregrov A (2003). Predictors of psychosocial distress after suicide, SIDS and accidents. Death Stud.

[5] Murphy SA, Johnson LC, Wu L, Fan JJ, Lohan J. Bereaved parents' outcomes 4 to 60 months after their children's deaths by accident, suicide, or homicide: a comparative study demonstrating differences. Death Stud. 2003;27(1):39–61. 10.1080/07481180302871 12508827

[6] Li J, Laursen TM, Precht DH, Olsen J, Mortensen PB (2005). Hospitalization for mental illness among parents after the death of a child. N Engl J Med.

[7] Demi AS, Miles MS (1988). Suicide bereaved parents: emotional distress and physical health problems. Death Stud.

[8] Znoj HJ, Keller D (2002). Mourning parents: considering safeguards and their relation to health. Death Stud.

[9] Lannen PK, Wolfe J, Prigerson HG, Onelov E, Kreicbergs UC (2008). Unresolved grief in a national sample of bereaved parents: impaired mental and physical health 4 to 9 years later. J Clin Oncol.

[10] Rosenberg AR, Baker KS, Syrjala K, Wolfe J (2012). Systematic review of psychosocial morbidities among bereaved parents of children with cancer. Pediatr Blood Cancer.

[11] Li J, Precht DH, Mortensen PB, Olsen J (2003). Mortality in parents after death of a child in Denmark: a nationwide follow-up study. Lancet.

[12] Meert KL, Briller SH, Myers Schim S, Thurston C, Kabel A (2009). Examining the needs of bereaved parents in the pediatric intensive care unit: a qualitative study. Death Stud.

[13] Alvarenga WdA, de Montigny F, Zeghiche S, Polita NB, Verdon C, Nascimento LC. Understanding the spirituality of parents following stillbirth: A qualitative meta-synthesis. Death Stud. 2021;45(6):420–36. 10.1080/07481187.2019.1648336 31403372

[14] Bergstraesser E, Inglin S, Hornung R, Landolt MA (2015). Dyadic coping of parents after the death of a child. Death Stud.

[15] Cantwell-Bartl A (2018). Grief and coping of parents whose child has a constant life-threatening disability, hypoplastic left heart syndrome with reference to the dual-process model. Death Stud.

[16] Ellis JB, Stump JE. Parents' perceptions of their children's death concept. Death Stud. 2000;24(1):65–70. 10.1080/074811800200702 10915448

[17] Rubinstein G (2004). Locus of control and helplessness: gender differences among bereaved parents. Death Stud.

[18] Peach MR, Klass D (1987). Special issues in the grief of parents of murdered children. Death Stud.

[19] Gilbert KR. “We've had the same loss, why don't we have the same grief?” loss and differential grief in families. Death Stud. 1996;20(3):269–83.

[20] Neimeyer RA, Prigerson HG, Davies B (2002). Mourning and Meaning. Am Behav Sci.

[21] Shapiro ER (2007). Whose Recovery, of What? Relationships and Environments Promoting Grief and Growth. Death Stud.

[22] Lichtenthal WG, Currier JM, Neimeyer RA, Keesee NJ. Sense and significance: a mixed methods examination of meaning making after the loss of one's child. J Clin Psychol. 2010;66(7):791–812. 10.1002/jclp.20700 PMC3745996 20527057

[23] Van Humbeeck L, Dillen L, Piers R, Grypdonck M, Van Den Noortgate N (2016). The suffering in silence of older parents whose child died of cancer: A qualitative study. Death Stud.

[24] Bartel BT (2020). Families grieving together: Integrating the loss of a child through ongoing relational connections. Death Stud.

[25] Mahat-Shamir M. Neither here nor there and a little bit of both: The ambiguous loss experience of parents who lost a baby to sudden infant death syndrome. Death Studies. 2020:1–10. Advance online publication. 10.1080/07481187.2020.1802629 32757882

[26] Einarsdóttir J (2021). Maternal grief in cross-cultural context: Selective neglect, replaceable infants and lifesaving names. Death Stud.

[27] Radha Krishna LK, Alsuwaigh R (2015). Understanding the fluid nature of personhood - the ring theory of personhood. Bioethics.

[28] Krishna LKR, Yong CYL, Koh SMC (2014). The role of palliative rehabilitation in the preservation of personhood at the end of life. BMJ Case Rep.

[29] Krishna LKR (2013). Personhood within the context of sedation at the end of life in Singapore. BMJ Case Rep.

[30] Krishna LK, Alsuwaigh R, Miti PT, Wei SS, Ling KH, Manoharan D (2014). The influence of the family in conceptions of personhood in the palliative care setting in Singapore and its influence upon decision making. Am J Hosp Palliat Care.

[31] Ho CY, Kow CS, Chia CHJ, Low JY, Lai YHM, Lauw S-K (2020). The impact of death and dying on the personhood of medical students: a systematic scoping review. BMC Med Educ.

[32] Kuek JTY, Ngiam LXL, Kamal NHA, Chia JL, Chan NPX, Abdurrahman ABHM (2020). The impact of caring for dying patients in intensive care units on a physician’s personhood: a systematic scoping review. Philos Ethics Humanit Med.

[33] Alsuwaigh R, Krishna LKR (2014). The Compensatory Nature of Personhood. Asian Bioethics Rev.

[34] Krishna LKR, Kwek SY (2015). The changing face of personhood at the end of life: the ring theory of personhood. Palliat Support Care.

[35] Krishna LKR (2014). Accounting for personhood in palliative sedation: the Ring Theory of Personhood. Med Humanit.

[36] Neimeyer RA, Burke LA (2015). Loss, grief, and spiritual struggle: The quest for meaning in bereavement. Religion Brain Behavior.

[37] Turner VW (1969). The ritual process: structure and anti-structure.

[38] Brotherson SE, Soderquist J. Coping with a child's death. J Fam Psychother. 2002;13(1–2):53–86.

[39] Youngblut JM, Brooten D, Glaze J, Promise T, Yoo C (2017). Parent grief 1–13 months after death in neonatal and pediatric intensive care units. J Loss Trauma.

[40] Dyregrov A, Gjestad R (2011). Sexuality following the loss of a child. Death Stud.

[41] Schwab R. Effects of a child's death on the marital relationship: A preliminary study. Death Stud. 1992;16(2):141–54.

[42] Wikman A, Hovén E, Cernvall M, Ljungman G, Ljungman L, von Essen L (2016). Parents of children diagnosed with cancer: work situation and sick leave, a five-year post end-of-treatment or a child’s death follow-up study. Acta Oncol.

[43] Boyden JY, Kavanaugh K, Issel LM, Eldeirawi K, Meert KL (2014). Experiences of African American Parents Following Perinatal or Pediatric Death: A Literature Review. Death Stud.

[44] Barr P, Cacciatore J (2008). Personal Fear of Death and Grief in Bereaved Mothers. Death Stud.

[45] Wei Y, Jiang Q, Gietel-Basten S (2016). The well-being of bereaved parents in an only-child society. Death Stud.

[46] Votta E, Franche R-L, Sim D, Mitchell B, Frewen T, Maan C. Impact of parental involvement in life-support decisions: a qualitative analysis of parents' adjustment following their critically ill child's death. Child Health Care. 2001;30(1):17–25.

[47] Rosenblatt PC (2000). Protective parenting after the death of a child. J Personal Interpersonal Loss.

[48] Braun MJ, Berg DH (1994). Meaning reconstruction in the experience of parental bereavement. Death Stud.

[49] Gamino LA, Hogan NS, Sewell KW (2002). Feeling the absence: A content analysis from the Scott and White grief study. Death Stud.

[50] Gamino LA, Sewell KW. Meaning constructs as predictors of bereavement adjustment: a report from the Scott & White grief study. Death Stud. 2004;28(5):397–421. 10.1080/07481180490437536 15152644

[51] Greenhalgh T, Wong G. Training materials for meta-narrative reviews. UK: Global Health Innovation and Policy Unit Centre for Primary Care and Public Health Blizard Institute, Queen Mary University of London. 2013. https://www.ncbi.nlm.nih.gov/books/NBK260010/.

[52] Osama T, Brindley D, Majeed A, Murray KA, Shah H, Toumazos M (2018). Teaching the relationship between health and climate change: a systematic scoping review protocol. BMJ Open.

[53] Gerrish NJ, Neimeyer RA, Bailey S (2014). Exploring maternal grief: a mixed-methods investigation of mothers’ responses to the death of a child from cancer. J Constr Psychol.

[54] Neimeyer RA (2019). Meaning reconstruction in bereavement: development of a research program. Death Stud.

[55] Wong G, Greenhalgh T, Westhorp G, Buckingham J, Pawson R (2013). RAMESES publication standards: meta-narrative reviews. BMC Med.

[56] Lichtenthal WG, Neimeyer RA, Currier JM, Roberts K, Jordan N (2013). Cause of death and the quest for meaning after the loss of a child. Death Stud.

[57] Meij LW-d, Stroebe M, Stroebe W, Schut H, Bout JVD, Van Der Heijden PGM, et al. The impact of circumstances surrounding the death of a child on parents' grief. Death Stud. 2008;32(3):237–52. 10.1080/07481180701881263 18705169

[58] Neimeyer RA (2016). Meaning reconstruction in the wake of loss: evolution of a research program. Behav Chang.

[59] Frei-Landau R, Hasson-Ohayon I, Tuval-Mashiach R. The experience of divine struggle following child loss: the case of Israeli bereaved Modern-Orthodox parents. Death Stud. 2020:1–15. Advance online publication. 10.1080/07481187.2020.1850547 33259263

[60] Dijkstra IC, Stroebe MS (1998). The impact of a child’s death on parents: a myth (not yet) disproved?. J Fam Stud.

[61] Wikman A, Mattsson E, von Essen L, Hovén E (2018). Prevalence and predictors of symptoms of anxiety and depression, and comorbid symptoms of distress in parents of childhood cancer survivors and bereaved parents five years after end of treatment or a child’s death. Acta Oncol.

[62] Donovan LA, Wakefield CE, Russell V, Hetherington K, Cohn RJ (2019). Brief report: Bereaved parents informing research design: the place of a pilot study. Death Stud.

[63] Pring R (2000). The ‘false dualism’of educational research. J Philos Educ.

[64] Crotty M. The foundations of social research: meaning and perspective in the research process. UK: SAGE Publications Ltd; 1998.

[65] Ford K. Taking a narrative turn: possibilities, challenges and potential outcomes. OnCUE J. 2012;6(1):23–6.

[66] Schick-Makaroff K, MacDonald M, Plummer M, Burgess J, Neander W (2016). What synthesis methodology should i use? a review and analysis of approaches to research synthesis. AIMS Publ Health.

[67] Kwok PC, Chan HK (2014). Delivery of inhalation drugs to children for asthma and other respiratory diseases. Adv Drug Deliv Rev.

[68] Peters M, Godfrey C, McInerney P, Soares C, Khalil H, Parker D. The Joanna Briggs Institute reviewers' manual 2015: methodology for JBI scoping reviews2015 April 29, 2019. Available from: http://joannabriggs.org/assets/docs/sumari/Reviewers-Manual_Methodology-for-JBI-Scoping-Reviews_2015_v1.pdf.

[69] Peters MD, Godfrey CM, Khalil H, McInerney P, Parker D, Soares CB (2015). Guidance for conducting systematic scoping reviews. Int J Evid Based Healthc.

[70] Pham MT, Rajić A, Greig JD, Sargeant JM, Papadopoulos A, McEwen SA (2014). A scoping review of scoping reviews: advancing the approach and enhancing the consistency. Res Synth Methods.

[71] Sandelowski M, Barroso J. Handbook for synthesizing qualitative research. US: Springer Publishing Company; 2006.

[72] Braun V, Clarke V (2006). Using thematic analysis in psychology. Qual Res Psychol.

[73] Hsieh H-F, Shannon SE (2005). Three approaches to qualitative content analysis. Qual Health Res.

[74] Cassol H, Pétré B, Degrange S, Martial C, Charland-Verville V, Lallier F (2018). Qualitative thematic analysis of the phenomenology of near-death experiences. PLoS ONE.

[75] Sambunjak D, Straus SE, Marusic A (2010). A systematic review of qualitative research on the meaning and characteristics of mentoring in academic medicine. J Gen Intern Med.

[76] France EF, Wells M, Lang H, Williams B (2016). Why, when and how to update a meta-ethnography qualitative synthesis. Syst Rev.

[77] Noblit GW, Hare RD (1988). Meta-ethnography: synthesizing qualitative studies.

[78] Popay J, Roberts H, Sowden A, Petticrew M, Arai L, Rodgers M, et al. Guidance on the conduct of narrative synthesis in systematic reviews. J Epidemiol Community Health. 2005;59(Suppl 1):A7.

[79] Reed DA, Beckman TJ, Wright SM, Levine RB, Kern DE, Cook DA (2008). Predictive validity evidence for medical education research study quality instrument scores: quality of submissions to JGIM’s Medical Education Special Issue. J Gen Inter Med.

[80] Tong A, Sainsbury P, Craig J (2007). Consolidated criteria for reporting qualitative research (COREQ): a 32-item checklist for interviews and focus groups. Int J Qual Health Care.

[81] Zimmermann K, Bergstraesser E, Engberg S, Ramelet AS, Marfurt-Russenberger K, Von der Weid N, et al. When parents face the death of their child: a nationwide cross-sectional survey of parental perspectives on their child's end-of life care. BMC Palliat Care. 2016;15:30. 10.1186/s12904-016-0098-3 PMC4784404 26956995

[82] deCinque N, Monterosso L, Dadd G, Sidhu R, Macpherson R, Aoun S (2006). Bereavement support for families following the death of a child from cancer: experience of bereaved parents. J Psychosoc Oncol.

[83] Gear R. Bereaved Parents' Perspectives on Informal Social Support: “What Worked for You?” J Loss Trauma. 2014;19(2):173–88.

[84] Caicedo C, Brooten D, Youngblut JM, Dankanich J. Parents' wishes for what they had or had not done and their coping after their infant's or child's neonatal intensive care unit/pediatric intensive care unit/emergency department death. J Hospice Palliat Nurs. 2019;21(4):333–43. 10.1097/NJH.0000000000000559 PMC6610693 30933014

[85] Abdel Razeq NM, Al-Gamal E. Maternal bereavement: mothers' lived experience of losing a newborn infant in Jordan. J Hosp Palliat Nurs. 2018;20(2):137–45. 10.1097/NJH.0000000000000417 30063567

[86] Rosenbaum JL, Smith JR, Zollfrank R (2011). Neonatal end-of-life spiritual support care. J Perinat Neonat Nurs.

[87] Nuss SL (2014). Redefining parenthood: surviving the death of a child. Cancer Nurs.

[88] deJong-Berg MA, Kane L (2006). Bereavement care for families part 2: Evaluation of a paediatric follow-up programme. Int J Palliat Nurs.

[89] Currie ER, Christian BJ, Hinds PS, Perna SJ, Robinson C, Day S (2019). Life after loss: parent bereavement and coping experiences after infant death in the neonatal intensive care unit. Death Stud.

[90] Dutta O, Tan-Ho G, Choo PY, Low XC, Chong PH, Ng C (2020). Trauma to transformation: the lived experience of bereaved parents of children with chronic life-threatening illnesses in Singapore. BMC Palliat Care.

[91] Falkenburg JL, van Dijk M, Tibboel D, Ganzevoort RR (2020). The fragile spirituality of parents whose children died in the pediatric intensive care unit. J Health Care Chaplain.

[92] Laakso H, Paunonen-Ilmonen M. Mothers' grief following the death of a child. J Adv Nurs. 2001;36(1):69–77. 10.1046/j.1365-2648.2001.01944.x 11555051

[93] Armentrout D. Living with grief following removal of infant life support: parents' perspectives. Crit Care Nurs Clin North Am. 2009;21(2):253–65. 10.1016/j.ccell.2009.01.003 19460667

[94] Sturrock C, Louw J (2013). Meaning-Making After Neonatal Death: Narratives of Xhosa-Speaking Women in South Africa. Death Stud.

[95] Knapp CA, Contro N (2009). Family support services in pediatric palliative care. Am J Hosp Palliat Care.

[96] Jonas D, Scanlon C, Rusch R, Ito J, Joselow M. Bereavement after a child's death. Child Adolesc Psychiatr Clin N Am. 2018;27(4):579–90. 10.1016/j.chc.2018.05.010 30219219

[97] Cai S, Guo Q, Luo Y, Zhou Y, Abbas A, Zhou X (2020). Spiritual needs and communicating about death in nonreligious theistic families in pediatric palliative care: a qualitative study. Palliat Med.

[98] Hedayat K (2006). When the spirit leaves: childhood death, grieving, and bereavement in Islam. J Palliat Med.

[99] Hawthorne DM, Youngblut JM, Brooten D. Parent spirituality, grief, and mental health at 1 and 3 months after their infant's/child's death in an intensive care unit. J Pediatr Nurs. 2016;31(1):73–80. 10.1016/j.pedn.2015.07.008 PMC4975148 26320884

[100] van der Geest IM, van den Heuvel-Eibrink MM, Falkenburg N, Michiels EM, van Vliet L, Pieters R, et al. Parents' faith and hope during the pediatric palliative phase and the association with long-term parental adjustment. J Palliat Med. 2015;18(5):402–7. 10.1089/jpm.2014.0287 25679453

[101] Scocco P, Idotta C, Mareschi T, Preti A. Do interpersonal events buffer or worsen depressive and grief related symptoms in people bereaved through suicide? Death Stud. 2020:1–10. Advance online publication. 10.1080/07481187.2020.1855608 33287686

[102] Stroebe MS, Folkman S, Hansson RO, Schut H (2006). The prediction of bereavement outcome: development of an integrative risk factor framework. Soc Sci Med.

[103] Stroebe M, Schut H, Stroebe W (2007). Health outcomes of bereavement. Lancet.

[104] Stroebe M, Stroebe W, Schut H, Boerner K (2017). Grief is not a disease but bereavement merits medical awareness. Lancet.

[105] Reder EA, Serwint JR. Until the last breath: exploring the concept of hope for parents and health care professionals during a child's serious illness. Arch Pediatr Adolesc Med. 2009;163(7):653–7. 10.1001/archpediatrics.2009.87 19581549

[106] Wheeler I (2001). Parental bereavement: the crisis of meaning. Death Stud.

[107] Lathrop A, VandeVusse L. Continuity and change in mothers' narratives of perinatal hospice. J Perinat Neonat Nurs. 2011;25(1):21–31. 10.1097/JPN.0b013e3181fa9c60 21311266

[108] Björk M, Sundler AJ, Hallström I, Hammarlund K. Like being covered in a wet and dark blanket - parents' lived experiences of losing a child to cancer. Eur J Oncol Nurs. 2016;25:40–5. 10.1016/j.ejon.2016.08.007 27865251

[109] Stevenson M, Achille M, Liben S, Proulx MC, Humbert N, Petti A (2017). Understanding how bereaved parents cope with their grief to inform the services provided to them. Qual Health Res.

[110] Lobb EA, Kristjanson LJ, Aoun SM, Monterosso L, Halkett GKB, Davies A (2010). Predictors of complicated grief: a systematic review of empirical studies. Death Stud.

[111] Lord S, Moore C, Beatty M, Cohen E, Rapoport A, Hellmann J (2020). Assessment of bereaved caregiver experiences of advance care planning for children with medical complexity. JAMA Netw Open.

[112] Pritchard M, Srivastava DK, Okuma JO, Powell B, Burghen E, West NK, et al. Bereaved parents' perceptions about when their child's cancer-related death would occur. J Pain Symptom Manage. 2009;38(4):561–7. 10.1016/j.jpainsymman.2009.01.005 PMC2941143 19822277

[113] Wiener L, Tager J, Mack J, Battles H, Bedoya SZ, Gerhardt CA. Helping parents prepare for their child's end of life: a retrospective survey of cancer-bereaved parents. Pediatr Blood Cancer. 2020;67(2):e27993. 10.1002/pbc.27993 PMC8330433 31595653

[114] Heller KS, Solomon MZ (2005). Continuity of care and caring: what matters to parents of children with life-threatening conditions. J Pediatr Nurs.

[115] Sieg SE, Bradshaw WT, Blake S (2019). The best interests of infants and families during palliative care at the end of life: a review of the literature. Adv Neonatal Care.

[116] Baughcum AE, Fortney CA, Winning AM, Dunnells ZDO, Humphrey LM, Gerhardt CA (2020). Healthcare satisfaction and unmet needs among bereaved parents in the NICU. Adv Neonat Care.

[117] Sedig LK, Spruit JL, Paul TK, Cousino MK, Pituch K, Hutchinson R (2020). Experiences at the end of life from the perspective of bereaved parents: results of a qualitative focus group study. Am J Hosp Palliat Care.

[118] Cacciatore J, Thieleman K, Lieber AS, Blood C, Goldman R (2019). The long road to farewell: the needs of families with dying children. Omega: J Death Dying.

[119] Butler AE, Copnell B, Hall H. "Some were certainly better than others" - Bereaved parents' judgements of healthcare providers in the paediatric intensive care unit: a grounded theory study. Intensive Crit Care Nurs. 2018;45:18–24. 10.1016/j.iccn.2017.12.003 29290525

[120] Kreicbergs U, Valdimarsdóttir U, Onelöv E, Björk O, Steineck G, Henter JI (2005). Care-related distress: a nationwide study of parents who lost their child to cancer. J Clin Oncol.

[121] Lewis-Newby M, Clark JD, Butt WW, Dryden-Palmer K, Parshuram CS, Truog RD (2018). When a child dies in the PICU despite ongoing life support. Pediatr Crit Care Med.

[122] Sieg SE, Bradshaw WT, Blake S (2019). The best interests of infants and families during palliative care at the end of life: a review of the literature. Adv Neonat Care.

[123] Beernaert K, Lövgren M, Jeppesen J, Werlauff U, Rahbek J, Sejersen T, et al. parents' experiences of information and decision making in the care of their child with severe spinal muscular atrophy: a population survey. J Child Neurol. 2019;34(4):210–5. 10.1177/0883073818822900 30642225

[124] Fortney CA, Baughcum AE, Moscato EL, Winning AM, Keim MC, Gerhardt CA. Bereaved parents' perceptions of infant suffering in the NICU. J Pain Symptom Manage. 2020;59(5):1001–8. 10.1016/j.jpainsymman.2019.12.007 31837457

[125] Liben S, Papadatou D, Wolfe J (2008). Paediatric palliative care: challenges and emerging ideas. Lancet.

[126] Peng N-H, Liu H-L, Chen C-H, Bachman J (2012). Cultural practices and end-of-life decision making in the neonatal intensive care unit in Taiwan. J Transcult Nurs.

[127] Tan JS, Docherty SL, Barfield R, Brandon DH (2012). Addressing parental bereavement support needs at the end of life for infants with complex chronic conditions. J Palliat Med.

[128] Falkenburg JL, Tibboel D, Ganzevoort RR, Gischler SJ, van Dijk M (2018). The importance of parental connectedness and relationships with healthcare professionals in end-of-life care in the PICU. Pediatr Crit Care Med.

[129] Snaman JM, Kaye EC, Torres C, Gibson DV, Baker JN. Helping parents live with the hole in their heart: the role of health care providers and institutions in the bereaved parents' grief journeys. Cancer. 2016;122(17):2757–65. 10.1002/cncr.30087 27244654

[130] Butler AE, Copnell B, Hall H (2019). When a child dies in the PICU: practice recommendations from a qualitative study of bereaved parents. Pediatr Crit Care Med.

[131] Midson R, Carter B. Addressing end of life care issues in a tertiary treatment centre: lessons learned from surveying parents' experiences. J Child Health Care. 2010;14(1):52–66. 10.1177/1367493509347060 20207658

[132] Meert KL, Thurston CS, Thomas R (2001). Parental coping and bereavement outcome after the death of a child in the pediatric intensive care unit. Pediatr Crit Care Med.

[133] Mullen JE, Reynolds MR, Larson JS. Caring for pediatric patients' families at the child's end of life. Crit Care Nurse. 2015;35(6):46–55 quiz 6. 10.4037/ccn2015614 26628545

[134] Dutta O, Tan-Ho G, Choo PY, Ho AHY (2019). Lived experience of a child’s chronic illness and death: a qualitative systematic review of the parental bereavement trajectory. Death Stud.

[135] Cox SA (2004). Pediatric bereavement: supporting the family and each other. J Trauma Nurs.

[136] O’Malley PJ, Barata IA, Snow SK. Death of a child in the emergency department. Ann Emerg Med. 2014;64(1):102–5. 10.1016/j.annemergmed.2014.05.010 24951421

[137] Monterosso L, Kristjanson LJ (2008). Supportive and palliative care needs of families of children who die from cancer: an Australian study. Palliat Med.

[138] Melin-Johansson C, Axelsson I, Jonsson Grundberg M, Hallqvist F. When a child dies: parents' experiences of palliative care-an integrative literature review. J Pediatr Nurs. 2014;29(6):660–9. 10.1016/j.pedn.2014.06.009 25038375

[139] Santos MRD, Wiegand DL, Sá NN, Misko MD, Szylit R (2019). From hospitalization to grief: meanings parents assign to their relationships with pediatric oncology professional. Rev Esc Enferm USP.

[140] Price J, Jordan J, Prior L, Parkes J. Living through the death of a child: a qualitative study of bereaved parents' experiences. Int J Nurs Stud. 2011;48(11):1384–92. 10.1016/j.ijnurstu.2011.05.006 21640992

[141] Sedig LK, Spruit JL, Paul TK, Cousino MK, McCaffery H, Pituch K (2020). Supporting pediatric patients and their families at the end of life: Perspectives from bereaved parents. Am J Hosp Palliat Med.

[142] Clancy S, Lord B (2018). Making meaning after the death of a child. Child Adolesc Psychiatr Clin N Am..

[143] Lövgren M, Sejersen T, Kreicbergs U. Parents' experiences and wishes at end of life in children with spinal muscular atrophy types I and II. J Pediatr. 2016;175:201–5. 10.1016/j.jpeds.2016.04.062 27241662

[144] Haas F (2003). Bereavement care: seeing the body. Nurs Stand.

[145] Milstein JM (2003). Detoxifying death in the neonate: in search of meaningfulness at the end of life. J Perinatol.

[146] Lichtenthal WG, Sweeney CR, Roberts KE, Corner GW, Donovan LA, Prigerson HG (2015). Bereavement follow-up after the death of a child as a standard of care in pediatric oncology. Pediatr Blood Cancer.

[147] Mitchell S, Spry JL, Hill E, Coad J, Dale J, Plunkett A (2019). Parental experiences of end of life care decision-making for children with life-limiting conditions in the paediatric intensive care unit: a qualitative interview study. BMJ Open.

[148] Robert R, Zhukovsky DS, Mauricio R, Gilmore K, Morrison S, Palos GR. Bereaved parents' perspectives on pediatric palliative care. J Soc Work End Life Palliat Care. 2012;8(4):316–38. 10.1080/15524256.2012.732023 23194168

[149] Penson RT, Green KM, Chabner BA, Lynch TJ (2002). When does the responsibility of our care end: bereavement. Oncologist.

[150] Donovan LA, Wakefield CE, Russell V, Cohn RJ (2015). Hospital-based bereavement services following the death of a child: a mixed study review. Palliat Med.

[151] van der Geest IM, van den Heuvel-Eibrink MM, van Vliet LM, Pluijm SM, Streng IC, Michiels EM (2015). Talking about death with children with incurable cancer: perspectives from parents. J Pediatr.

[152] Adams G, Green A, Towe S, Huett A (2013). Bereaved caregivers as educators in pediatric palliative care: their experiences and impact. J Palliat Med.

[153] Bellali T, Papadatou D (2006). Parental grief following the brain death of a child: does consent or refusal to organ donation affect their grief?. Death Stud.

[154] Jennings V, Nicholl H (2014). Bereavement support used by mothers in Ireland following the death of their child from a life-limiting condition. Int J Palliat Nurs.

[155] O’Connor K, Barrera M. Changes in parental self-identity following the death of a child to cancer. Death Stud. 2014;38(6–10):404–11. 10.1080/07481187.2013.801376 24666147

[156] Meert KL, Thurston CS, Briller SH. The spiritual needs of parents at the time of their child's death in the pediatric intensive care unit and during bereavement: a qualitative study. Pediatr Crit Care Med. 2005;6(4):420–7. 10.1097/01.PCC.0000163679.87749.CA 15982428

[157] Thornton R, Nicholson P, Harms L (2019). Scoping review of memory making in bereavement care for parents after the death of a newborn. J Obstet Gynecol Neonatal Nurs.

[158] Côté-Arsenault D, Denney-Koelsch EM, McCoy TP, Kavanaugh K (2019). African American and Latino bereaved parent health outcomes after receiving perinatal palliative care: a comparative mixed methods case study. Appl Nurs Res.

[159] Coelho A, de Brito M, Barbosa A (2018). Caregiver anticipatory grief: phenomenology, assessment and clinical interventions. Curr Opin Support Palliat Care.

[160] Butler AE, Hall H, Copnell B (2018). Becoming a team: the nature of the parent-healthcare provider relationship when a child is dying in the pediatric intensive care unit. J Pediatr Nurs.

[161] Seecharan GA, Andresen EM, Norris K, Toce SS. Parents' assessment of quality of care and grief following a child's death. Arch Pediatr Adolesc Med. 2004;158(6):515–20. 10.1001/archpedi.158.6.515 15184212

[162] Drew D, Goodenough B, Maurice L, Foreman T, Willis L (2005). Parental grieving after a child dies from cancer: is stress from stem cell transplant a factor?. Int J Palliat Nurs.

[163] Kacel E, Gao X, Prigerson HG (2011). Understanding bereavement: what every oncology practitioner should know. J Support Oncol.

[164] Abib El Halal GM, Piva JP, Lago PM, El Halal MG, Cabral FC, Nilson C, et al. Parents' perspectives on the deaths of their children in two Brazilian paediatric intensive care units. Int J Palliat Nurs. 2013;19(10):495–502. 10.12968/ijpn.2013.19.10.495 24162280

[165] Snaman JM, Kaye EC, Levine DR, Cochran B, Wilcox R, Sparrow CK (2017). Empowering bereaved parents through the development of a comprehensive bereavement program. J Pain Symptom Manage.

[166] Meert KL, Eggly S, Pollack M, Anand KJ, Zimmerman J, Carcillo J, et al. Parents' perspectives on physician-parent communication near the time of a child's death in the pediatric intensive care unit. Pediatr Crit Care Med. 2008;9(1):2–7. 10.1097/01.PCC.0000298644.13882.88 PMC3198033 18477906

[167] Denhup C. Bereavement care to minimize bereaved parents' suffering in their lifelong journey towards healing. Appl Nurs Res. 2019;50:151205. 10.1016/j.apnr.2019.151205 31677927

[168] Bennett RA, LeBaron VT (2019). Parental perspectives on roles in end-of-life decision making in the pediatric intensive care unit: an integrative review. J Pediatr Nurs.

[169] Sperandeo DDS. Post-decisional conflict in selecting cancer treatments: Perception of information disclosure may influence decisional conflict, decisional regret, and self-acceptance in bereaved parents of children with cancer. Dissertation Abstracts Int: Sect B: Sci Eng. 2020;81(4-B):No Pagination Specified.

[170] Levick J, Fannon J, Bodemann J, Munch S (2017). NICU Bereavement Care and Follow-up Support for Families and Staff. Adv Neonat Care.

[171] Goldstein RD, Lederman RI, Lichtentha WG, Morris SE, Human M, Elliott AJ, et al. The grief of mothers after the sudden unexpected death of their infants. Pediatrics. 2018;141(5). 10.1542/peds.2017-3651 PMC6173829 29712764

[172] Morris AT, Gabert-Quillen C, Friebert S, Carst N, Delahanty DL (2016). The indirect effect of positive parenting on the relationship between parent and sibling bereavement outcomes after the death of a child. J Pain Symptom Manage.

[173] Lykke C, Ekholm O, Schmiegelow K, Olsen M, Sjøgren P (2019). Anxiety and depression in bereaved parents after losing a child due to life-limiting diagnoses: a danish nationwide questionnaire survey. J Pain Symptom Manage.

[174] Dias N, Brandon D, Haase JE, Tanabe P. Bereaved parents' health status during the first 6 months after their child's death. Am J Hosp Palliat Care. 2018;35(6):829–39. 10.1177/1049909117744188 29202599

[175] McCarthy MC, Clarke NE, Ting CL, Conroy R, Anderson VA, Heath JA (2010). Prevalence and predictors of parental grief and depression after the death of a child from cancer. J Palliat Med.

[176] Meyer EC, Burns JP, Griffith JL, Truog RD (2002). Parental perspectives on end-of-life care in the pediatric intensive care unit. Crit Care Med.

[177] Sirki K, Saarinen-Pihkala UM, Hovi L (2000). Coping of parents and siblings with the death of a child with cancer: death after terminal care compared with death during active anticancer therapy. Acta Paediatr.

[178] deCinque N, Monterosso L, Dadd G, Sidhu R, Lucas R (2004). Bereavement support for families following the death of a child from cancer: practice characteristics of Australian and New Zealand paediatric oncology units. J Paediatr Child Health.

[179] Leemann T, Bergstraesser E, Cignacco E, Zimmermann K. Differing needs of mothers and fathers during their child's end-of-life care: Secondary analysis of the "paediatric end-of-life care needs" (PELICAN) study. BMC Palliative Care. 2020;19(1). 10.1186/s12904-020-00621-1 PMC7405340 32753031

[180] Currie ER, Christian BJ, Hinds PS, Perna SJ, Robinson C, Day S (2016). Parent perspectives of neonatal intensive care at the end-of-life. J Pediatr Nurs.

[181] Broden EG, Deatrick J, Ulrich C, Curley MAQ. Defining a "good death" in the pediatric intensive care unit. Am J Crit Care. 2020;29(2):111–21. 10.4037/ajcc2020466 PMC11288184 32114610

[182] Abraham A, Hendriks MJ. "You can only give warmth to your baby when it's too late": parents' bonding with their extremely preterm and dying child. Qual Health Res. 2017;27(14):2100–15. 10.1177/1049732317721476 28758538

[183] Butler AE, Hall H, Copnell B (2018). The changing nature of relationships between parents and healthcare providers when a child dies in the paediatric intensive care unit. J Adv Nurs.

[184] Cortezzo DE, Sanders MR, Brownell EA, Moss K (2015). End-of-life care in the neonatal intensive care unit: experiences of staff and parents. Am J Perinatol.

[185] Haig A, Dozier M (2003). BEME Guide No. 3: Systematic searching for evidence in medical education - Part 2: Constructing searches. Med Teach.

[186] Gordon M, Gibbs T (2014). STORIES statement: Publication standards for healthcare education evidence synthesis. BMC Med.

[187] Garcini LM, Brown RL, Chen MA, Saucedo L, Fite AM, Ye P, et al. Bereavement among widowed Latinos in the United States: a systematic review of methodology and findings. Death Stud. 2021;45(5):342–53. 10.1080/07481187.2019.1648328 PMC7076907 31402785

[188] D’Agostino NM, Berlin-Romalis D, Jovcevska V, Barrera M. Bereaved parents' perspectives on their needs. Palliat Support Care. 2008;6(1):33–41. 10.1017/S1478951508000060 18282343

[189] Macdonald ME, Liben S, Carnevale FA, Rennick JE, Wolf SL, Meloche D, et al. Parental perspectives on hospital staff members' acts of kindness and commemoration after a child's death. Pediatrics. 2005;116(4):884–90. 10.1542/peds.2004-1980 16199697

[190] van der Geest IMM, Darlington A-SE, Streng IC, Michiels EMC, Pieters R, van den Heuvel-Eibrink MM. Parents' experiences of pediatric palliative care and the impact on long-term parental grief. J Pain Symptom Manage. 2014;47(6):1043–53. 10.1016/j.jpainsymman.2013.07.007 24120185

[191] Giorgali S. Bereaved parents’ needs regarding hospital based bereavement care after the death of a child to cancer. Death Stud. 2020:1–9. Advance online publication. 10.1080/07481187.2020.1824202 32972331

[192] Lichtenthal WG, Sweeney CR, Roberts KE, Corner GW, Donovan LA, Prigerson HG (2015). Bereavement follow-up after the death of a child as a standard of care in pediatric oncology. Pediatr Blood Cancer.

[193] Snaman JM, Kaye EC, Torres C, Gibson D, Baker JN (2016). Parental grief following the death of a child from cancer: the ongoing odyssey. Pediatr Blood Cancer.

[194] Jensen J, Weng C, Spraker-Perlman HL (2017). A provider-based survey to assess bereavement care knowledge, attitudes, and practices in pediatric oncologists. J Palliat Med.

[195] Alsuwaigh R (2015). How do English-speaking cancer patients conceptualise personhood?. Ann Acad Med Singap.

[196] Wijngaards-de Meij L, Stroebe M, Schut H, Stroebe W, van den Bout J, van der Heijden P (2005). Couples at risk following the death of their child: predictors of grief versus depression. J Consult Clin Psychol.

[197] Maass U, Hofmann L, Perlinger J, Wagner B. Effects of bereavement groups–a systematic review and meta-analysis. Death Stud. 2020:1–11. Advance online publication. 10.1080/07481187.2020.1772410 32501773

[198] Sikstrom L, Saikaly R, Ferguson G, Mosher PJ, Bonato S, Soklaridis S (2019). Being there: A scoping review of grief support training in medical education. PLoS ONE.

[199] Morris SE, Block SD (2015). Adding value to palliative care services: the development of an institutional bereavement program. J Palliat Med.

[200] Contro N, Sourkes BM. Opportunities for quality improvement in bereavement care at a children's hospital: assessment of interdisciplinary staff perspectives. J Palliat Care. 2012;28(1):28–35. 22582469

