# Supplementary material for: Parental bereavement – impact of death of neonates and children under 12 years on personhood of parents: a systematic scoping review
Source: BMC Palliat Care. 2021 Sep 4;20:136. doi: 10.1186/s12904-021-00831-1 (PMC8418708; doi:10.1186/s12904-021-00831-1)
Supplement: Supplementary file 1 — Additional file 1. Search Strategy. [file 12904_2021_831_MOESM1_ESM.docx]

| ***Search*** | ***Query*** |
| --- | --- |
| *#1* | “Personhood”[MeSH] OR Personhood[tiab] OR selfhood[tiab] OR self[tiab] OR character[tiab] OR individuation[tiab] OR individual[tiab] OR self-awareness[tiab] OR “self awareness”[tiab] OR “Bereavement”[MeSH] OR bereave*[tiab] OR “Grief”[MeSH] OR griev*[tiab] OR mourn*[tiab] OR grief[tiab] OR “Attitude to Death”[Mesh] OR "Adaptation, Psychological"[Mesh] OR "Social Adjustment"[MeSH] OR “Mother-Child Relations”[MeSH] OR “Mothers/psychology”[MeSH] OR “Social Support”[MeSH] OR “Self Care/psychology”[MeSH] OR “Parent-Child Relations”[MeSH] OR “Personality”[MeSH] OR personality[tiab] OR “Consciousness”[MeSH] OR consciousness[tiab] OR “relational personhood”[tiab] OR “Human Rights”[MeSH] OR “human rights”[tiab] OR “human dignity”[tiab] OR “Cognition”[MeSH] OR cognition[tiab] OR “cognitive function”[tiab] OR “self awareness”[tiab] OR self-awareness[tiab] OR “Awareness”[MeSH] OR awareness[tiab] OR “personal beliefs”[tiab] OR “personal belief”[tiab] OR “personal values”[tiab] OR “familial roles”[tiab] OR “familial role”[tiab] OR “familial expectation*”[tiab] OR “filial duties”[tiab] OR “filial duty”[tiab] OR “societal expectations”[tiab] OR “societal expectation”[tiab] OR “religious mores”[tiab] OR “self expression”[tiab] |
| *#2* | "Palliative Care"[Mesh] OR "Palliative Medicine"[Mesh] OR Palliative[tiab] OR "Hospice and Palliative Care Nursing"[Mesh] OR "Hospice Care"[Mesh] OR "Terminal Care"[Mesh] OR “palliative care”[tiab] OR “hospice care”[tiab] OR “terminal care”[tiab] OR “terminal illness”[tiab] OR “terminally ill”[tiab] OR “end of life”[tiab] OR end-of-life[tiab] |
| *#3* | “Death”[Mesh]  OR “Mortality”[Mesh] OR "Sudden Infant Death"[Mesh] OR "Infant Death"[Mesh] OR "Perinatal Death"[Mesh] OR death[tiab] OR deaths[tiab] OR mortality[tiab] |
| *#4* | Infant[MeSH] OR infant[tiab] OR infant[tiab] OR infancy[tiab] OR newborn[tiab] OR newborn[tiab] OR baby[tiab] OR babies[tiab] OR Child[MeSH] OR child[tiab] OR schoolchild*[tiab] OR school age*[tiab] OR preschool*[tiab] OR kid[tiab] or kids[tiab] OR toddler[tiab] OR toddlers[tiab] OR boy[tiab] OR boys[tiab] OR girl[tiab] OR girls[tiab] OR Minors[MeSH] OR minor[tiab] OR minors[tiab] OR Puberty[MeSH] OR puberty*[tiab] OR pubescen*[tiab] OR prepubescen*[tiab] OR Pediatrics[MeSH] OR paediatric[tiab] OR paediatrics[tiab] OR pediatric[tiab] OR pediatrics[tiab] OR paediatrician[tiab] OR paediatricians[tiab] OR pediatrician[tiab] OR pediatricians[tiab] OR “Nursery school*”[tiab] OR kindergar*[tiab] OR “primary school*”[tiab] OR “elementary school*”[tiab] OR “Pediatric Emergency Medicine”[MeSH] OR “Pediatric Emergency Medicine”[Tiab] OR “Child, Hospitalized”[MeSH Terms] OR “Child, Hospitalized”[tiab] |
| *#5* | Combined #1, #2, #3 and #4 |
