# Supplementary material for: Parental bereavement – impact of death of neonates and children under 12 years on personhood of parents: a systematic scoping review
Source: BMC Palliat Care. 2021 Sep 4;20:136. doi: 10.1186/s12904-021-00831-1 (PMC8418708; doi:10.1186/s12904-021-00831-1)
Supplement: Supplementary file 2 — Additional file 2. Tabulated Summaries. [file 12904_2021_831_MOESM2_ESM.docx]

| **Authors/Year** | **Article title** | **Type of study** | **MERSQI** | **COREQ** | **Study Aim** | **Methodology** | **Key findings** | **Proposed solutions/ conclusions** |
| --- | --- | --- | --- | --- | --- | --- | --- | --- |
| Abdel Razq, N.M., & Al-Gamal, E., 2018 | Maternal Bereavement: Mothers' Lived Experience of Losing a Newborn Infant in Jordan | *Qualitative* |  | 23 | “The aim of this study was to understand bereavement and its associated meanings as lived and experienced by the mothers who lost their newborn infants in the intensive care units of hospitals in Jordan.” | “Data were generated using semi structured face-to-face interviews with 12 mothers whose neonates died after being admitted to the NICUs for at least 24 hours at 2 hospitals in Jordan (1 private and 1 public). A qualitative phenomenological approach was used for data analysis.”  “The newborns" stay duration in the NICU ranged from 1 day to 3 weeks. Reasons for the child"s NICU stay and death varied between prematurity, congenital anomalies, congenital sepsis, severe respiratory distress, and/or asphyxia.” | Three main themes emerged from the analysis:  (1) Longing and grieving, as natural emotional responses to the loss;  (2) Adaptive work of coping, as the mothers internalized meanings to cope with their loss:   - Many mothers in an attempt to accept the reality of their newborn's death, searched for a larger meaning or rationale (i.e. devine meanings, spirituality, spiritual beliefs) - Spiritual values are embedded in the cultural and social values of the Jordanian community: Religious beliefs such as the importance of patience, divine testing of faith through calamity, and that deceased newborns are ‘‘birds in heaven’’ were repeated. - Being socially or professionally active - Friends were a huge support, as well as family - Lack of support from HCPs: not given enough counseling time.   (3) Moving forward but with a scar, as the mothers moved on with their lives while they carried the unforgettable memories of the newborns" death experience.   - Willingness to 'let go' of the deceased newborns' belongings and begin preparing for subsequent pregnancy - Concerned about the health of their next newborns, some fear for the next pregnancy even though they hoped for better experiences | “Mothers continuously confirmed the individuality of deceased newborns, despite often extremely short lives lived away from their arms in the ICU.” E.g. referred neonates to given names, and were buried with a name even if they died before parents had the time to pick a name.  “Mother commented on HCPs - They did the tasks, only! Taking [blood] pressure, administering medications, the things.”  Mothers whose newborns died in the NICU perceived pressure from family members to have another child, and this pressure was more common for mothers whose infants died in the NICU, rather than mothers who have lost older children. |
| Abib El Halal, G.M., et al., 2013 | Parents’ perspectives on the deaths of their children in two Brazilian paediatric intensive care units | *Qualitative* |  | 18 | “The PICU’s handling of the dying process can affect how much suffering is felt. Palliative care is a priority to promote a good death and minimize the child’s and the family’s suffering. Halal seeks to evaluate the quality of care offered to terminally ill children and their families in the last days of life in 2 Brazilian PICUs from the parents’ perspectives.” | Qualitative exploratory study. Parents of 9 of the 38 children who died in the two Brazilian PICUs attended the interview.  Thematic analysis sought data into six categories:   1. Communication with the attending physicians 2. Quality of care 3. Quality of communication in the PICU 4. Parental participation in the decision making process 5. Moments surrounding the child’s death   Feelings regarding the experience of being included in the research | 1. Quality of communication was low; medical staff frequently using technical terms which limited understanding. Unable to understand the seriousness of the situation or why certain procedures were performed. 2. Parental participation in decision making was scarce and decisions were based on the medical perspective. 3. Reports of uncompassionate attitudes from the medical staff 4. Last moments of child’s lives lacked privacy and a peaceful environment for farewells. Excessive use of technologies in the PICU. | Uncovering of many deficiencies in the care provided to parents in the PICUs. Great need for changes in practice.  Palliative care should not end at the time of death but should be extended to the family in the post-death period. |
| Andrea Abraham & Manya J. Hendriks 2017 | “You Can Only Give Warmth to Your Baby When It’s Too Late”: Parents’ Bonding With Their Extremely Preterm and Dying Child | *Qualitative* |  | 16 | “This article illustrates the perspective of parents whose extremely premature baby died within a few hours or days after birth. It focuses on how parents experience the dying trajectory of their extremely premature babies in the NICU, how they experience parenthood, and how HCPs can facilitate parent–child bonding in the very short time available. It presents data from a qualitative study on end-of-life decision making in extremely pre-term infants at the limit of viability. Our study was secondary to a larger quantitative survey on end-of-life decisions in extremely preterm infants. Our task was to shed light on the parental perspective, whereas the other studies forming part of the survey focused on the perspec-tives of society, of NICU HCPs, and on ethical questions arising from the results.” | “This study used a methodological approach based on symbolic interactionism with its focus on the reconstruction of subjective meaning. For data collection, we chose an interview design with ethnographic aspects (temporary field stays, minimal observations). We conducted a retrospective study (and not, for instance, a prospective ethnographic study) due to the assumed mourning process of parents. During our informal talks in the preparatory field stays, we were advised not to conduct interviews with parents who had only recently lost their child.” | “This study on end-of-life decisions in extremely preterm babies shows that the parents under study experience a multitude of stressors due to the immediate separation after birth, the alienating setting of the neonatal intensive  care unit (NICU), the physical distance to the child, medical uncertainties, and upcoming decisions. Even though they are considered to be parents (assigned parenthood), they cannot act as primary caregivers. Instead, they depend on  professional instructions for access and care. Embodied parenthood can be experienced only at the end-of-life, that is, during the dying trajectory and after the child’s death. Professionally supporting parents during this compressed process (from assigned and distant to embodied parenthood) contributes fundamentally to their perception of being  a family and supports their mourning. This calls for the further establishment of palliative and bereavement care concepts in neonatology” | “In conclusion, our study illustrates that parents of extremely preterm babies suffered from unpreparedness of becoming parents: They were considered parents (assigned parenthood) with the birth of their baby, but to actually feel like parents they needed to go through a pro-cess of biological and psychosocial bonding. In our sample, child–parent embodiment through holding, touching, smelling, caring, and protecting barely occurred before dying (distant parenthood). Thus, caring for their dying and deceased child enabled parents to become parents in an embodied sense. Even though this trajectory can be very short in time, it is of crucial importance that parents receive the professional support needed to experience embodied parenthood which is grounded on palliative and bereavement care concepts elaborated for neonatology.” |
| Annie Rini, Lillia Loriz, 2007 | Anticipatory Mourning in Parents With a Child Who Dies While Hospitalized | *Qualitative* |  | 18 | “Even when prognosis is poor and death appears imminent, care of the dying child typically focuses on achieving cure. Parents are often ill-prepared to cope with the grief they experience as their child is dying. Anticipatory mourning allows time to begin grief work prior to the death of a loved one.” | “An exploratory design was used to determine the presence and the role of anticipatory mourning, and to describe the themes expressed by parents.  The study used a focused, guided, semi structured, in-depth interviews, was used to collect data. A set of open-ended questions, developed from a review of the literature and from the researchers’ own personal experience in this field, was used to guide the interviews.  A purposive sampling technique was used to recruit  11 parents who sustained the death of a child during the child’s hospitalization, thus comprising a homogeneous subgroup.” | “The major themes that emerged as facilitating or impeding anticipatory mourning are as follows: (1) giving of information to parents; (2) the impact of the attitudes and actions of health care professionals; (3) physical presence with the dying child; (4) the location of the child’s death; (5) issues of hospital policy, procedures, process, and rules; and (6) the existence of anticipatory mourning and its relationship with bereavement.  Parents’ descriptions of their experiences surrounding the death of their child reveal an environment and a health care team that are often ill-prepared to deal with the impending death of a child.  Also described are instances that reflect a compassionate process that positively affects the experience while facilitating appropriate grief work.” | The study offered recommendations for health care professionals that may assist parents in coping with the death of their child by looking the themes from the interviews.  1. The first of these is the theme of desiring information, particularly detailed information, that would serve to assist parents in their attempts to grasp and understand the events that are occurring.  2. The need for a health care member to function in the role of coach, who can assist, describe, and guide parents through what is involved and anticipated in the dying process, is a strong theme  expressed by many parents.  3. Being physically present and participating in the care of the child leading up to and following the death were desired and considered important for study parents.  4. The actual setting where death occurs appeared to influence the perception and experience of parents in this study. There is a need to be in a comfortable soothing environment where there is freedom to grieve and to complete tasks deemed important to be performed by parents as their child was dying. |
| Anscombe, Elizabeth 2008 | The dichotomy of containing trauma amidst joy: New life and neonatal death; the experience of working with the parents of twins on the NICU | *Case report* |  |  | This paper describes the work with a family of premature twins in a neonatal intensive care unit (NICU), where one twin was born seriously ill and died within a few days. An overview of literature related to the birth of a sick premature baby, trauma and mourning with twins, and the impact of loss on the surviving twin, forms the first section. | Case Report | Informed by psychoanalytic theory, the author explores the painful work with the parents through detailed observation, noting particularly the difference between the father’s capacity to stay in touch with the pain of loss and mother’s denial and preoccupation with the healthy twin. | NIL |
| Anthony Lathrop & Leona Vande Vusse 2011 | Continuity and Change in Mothers’ Narratives of Perinatal Hospice | *Qualitative* |  | 14 | (1) To broadly explore the experiences of women who chose to continue pregnancies affected by lethal fetal diagnoses and  (2) To develop knowledge useful to nurses and other healthcare professionals who provide perinatal hospice (PH) care. | “Design: Qualitative descriptive study using narrative analysis. Participants: Fifteen women who learned during their pregnancies of a lethal fetal diagnosis and chose to continue the affected pregnancies. Method: Participants’ stories of their PH experiences were recorded in face-to-face interviews. A qualitative approach using narrative analysis was used to identify themes and develop suggestions for care.” | “The element of time was prevalent in mothers’ stories. Some aspects of mothers’ experiences continued, particularly feelings of love and connection to their babies. Mothers also reported evolving changes in their thoughts and feelings. Personal changes such as increased compassion, faith, and strength were frequently mentioned. Mothers described transient phases of highs and lows. Drawing personal meanings or life lessons was the main way mothers connected their experiences to their present lives.” | Mothers’ descriptions of their experiences can enhance nurses’ understanding of perinatal loss.  Development of established care practices. |
| Armentrout, D., 2009 | Living with Grief Following Removal of Infant Life Support : Parents’ Perspectives | *Qualitative* |  | 12 | “Research findings reported in the literature about making life and death decisions for critically ill infants in the neonatal ICU (NICU) focus primarily on the experiences of health care providers and the ethical dilemmas surrounding these decisions. Fewer studies focus on parents’ experiences in making decisions about discontinuing life support for the infant, and even fewer address what life is like for parents following the death of the infants. Therefore the author conducted a qualitative study as part of her doctoral requirements that explored with parents how life support decisions were made for their infants, the roles they had in the decision-making process, and how the decisions and subsequent deaths of their infants influence their everyday lives. The results of that qualitative investigation, Holding a Place: A Grounded Theory of Parents Bringing Their Infant Forward in Their Daily Lives Following the Removal of Life Support and Subsequent Infant Death, have been described elsewhere. This article expands on the concepts identified by parents as factors in their decision making and on the facilitators and barriers the parents faced, and continue to face, in their grieving process.” | “Data in this grounded theory study were narrative interviews. Each participant was asked the same opening question: ‘‘Tell me about your son/your daughter.’’ Parents then were asked to describe what it was like to realize that their infant was not going to survive. Parents were asked to describe what their lives were like immediately after their infant’s death and how their lives are now, without the infant. Parents also were asked about their current feelings regarding their decisions and if, in retrospect, they would do anything differently. Participants were encouraged to share their thoughts and memories of the infant, what the infant’s brief presence in their lives meant to them, and how the decisions they made influenced the ways their lives have evolved. Either during or at the end of each interview, parents were asked what advice they have for parents currently involved in facing such a decision. Finally, each participant was asked what he or she believed was important to share about their experiences that had not been addressed.” | The following themes were identified:   - Making the decision - Things they would have changed - Healthcare providers - Initial shock - Differences in grief between mothers and fathers - Family and friends - Feelings of isolation - Remembering activities - Moving forward - Altered perspectives of life - Spiritual/religious perspectives | “This qualitative work provides unique insight into parents’ perspectives of grief as they moved forward with their lives following the removal of life support and subsequent death of their infant. In addition it illuminates how parents’ grief and loss becomes incorporated into their ongoing lives.” |
| Balkin, E.M., et al. (2015) | Physician and parent perceptions of prognosis and end of life experience in children with advanced heart disease | *Quantitative* | 10 |  | “Little is known about how physician and parent perspectives compare regarding the prognosis and end-of-life (EOL) experience of children with advanced heart disease (AHD). Balkin seeks to describe and compare parent and physician perceptions regarding prognosis and EOL experience in children with AHD.” | Cross-sectional survey study  “Bereaved parents and cardiologists of children with primary cardiac diagnoses who died in a tertiary care pediatric hospital between January 2007 and December 2009.”  “33 of 78 parents approached completed survey (42%). Of the 33 parents, 31 cardiologists completed physician survey (94%). Final sample size was 31.” | “Nearly half of parents and physicians felt that patients suffered ‘a great deal,’ ‘a lot,’ or ‘somewhat’ at EOL, but there was no agreement between them.”  “At diagnosis, parents more often expected complete repair and normal lifespan while the majority of physicians expected shortened lifespan without normal quality of life.  Parents who expected complete repair with normal life were more likely to report ‘a lot’ of suffering at EOL”   - In 43% of cases, physicians reported that the parents were prepared for the way in which their child died, while the parents reported feeling unprepared. | “Physicians tend to overestimate the degree of parent preparedness for their child’s death.” |
| Baughcum, A. E., et al., 2020 | Healthcare Satisfaction and Unmet Needs Among Bereaved Parents in the NICU | *Mixed method* | 13 | 7 | “Learning directly from bereaved parents about their experiences in the neonatal intensive care unit (NICU) can improve services at end-of-life (EOL) care. Parents who perceive that their infant suffered may report less satisfaction with care and may be at greater risk for distress after the death. Despite calls to improve EOL care for children, limited research has examined the EOL experiences of families in the NICU.”  “We examined parent perceptions of their infant’s EOL experience (eg, symptom burden and suffering) and satisfaction with care in the NICU.” | “Forty-two mothers and 27 fathers (representing 42 infants) participated in a mixed-methods study between 3 months and 5 years after their infant’s death (mean = 39.45 months, SD = 17.19). Parents reported on healthcare satisfaction, unmet needs, and infant symptoms and suffering in the final week of life.” | “Parents reported high levels of healthcare satisfaction, with relative strengths in providers’ technical skills and inclusion of the family.”  “Greater perceived infant suffering was associated with lower healthcare satisfaction and fewer well-met needs at EOL.”  “Parents’ understanding of their infant’s condition, emotional support, communication, symptom management, and bereavement care were identified as areas for improvement.” | “Results can help shape staff education initiative, program development, and research initiatives within the NICU setting to improve service delivery.”  We must attempt to effectively address infant distress, specifically symptoms and suffering.  Healthcare providers need to do more to prepare families by screening for preexisting mental health needs at admission and help with developing coping strategies; Mental health professionals in the NICU and palliatve care team are key.  “Parents have also reported feeling alone and abandoned after leaving the hospital and they want to maintain connections to staff members.” |
| Beernaert, K., et al., 2018 | Parents' Experiences of Information and Decision Making in the Care of Their Child With Severe Spinal Muscle Atrophy: A Population Survey | *Quantitative* | 13 |  | “Study aims to assess the experiences and wishes of parents of children with severe spinal muscular atrophy regarding information and decision-making throughout the course of the illness.” | “A full population survey, conducted in 2015, among parents of children with severe spinal muscular atrophy who were born in Denmark between January 1, 2003, and December 31, 2013.”  Study-specific questionnaire with items about experiences and wishes concerning the provision of information about diagnosis, treatment, and end-of-life care.  “Among the 47 parents that were identified, 34 parents of 21 children participated. Eleven of them were nonbereaved and 23 were bereaved parents.” | “All parents stated that health care staff did not take any decisions without informing them. A proportion of parents indicated that they were not informed about what spinal muscular atrophy entails (32%), possible treatment options (18%), or the fact that their child would have a short life (26%) or that death was imminent (57%). Most of the bereaved parents who had wishes concerning how and where their child would pass away had their wishes fulfilled.”  Healthcare staff did not take treatment decisions without parents being informed; however there is still a lack of information given to the parents. | “Not understanding the diagnosis and prognosis of your child might influence a patient's decisions and wishes and make it difficult to plan care. Predicting prognosis and imminent death in spinal muscular dystrophy however is difficult.” |
| Bennett, R. A. & V. T. LeBaron, 2019 | Parental Perspectives on Roles in End-of-Life Decision Making in the Pediatric Intensive Care Unit: An Integrative Review | *Descriptive (Review)* |  |  | “Little is known about how parents perceive their role or the role of health care providers (HCPs) during end-of-life decision making (EOL DM) in the context of the pediatric intensive care unit (PICU).” | “The authors searched CINAHL, PubMed, OvidMedline, Web of Science, Social Science Database, PsycINFO, and Google scholar for English language studies performed in the United States related to parental perception of parental or HCP roles in EOL DM in the PICU since 2008. Eleven studies of parents and health care providers (HCPs) of critically ill children in the PICU and/or receiving inpatient pediatric palliative care, and bereaved parents of PICU patients.” | “Most parents reported belief that EOL DM is within the domain of parental role, a minority felt it was a physician's responsibility. Parental EOL DM is rooted more firmly in emotion and perception and a desire to be a ‘good parent’ to a child at EOL in the way they see fit than HCP recommendations or ‘medical facts’. Parents need HCPs to treat them as allies, communicate well, and be trustworthy.” | “Role conflict may exist between parents and HCPs who are prioritizing different attributes of the parental role. Both providers and parents have the ‘best interest’ of the child at heart; the issue is that ‘best interest’ is often personally defined and informed by the role of the individual.”  “Nurses are in sustained closer physical proximity to patients and their families and spend more time with them on a day-today basis than any other member of the team. Some studies show that parents rank nurses higher as sources of support and understanding during EOL DM than friends, extended family, spiritual directors, or instinct. However, The role of the nurse in support of parental role in the PICU is not well-elucidated in the extant literature.” |
| Broden, E. G., et al., 2020 | Defining a "Good Death" in the Pediatric Intensive Care Unit. | *Descriptive (Review)* |  |  | “Societal attitudes about end-of-life events are at odds with how, where, and when children die. In addition, parents’ ideas about what constitutes a “good death” in a pediatric intensive care unit vary widely. Many societal conclusions about a “good death” hinge on the assumption that the dying person is an adult who has lived a complete life and whose wishes are well established.”  “Objective: To synthesize parents’ perspectives on end-of life care in the pediatric intensive care unit in order to define the characteristics of a good death in this setting from the perspectives of parents.” | “A concept analysis was conducted of parents’ views of a good death in the pediatric intensive care unit. Empirical studies of parents who had experienced their child’s death in the inpatient setting were identified through database searches.” | “Because of the profound and enduring nature of parents’ grief, integrating their perspectives into the definition of a good death in the PICU is imperative.”  “Central to the concept of a good death for a child is the overarching theme of being a good parent—that is, doing what is best for the child and ensuring that he or she feels loved, especially at the end of life.”  “It is essential to establish an ethical framework in which the child, the parents, and the interprofessional team continuously identify and discuss shared goals of care within the context of sociocultural and familial preferences.” | “A supportive environment built on ethical concordance and centered around the parent-child relationship can foster compassionate caregiving to achieve the characteristics of a good death.”  “The parent perspectives and exemplar cases suggest necessary conditions—termed antecedents in concept analysis—that must be met in order to achieve a good death in the PICU. These antecedents are preparation, mutuality, communication, and resource mobilization or use.”  “A good death in the PICU influences the bereavement process, adaptation, and continued bonds and memories, which may evolve over time in keeping with the oscillatory nature of grief.” |
| Butler, A. E., et al., 2018 | Gradually Disengaging: Parent-Health care Provider Relationships After a Child's Death in the Pediatric Intensive Care Unit | *Qualitative* |  | 21 | “When a child dies in the intensive care unit, many bereaved parents want relationships with their child’s health care staff to continue in the form of follow-up care. However, the nature of these relationships and how they change across the parents’ bereavement journey is currently unknown. This article explores early and ongoing relationships between parents and health care staff when a child dies in intensive care.” | “Constructivist grounded theory methods were used to recruit 26 bereaved parents from four Australian pediatric intensive care units into the study. Data were collected via audio recorded, semi structured interviews and analyzed using the constant comparative methods and theoretical memoing.” | “Findings show that these relationships focus on Gradually disengaging, commonly moving through three phases after the child dies: Saying goodbye, Going home, and Seeking supports.”  “Saying goodbye describes the first phase of the relationship between parents and health care providers (predominantly nurses and social workers), occurring close to the time of a child’s death in PICU.”  “Once parents had said goodbye to their child, they began the process of Going home. The relationship between parents and health care providers again adapted to the parents’ changing needs, focusing on supporting them to leave the hospital without their child. Parents often did not know the procedures associated with leaving the hospital”  “Finally, Seeking supports described the parents’ need for longer term ongoing support, typically with someone with whom they had already developed a relationship in the PICU. For most parents, their continued relationship with health care providers centered around this need, with support gradually withdrawn over a number of months.” | “Our findings suggest that most parents want these relationships to continue through the dying phase and during their initial bereavement period, though the perceived purpose of the relationship changes. Instead of desiring teamwork and collaboration, parents rely on their relationships to support them as they say goodbye and move into their bereavement journey. The continued presence or absence of these relationships has impacts well beyond the actual hospitalization period of the child, often influencing the parents’ entire perception of their child’s death.”  “In addition to needing support to say goodbye, parents in our study also desired ongoing close contact with health care providers as they attempted to leave the hospital. They frequently wanted a chance to debrief, support to pack their child’s belongings, and assistance to leave the PICU and go home. Without it, parents may feel a sense of immediate abandonment, as if staff had simply moved on to other patients. This desire for a “transition period” between PICU care and home is a new finding; a concept that has not yet been explored within the PICU end-of-life care literature.” |
| Butler, A. E., et al., 2019 | When a Child Dies in the PICU: Practice Recommendations From a Qualitative Study of Bereaved Parents | *Qualitative* |  | 11 | “Around the world, the PICU is one of the most common sites for hospitalized children to die. Although ensuring the best possible care experience for these children and their families is important, clear recommendations for end-of-life and bereavement care, arising from the parents themselves, remain limited within current literature.”  “This report aims to describe bereaved parents’ recommendations for improvements in end-of-life care and bereavement follow-up when a child dies in intensive care.” | “Thematic analysis of incidental data from a larger grounded theory study.  Setting: Four Australian PICUs.  Subjects: Twenty-six bereaved parents participated in audio recorded, semi-structured interviews in 2015–2016. Interviews explored their experiences of having a child die in intensive care and their experiences of end-of-life care and bereavement follow-up. Data pertaining to this report were analyzed via thematic analysis.” | “Identified several areas for care delivery and improvement across 2 time periods:  During hospitalization:   - Improve communication: the need for honesty; wanted to be clearly and directly told that child’s chances of survival were limited using unambiguous language - Changes to the physical environment: provision of parents’ respite room - Better self care resources: meals and overnight accommodations - Provision of family support: community based support services as well as improved support and resources for sick child’s siblings   During dying phase:   - Private, demedicalized rooms - Familiar staff members - Support to leave the hospital: staff members to continue to stay with them after their child had died; wanted assistance to wash their child, pack their belongings and walk our of the PICU and to cars   After death:   - Provision of ongoing support from hospital or local bereavement services: follow up care; include the whole family - Improved information delivery: information about what should happen next; recommended connecting newly bereaved parents to others who have similar experiences” | “These recommendations range from simple practice changes to larger organizational modifications, offering many potential avenues for change and improvement both on an individual healthcare provider level and within individual PICU.”  “Should not advocate for standardization of end of life or bereavement follow up care, but recommendations can be provided to family to pick.” |
| Butler, A.E., 2018 | The changing nature of relationships between parents and healthcare providers when a child dies in the pediatric intensive care unit | *Qualitative* |  | 17 | “To explore bereaved parents’ interactions with healthcare providers when a child dies in a pediatric intensive care unit” | “Data were collected via semi-structured, audio-recorded interviews with 26 bereaved parents from four Australian paediatric intensive care units over 18 months in 2015–2016. Constant comparative analysis and theoretical memos were used to analyse the data.” | “The theory “Transitional togetherness” identified as the core process occurring between parents and HCPs when a child dies in PICU - demonstrates the changing nature of the parent–healthcare provider relationship across three key phases of the parents’ journey.   - Phase one, “Welcoming expertise,” focuses on the child’s medical needs, with the healthcare provider dominant in the relationship as parents prioritize the survival of the child. Parents start to learn what their role is and familiarize themselves with the environment. - Phase two, “Becoming a team,” centres around the parents’ need to recreate a parental role and work collaboratively with healthcare providers as parents realize that their child will probably die.”   ““Gradually disengaging” describes the parents’ desire for the relationship to continue after the child’s death as a source of support until no longer needed. Parents step back here and expect the HCPs to act as experts to lead them through the entire grieving process. A lack of follow up leaves parents feeling abandoned and forgotten.” | Parent HCP relationship is not static but changes over time according to changing parental needs.  Parent-HCP relationships do not end with the death of the child; this relationship only gradually diminishes over time. |
| Butler, A.E., et al., 2017 | "Some were certainly better than others" - Bereaved parents' judgements of healthcare providers in the pediatric intensive care unit: A ground theory study | *Qualitative* |  | 18 | “To explore bereaved parents’ judgements of healthcare providers, as part of a larger study examining their perceptions of the death of a child in the paediatric intensive care unit.” | “Semi-structured, audio recorded interviews were undertaken with 26 bereaved parents from 4 Australian pediatric intensive care units 6–48 months after their child’s death. Data were transcribed verbatim and analysed using open, focused and theoretical coding and the constant comparative method.” | “Bereaved parents judged healthcare providers as ‘good’ or ‘poor’ based on behaviours they exhibit. ‘Good’ behaviours were further subdivided by parents into four categories: ‘Better than others’, ‘good’, ‘very good’, and ‘fantastic’.”  Common behaviours identified as ‘good’ included:   1. Provision of practical assistance (food or toiletries; helped to orientate parents to the hospital and PICU environment) 2. Facilitation of parental presence (ensured that parents could touch and hold their child as much as possible) 3. Sharing of information (explanation of condition using non-technical terminology; patient when asked the same questions again) 4. Cared for parents (being present or providing comfort)   Behaviours for ‘very good’   1. Involved parents in direct care and demonstrated compassion   Behaviours for ‘fantastic’   1. Parents were remembered by name, demonstrated clinical competence, maintained the child’s humanity and went ‘above and beyond’   In contrast, the concept of ‘poor’ had no subdivision: all identified behaviours, including   1. diminishing parental concern 2. mishandling hope 3. adopting an unprofessional demeanour 4. judging the child’s worth,   mishandling communication | “Highest level of care seems to be personalization of care; many of the gestures identified by parents such as decorating a child’s room were small and can easily be incorporated into daily practice without significant effort.”  “Many of the behaviours considered as poor were only identified in medical staff: difficulty in communication and interpersonal skills” |
| Butler, A.E., et al., 2018 | Bereaved parents’ experiences of research participation | *Mixed* | 12.5 | 10 | “To explore bereaved parents’ experiences of research participation in a larger grounded theory study exploring experiences of the death of a child in the paediatric intensive care unit.” | “Data were obtained during follow-up phone calls made to 19 bereaved parents, five of whom provided data from their spouse, 1 week after their participation in the study. Participants were asked to reflect on their experiences of research participation, with a focus on recruitment methods, timing of research contact, and the location of their interview. Parents’ responses were analysed using descriptive content analysis.” | “Despite being emotionally difficult, parents’ overall experiences of research participation were positive. Parents preferred to be contacted initially via a letter, with an opt in approach viewed most favourably. Most commonly, participants preferred that research contact occurred within 12–24 months after their child’s death, with some suggesting contact after 6 months was also appropriate. Parents also preferred research interviews conducted in their own homes, though flexibility and parental choice was crucial.” | Not directly related to parental bereavement but more on how studies to improve bereavement process for parents should be conducted in the future |
| Butler, A.E., wt al., 2018 | Becoming a Team: The Nature of the Parent-Healthcare Provider Relationship when a Child is Dying in the Pediatric Intensive Care Unit | *Qualitative* |  | 20 | “To explore bereaved parents' perspectives of parent and staff roles in the pediatric intensive care unit when their child was dying, and their relationships with healthcare staff during this time.” | “Semi-structured interviewers were conducted with 26 bereaved parents recruited from four Australian pediatric intensive care units. The constant comparative method, coupled with open, focused, and theoretical coding were used for data analysis.” | “When the focus of care changed from ‘life-saving’ to ‘end-of-life’, parents' perspectives and desires of their and the healthcare providers' roles changed.”   - Instead of stepping back, parents begin to view their PICU role as mirroring their normal parental role - Providing comfort and support, protecting child, providing physical care and not giving up   “Parents' attempted to reconstruct their roles to match their changing perspectives, which may or may not have been successful, depending on their ability to successfully negotiate these roles with healthcare providers.”   - Wanted HCPs to focus on managing medical care to prolong life, rather than ensure survival | “HCPs need to be able to help facilitate parental roles in the PICU. Parental experiences of role loss are common in the PICU, often attributed to the equipment or the instability of the child 🡪 parental role may be regained only after appropriate support from staff.” |
| Cacciatore, J., et al., 2019 | The Long Road to Farewell: The Needs of Families With Dying Children | *Qualitative* |  | 12 | “Families of dying children are profoundly impacted by numerous interactions with health-care providers before, during, and after their child’s death. However, there is a dearth of research on these families’ direct, qualitative experiences with health-care providers. This study presents findings from interviews with 18 family members, predominantly parents, regarding their experiences with health-care providers during a child’s terminal illness, from diagnosis to death.” | “A qualitative descriptive methodology with phenomenological overtones informed the present study.”  “In a series of open-ended interviews, parents were encouraged to describe as much as they wanted of their experience during these times. Minimal direction was supplied.”  “Data were then transcribed verbatim and inductively analyzed employing conventional content analysis techniques (Hsieh & Shannon, 2005) to topically group statements as pertinent themes emerged.” | “The importance of compassion emerged as a salient theme, manifested in myriad ways, and connected to participants’ perception of caregiver presence in multiple domains.”  “Families were likewise negatively affected by a wide variety of situations and behaviors that represented individual or institutional abandonment or nonpresence, and thus compounded the experience of loss.” | “HCPs strongly influence families’ experiences during a child’s illness, dying, and death. The expression of compassion via warmth, shared decision making, open communication, flexibility, effective care, and a host of other manifestations were key factors that either aided or hindered families’ ability to cope with their child’s illness and death.”   - HCPs need to address parents’ emotional needs - Manner and circumstances in which bad news is shared with parents are remembered – nonpresence and abandonment are felt in these moments - Idea of double abandonment: esp in the scenario of pain control |
| Caeymaex, L. et al, 2012 | Perceived role in end-of-life decision making in the NICU affects long-term parental grief response | *Mixed methods* | 9 | 11 | “Background Shared decision making (DM) is increasingly advocated as the most appropriate model to support parents confronted with end-of-life (EoL) decisions for a child in the neonatal intensive care unit (NICU). However, few studies have explored its impact on their long-term grief.  Objectives The aim of this study was to investigate whether parental perception of the type of involvement in the EoL decision-making process (EoL DMP) for their child in the NICU is related to their long-term grief outcome” | “A retrospective study with mixed methods. The study included parents whose child died from 2002 through 2005 in one of four NICUs in different areas in France, with interviews of 78 individual parents of 53 children, 2.7±0.6 years after the child’s death. Parental perception of the type of involvement in the EoL DMP was determined by qualitative analysis of face-to-face interviews and classiﬁed as follows: shared, medical, informed parental and no decision. Grief reactions were assessed with the Texas Revised Inventory of Grief  (TRIG-F).”  In this paper, we present the data from the face-to-face interviews  because only this method allowed an accurate assessment of the  parents’ self-perceived role in the DMP through an indepth  investigation of the different steps and feelings of the EoL deci-  sion. | “Current grief scores differed signiﬁcantly according to the perceived type of EoL DM. Shared DM was associated with lower TRIG-F scores (less grief) than were the other types of EoL DM (F=7.95; p=0.05). The baby’s perceived suffering was also associated with higher grief scores (F=6.51, p=0.01).” | “In decisions to forego life-sustaining treatment in the NICU, the perception of a shared decision is associated in the long term with lower grief scores than perceptions of the other types of DM.” |
| Cai, S., et al., 2020 | Spiritual needs and communicating about death in nonreligious theistic families in pediatric palliative care: A qualitative study | *Qualitative* |  | 23 | “Spiritual support should be offered to all patients and their families regardless of their affiliated status with an organized religion.  Aim: To understand nonreligious theistic parents’ spirituality and to explore how parents discuss death with their terminally ill children in mainland China.” | Design: Qualitative study.  “Setting/participants: This study was conducted in the hematology oncology center at Beijing Children’s Hospital. Participants in this study included 16 bereaved parents.” | “Participants described themselves as nonreligious but showed a tendency toward a particular religion. Parents sought religious support in the face of the life-threatening conditions that affected their child and regarded the religious belief as an important way to get psychological and spiritual comfort after experiencing the death of their child.” | “Religious support could partially address parents’ spiritual needs. Parents’ spiritual needs still require other supports such as bereavement services, death education, and family support groups.”  “For patients who come from nonreligious theistic families, their understanding of death was more complex and may be related to atheism.”  “Religious support could be an element of spiritual support for nonreligious theistic parents of terminally ill children.”  “Multiple strategies including religious supports and nonreligious supports should be rationally integrated into spiritual support of nonreligious theistic family. Patient’s personal belief in death should be assessed before discussing death with them.” |
| Caicedo, C., et al., 2019 | Parents' Wishes for What They Had or Had Not Done and Their Coping After Their Infant's or Child's Neonatal Intensive Care Unit/Pediatric Intensive Care Unit/Emergency Department Death | *Qualitative* |  | 14 | “More than 40,000 infants and children die in the US annually, most in NICUs and PICUs. Around the time of the infant or child’s death, parents are faced with making stressful decisions on treatments, resuscitation, and withdrawal of life support.”  “This qualitative study asked 70 mothers and 26 fathers 3 open ended questions on what they wish they had, had not done, and coping 2, 4, 6, 13 months after their infant’s/child’s neonatal intensive care unit/pediatric intensive care unit/emergency department death.” | “This descriptive study was part of a larger longitudinal study (NIH, R01 NR012675) examining surviving siblings’ and parents’ health and functioning 2–13 months after a child’s NICU/PICU death23. In this study and in our previous study (NIH, R01 NR009120)22, we used the same 3 open ended questions on what parents wished they had or had not done and their coping around the child’s death. Data in this study were collected at 2, 4, 6, and 13 months post-infant’s/child’s death.” | “Wish you had done - Wishing to have spent more time with the child (24%), having chosen a different treatment path (19%), advocated for care changes (17%) and allowed the child his/her wishes (10%) were the most frequent of the total responses from mothers.Wishing to have spent more time with the child (43%), gotten care earlier (24%), having chosen a different hospital or care site (10%) and having refused treatment or gotten a second opinion were the most frequent of the total responses from fathers.”  “Wish you had not done.—For mothers, wishing she had not agreed to child’s surgery/treatment (33%), not taken her own actions (indicating self-blame 29%), not left the hospital before the child died (13%), and not make the child do things he/she didn’t want to do or punished them (9%) were the most frequent of the total responses. For fathers, wishing he had not been so hard on the child (39%), not agreed with doctors and treatment (28%), and wishing he had not done some actions (indicating self-blame 22%) were the most frequent of the total responses.”  “Coping with the child’s death- Religious activities (20%), caring for self (16%), and talking about and with the deceased child (14%) were the most frequent of the mothers’ total responses. Fathers’ responses included caring for self (29%), religious activities (21%), remembering and honoring the child (14%), spending time with family and friends (13%) and avoiding talking or thinking about the deceased child (13%) as the most frequent of the total responses.” | “Both mothers and fathers wished they had spent more time with their child and had not agreed to surgery/treatments. Most frequent coping was caring for themselves, likely to care for the family and retain employment. Nurses must be sensitive to parents’ need for time with their infant/child before and after death and to receive information on child’s treatments at levels and in languages they understand.” |
| Caprice A. Knapp,et al, 2009 | Family Support Services in Pediatric Palliative Care | *Non-empirical* |  |  | “A fundamental premise of pediatric palliative care is that support is provided not only to the ill child but to the family as well. In doing so, a number of services may be offered to family members throughout the child’s illness, at the time of death and into bereavement, such as respite, counseling, expressive therapies, and bereavement support. Support may also be needed for the child’s peers at school, church, or on sporting teams. Evidence on family supportive care in pediatric palliative care research is scarce.” | “This article focuses  on the existing expert recommendations and research findings about family-support services” | “Support services at Diagnosis:  There is a role for supportive care at the time of diagnosis, but what is unclear is how pediatric palliative care providers can provide such care. stage. A well-documented barrier to pediatric palliative care is the fact that families may view this service as synonymous with giving up hope.  Support Services Throughout Children’s Illnesses:  Coordinating care can reduce stress for the parents and help them navigate the medical system. Many other innovative services exist outside of the medical system for families whose children have life-limiting illnesses. One example, Donna’s Dream House in Blackpool, England, allows families of children with life-limiting illnesses to enjoy a seaside vacation free of charge.  Support Services at the End of Life:  Admittedly, it is difficult to conduct research at the end of a child’s life due to patient and parent burden and there continues to be debate around the ethical considerations of approaching parents to participate in research at the end of life and even after their children die.End-of-life studies have found that parents desire information that is honest, clear, and accurate about what to expect as death approaches; they also want privacy, emotional support, and options at the end of life such as being able to hold their child as death approaches. Family members emphasize how critical their interactions with medical staff are at the end of life.  Bereavement Support:  Although some grief responses are relatively universal, the experience is highly individualized. In evaluating what services might be appropriate; providers must consider personality traits, gender differences, previous experiences with loss, culture, ethnicity, and spiritual beliefs among other factors.” | “Pediatric palliative care strives to treat the family as a unit; providing much needed support to parents, siblings, extended family members, and the community. However, if pediatric palliative care is to be integrated with curative care, much more work needs to be done regarding the needs of families at the point of diagnosis and during times of decreased health status when support may be greatly valued.” |
| Clancy, S., & B, Lord., 2018 | Making Meaning After the Death of a Child | *Non empirical* |  |  | To share their stories with medical providers and other parents who had experienced the death of a child | Anecdotal.  Two bereaved parents articulate how they made meaning after the deaths of their children. | Common themes found in the anecdotes:   - Good support from medical staff  1. Psychologist serving as grief counsellor to help process parents’ anticipatory grief following the diagnosis and helped to put the pieces together so that child’s death was not a tragedy to their family 2. Did not like it when the care from various specialists were fragmented 3. Were recognized as equal participant in child’s medical care: listened to observations, questions and concerns and give honest answers even if they may not accord with medical team’s consensus 4. Knew the medical staff as other people who have also experienced the loss of a parent or sibling  - Contributed back to society in the name of their children  1. Started a small foundation 2. Shared their story to medical students and residents (delivering diagnoses, giving anticipatory guidance, helping to identify care goals and values, and accompanying family through child’s transition to end of life; and how palliative care made a very big difference)   Help other parents to their story; share with other bereaved parents | Pediatric palliative care can be conceived as a way to provide space for patients and parents to tell their stories outside of the specifics of illness, thereby initiating the process of meaning making.  “Viewing video interviews of parents of children with serious illnesses allows medical trainees to understand the parent perspective and hear their stories, enhancing trainees’ communications skills, empathy and compassion.” |
| Coelho, A., et al., 2018 | Caregiver anticipatory grief: phenomenology, assessment and clinical interventions | *Descriptive (Review)* |  |  | “To synthesize recent findings on anticipatory grief in caregivers, referring to its phenomenology, assessment and clinical interventions.” | “Literature review of papers published in the last 2 years.” | Pertaining to parents as caregivers:   - Feelings of helplessness and impotence related to the multiple losses and frequent failures in their child’s treatment - Perspective of finitude led parents to reconfigure their meaning in life: they enhance the parent– child relationship, share decision-making and give the child more autonomy   In other cases, parents take more control and responsibility for the deci- sions about their child’s life and death. | “Difficult circumstances, such as caregiver exhaustion and lack of preparation to death, also contribute to high levels of anticipatory grief.”  “Support of caregiving skills and self-help strategies, providing adequate information about illness progression, validation of grief feelings, reframing roles, anticipation of future losses and relationship reformulation can be helpful techniques specifically addressed to caregiver anticipatory grief.” |
| Cortezzo, D.E. et al, 2014 | End-of-Life Care in the Neonatal Intensive Care  Unit: Experiences of Staff and Parents | *Quantitative* | 9.5 |  | “The aim of the study is to determine the perceptions of end-of-life care practices and experience with infants who have died in the NICU among neonatologists, advanced practitioners, nurses, and parents, and also to determine perceived areas for improvement and the perceived value of a palliative care team.” | “This descriptive, exploratory cross-sectional study using surveys consisting of 7-point Likert scales and free response comments was sent to all neo-natologists (n ¼ 14), advanced practitioners (n ¼ 40), and nurses (n ¼ 184) at Connecticut Children’s Medical Center’s neonatal intensive care units (NICUs) in April 2013 and to all parents whose infants died in these NICUs from July 1, 2011, to December 31, 2012 (n ¼ 28).” | “The response rates were 64.3% for physicians; 50.0% for practitioners; 40.8%for nurses; and 30.4% for parents. Most providers reported they feel comfortable delivering end-of-life care. Bereavement support, debrieﬁng/closure conferences, and education did not occur routinely. Families stressed the importance of memory making and bereavement/follow-up. Consistent themes of free responses include modalities for improving end-of-life care, inconsistency of care delivery among providers, and the importance of memory making and follow-up.” | “End-of-life experiences in the NICU were perceived as variable and end-of-life practices were, at times, perceived as inconsistent among providers. There are areas for improvement, and participants reported that a formalized palliative care team could help. Families desire memory making, follow-up, and bereavement support.” |
| Côté-Arsenault,D., et al., 2019 | African American and Latino bereaved parent health outcomes after receiving perinatal palliative care: A comparative mixed methods case study | *Mixed methods* | 11.5 | 12 | “Death of one's infant is devastating to parents, negatively impacting couple relationships and their own health. The impact of a prenatally diagnosed life-limiting fetal condition (LLFC) on parents of minority status is unclear.”  “To examine the person characteristics, quality of perinatal palliative care (PPC) received and parent health outcomes.” | Mixed methods case study  “Bereaved couples, 11 mothers and 3 fathers of minority or mixed races (11 African American and Latino, 1 White Latino and 2 White parents) completed the survey; 7 were interviewed. Inclusion of up to three years since the loss.” | - Parents rated their general health close to good, physical health close to normal but mental health lower than the population norm. Clinical caseness (abnormal levels) of anxiety were reported in 50% of parents whereas depression scores were normal. The experience of fetal diagnosis and infant death had a negative impact on the health of 40% of participants however, parents could not identify what specifically caused their health problems. - Most were satisfied with their PPC but some shared that original providers were not supportive of pregnancy continuation. - After the baby's death, 71% reported closer/stronger couple relationships.   “Once parents found PPC, their baby was treated as a person, they spent time with their baby after birth, and found ways to make meaning through continuing bonds – babies were treated as a person and not a diagnosis; now have momentoes, and have found ways to honour their babies.” | Number of PPC programs have grown exponentially in the past decade – parents find PPC to be accepting, helpful and to have greatly improved their experience. |
| Cox, S.A. (2004) | Pediatric bereavement: supporting the family and each other | *Descriptive (Review)* |  |  | “Childhood deaths are relatively uncommon but very stressful to both family members and health care providers. Bereavement programmes are helpful in guiding and supporting family into and through the bereavement process. Cox describes the current literature on bereavement practices, outlining the components of a bereavement programme and current challenges for nurses.” | “Review of the literature which describes bereavement programmes in the Paediatric Bereavement Program at Children’s Hospital and Health Center in San Diego, California, and other programmes. “ | Elements of a comprehensive Paediatric Bereavement programme:   1. Team Approach – multi-disciplinary care team 2. Recognition of cultural differences: wrt death and dying 3. Integration of family into the care of the dying: bedside privacy, parents to hold the child, involving family members in aftercare 4. Memory packets or boxes 5. Facilitated support groups 6. Resource lists and information 7. A remembrance ceremony 8. Continued contact with family members 9. Staff education and development 10. Programme evaluation and feedback   Challenges for nurses:   1. Increasing self awareness: non verbal cues could serve as additional stressors to families 2. Balancing composure with care 3. Finding time to learn new concepts and strategies 4. Mentoring the next generation of caregivers 5. Participating in changing the bereavement culture in your organization 6. Maintaining a bereavement programme in an era of shrinking financial resources | “The greatest obstacles to optimal paediatric bereavement and psychosocial care are personally held convictions and biases on the part of the health care team members.” |
| Currie, E. R. et al, 2016 | Parent Perspectives of Neonatal Intensive Care at the End-of-Life | *Qualitative* |  | 19 | “This descriptive qualitative study explored parent experiences related to their infant's neonatal intensive care unit (NICU) hospitalization, end-of-life care, and palliative care consultation.” | “A descriptive qualitative design aims to present an inclusive summary of the phenomena in question in everyday language (Sandelowski, 2000, 2010) and was used to explore and describe parent experiences related to their infant's NICU hospitalization, EOL care, and PPC consultation. In-depth, face-to-face inter-views occurred up to four years after infant death in the NICU.”  Theoretical Framework  The Dual Process Model of Coping with Bereavement was used as the theoretical framework for this study.. | ““Life and death in the NICU environment" emerged as the primary theme with the following categories: ups and downs of parenting in the NICU, decision-making challenges in the NICU, and parent support. Parents encountered challenges with areas for improvement for end-of-life and palliative care in the NICU.” | “The findings from this study identified the importance of exploring parents' experiences during their infant's care near the end-of-life in the NICU. The experiences of these parents living with decisions surrounding infant death in the NICU in this study provide support for the oscillating process described in the Dual Process Model (Stroebe & Schut, 1999). Being a parent in the NICU was extremely important for these participants regardless of the infant's prognosis. NICU nurses played a critical role in facilitating parenting in the NICU by teaching the parents how to interact with their critically ill infant and remaining a source of support throughout the NICU hospitalization.” |
| Currie, E. R., et al., 2019 | Life after loss: Parent bereavement and coping experiences after infant death in the neonatal intensive care unit. | *Qualitative* |  | 20 | “The dual process model of coping with bereavement was used as a conceptual framework to help understand how parents cope with grief after infant death.”  “Framework - A central component to this model is the concept of oscillation between loss-oriented and restoration-oriented stressors in a dynamic process of confrontation and avoidance, in order to adapt successfully.” | “A qualitative descriptive approach was used to explore and describe parents’ bereavement coping experiences following a NICU hospitalization where neonatal palliative and end of life were provided.” | “The two major themes identified from the data were (a) living with loss and (b) coping with grief over time.  (a) Living with loss - While many of the parents were able to function and continue with their daily lives, they described grief as “always being there”. The theme is comprised of five subthemes—bereavement and grief over time, mental health changes, spiritual suffering, personal growth after loss, and life changes after loss—which serve to provide depth to the primary theme.  (b) Coping with grief over time - Barriers and facilitators to coping ranged from loss-oriented stressors such as confronting multiple losses simultaneously or difficulty reminiscing through the infant’s personal items to restoration-stressors such as taking on new projects and roles after infant death.” | “Living with infant death was a process that resulted in major life changes and a process of oscillating among various coping strategies.” |
| Dawn M. Hawthorne et al, 2016 | Parent Spirituality, Grief, and Mental Health at 1 and 3 Months  After Their Infant's/Child's Death in an Intensive Care Unit | *Quantitative* | 13 |  | “Problem—The death of an infant/child is one of the most devastating experiences for parents and  immediately throws them into crisis. Research on the use of spiritual/religious coping strategies is limited, especially with Black and Hispanic parents after a neonatal (NICU) or pediatric intensive  care unit (PICU) death.  Purpose—The purpose of this longitudinal study was to test the relationships between spiritual/ religious coping strategies and grief, mental health (depression and post-traumatic stress disorder) and personal growth for mothers and fathers at 1 (T1) and 3 (T2) months after the infant's/child's death in the NICU/ PICU, with and without control for race/ethnicity and religion.” | “An interview was conducted with 165 bereaved parents of 124 deceased infants/ children. Grief was measured with four of the six subscales in the Hogan Grief Reaction Checklist. Each of the 61 items were rated on a 5 point scale. Depression measured with the Beck Depression Inventory. PTSD measured with the Impact of Events Scale-Revised. Spiritual coping measured with SCS.” | “Bereaved parents' greater use of spiritual activities was associated with lower symptoms of grief, mental health (depression and post-traumatic stress), but not post-traumatic stress in fathers. Use of religious activities was significantly related to greater personal growth for mothers, but not fathers. Use of spiritual activities was more strongly related to all outcomes for mothers and fathers than use of religious activities. Bereaved mothers' greater use of spiritual activities, but not religious activities, was significantly related to lower symptoms of grief (despair, detachment and disorganization), depression, and PTSD at T1 and T2.” | “Spiritual strategies and activities helped parents cope with their grief and helped bereaved mothers maintain their mental health and experience personal growth. Spiritual activities may assist bereaved mothers to reduce their symptoms of grief, depression, PTSD and increase personal growth over a longer period of time than religious activities.  Gender differences in coping with grief are supported in the literature.” |
| Decinque N., et al. , 2004 | Bereavement support for families following the death of a child from cancer: practice charactertistics of Australian and New Zealand pediatric oncology units | *Quantitative* | 9 |  | “Hospital-based bereavement support programs in the paediatric setting are a relatively recent development and reflect both an acknowledgment by health professionals of the complexity of parental grief, as well as the role they have in the provision of support for bereaved families.  The author seeks to explore the experiences and needs of nine parents who had received hospital-based bereavement support following the death of their child from cancer, in Western Australia.” | “This is a descriptive study, involving surveys of 10 pediatric oncology units in Australia and New Zealand. Survey instrument consisted of a 19-item questionnaire with open ended and closed questions.” | “Six prominent themes emerged from thematic data analysis: personal grief, personal coping, concern for siblings of the deceased child, hospital bereavement support, community supports and unmet needs. Parents identified the need for more supportive contact from hospital staff during the palliative phase and following the child’s death, early provision of information on how to practically and emotionally prepare for the death of their child, contact with other bereaved parents, and formal grief support for siblings.” | “Areas for future research include exploration of parents’ wish to become involved in activities to help others, bereavement support for siblings, the level of contact with the hospital unit that may be therapeutically beneficial, and parental behaviors associated with accessing both hospital and community-based bereavement supports.” |
| Decinque N., et al. , 2006 | Bereavement Support for Families Following the Death of a Child from Cancer | *Qualitative* |  | 19 | “Hospital-based bereavement support programs in the paediatric setting are a relatively recent development and reflect both an acknowledgment by health professionals of the complexity of parental grief, as well as the role they have in the provision of support for bereaved families.” | “This study explored the experiences and needs of nine parents who had received hospital-based bereavement support following the death of their child from cancer, in Western Australia. Narrative inquiry was adopted as the methodological approach for this study.” | “Six prominent themes emerged from thematic data analysis: personal grief, personal coping, concern for siblings of the deceased child, hospital bereavement support, community supports and unmet needs.  Parents identified the need for more supportive contact from hospital staff during the palliative phase and following the child’s death, early provision of information on how to practically and emotionally prepare for the death of their child, contact with other bereaved parents, and formal grief support for siblings.” | “Areas for future research include exploration of parents’ wish to become involved in activities to help others, bereavement support for siblings, the level of contact with the hospital unit that may be therapeutically beneficial, and parental behaviors associated with accessing both hospital and community-based bereavement supports.” |
| deJong-Berg, M.A., Kane, L. , 2006 | Bereavement care for families part 2: evaluation of a pediatric follow-up programme | *Mixed methods* | 8.5 | 18 | “In North America, parents are not expected to outlive their child.  When they do, neighbours, co-workers, friends and family do not know what to say or do resulting in parents feeling isolated in their grief and left alone to find the support they need. The Paediatric Palliative Care team at Capital Health's, Stollery Children's Hospital (Stollery) in Edmonton, Alberta, Canada began a programme of bereavement follow-ups in April 2002 to offer support to families who had experienced the death of their child.” | “In Part 1 of this article (deJong-Berg and deVlamming, 2005) implementation and the current state of the programme is outlined, and in Part 2 we present the results of the programme evaluation conducted in Spring, 2005. Eighty-one families were invited to take part in a satisfaction survey to determine the helpfulness of this bereavement follow-up programme. Twenty-nine parents, representing 21 families, took part.”  The questions addressed in this programme evaluation were:   - Is the programme goal being met? - Are parents satisfied with the programme as it is currently being delivered? - Do parents have suggestions for improvements to the programme?" | “Parents reported that written information received was useful and that they felt supported knowing a resource was available.  They also felt also felt that the programme extended the care given by the Stollery staff throughout their child's illness and death.”  Answers to the questionnaire (themes)   - Mementos offered at time of child's death - Letters and accompanying materials - Calls - Memorial Service - Groups - Sources of support to families - Comments on the programme and suggestions for improvement | “This evaluation study provided information in the five main areas of the standard practice of bereavement follow-up at the Stollery Children's Hospital including memory creation, letters and cards, information and pamphlets, phone calls, and the memorial service. It did not offer information on the individual counseling and offered only limited information on group work as theses are not standard features of the programme because of the distances some families live from the Stollery.” |
| Denhup, C., 2019 | Bereavement care to minimize bereaved parents’ suffering in their lifelong journey towards healing | *Qualitative* |  | 17 | To describe the lived experience of parental bereavement. | “Interpretive phenomenological study based in United States. Six parents, each of whom experienced the death of a child due to cancer at least one year prior, participated in conversational interviews to share what it has been like for them since their child's death.”  Thematic analysis done of the data. | Themes:   1. Profound suffering: suffering is experienced throughout parental bereavement, regardless of the amount of time that passes after a child's death. Evidence of this is found in the fact that all participating parents experienced suffering of similar nature. Their suffering was found to be incomparable, indescribable, and undeserved. 2. Things that people say or do cause additional suffering: a) when people manage or explain b) when people avoid c) when people question d) when people complain and e) when people compare 3. Things that people say or do to minimize additional suffering 4. Healthcare providers and care processes cause additional suffering: poor communicati0on, insufficient information, exclusion from child’s care, and differences in care goal 5. Healthcare providers and care processes minimize additional suffering: caring behaviours, partnering in decision making, maintaining presence, and acts of kindness   Fostering support: support not just immediately after a child’s death, but throughout their lifetime. Many parents felt that the support waned thereafter, even though they continued to be in need of support. Needed empathetic reassurance. Also expressed a need for support as they engaged in self-care behaviours. Also shared the examples of helpful support group activities that could be incorporated into either professional-led or peer support groups. | Recommendation from paper: Since bereaved parents' journey is lifelong, nurses must consider their role in supporting parents for the long-term.  Role for even adult health nurses to identify adult patients who are recently bereaved, and ensure that supports are in place not just for the current minimum 13 months post loss but throughout bereaved parent’s lifetime. |
| Dias, N., et al., 2018 | Bereaved Parents’ Health Status During the First 6 Months After their Child’s Death | *Quantitative* | 13 |  | “Bereaved parents have higher mortality and morbidity rates when compared to nonbereaved parents. Acute illnesses, hospitalizations, and medication changes are highest in the first 6 months. An understanding of bereaved parents’ health risk indicators can help inform development of health promotion and disease prevention measures.”  “To examine bereaved parents’ physical, mental, and social health during the first 6 months after their child’s (<12 years) death from a life-threatening illness.” | “A prospective descriptive study examined 8 parent dyads. Parents completed health surveys (Patient-Reported Outcomes Measurement Information System–global, social, and sleep; Brief Symptom Inventory [BSI] 18), which are used to assess parents’ health at 3 and 6 months after their child’s death (children below 12 years). Demographic data included a medical history, hospital or emergency department visits, and smoking and alcohol intake. Descriptive statistics were used to compare parents’ scores to US general population scores.” | - Mothers’ and fathers’ physical, mental, and sleep health scores were typically within 1 to 2 standard deviations of the population norms. However, their social health scores were as low as 3 standard deviations and all parents’ scores were below population norms. - Self-Health Perception Scale: Most parents rated their overall health at 7 or 8 at both time points of 3 months and 6 months. While 43.75% of the parents had an improved score at 6 months, the scores of more fathers than mothers were reduced; and more mothers than fathers had stable scores at both time points - Comparisons of 3 and 6 months’ health scores identified that mothers had trends toward worsening in their physical and social health, also indicating a need for further examination of the relationship of social health to parents’ physical health. - Comparison between mothers’ and fathers’ health concurred with prior studies, in which fathers fared better than mothers at 6 months (but not statistically significant due to small sample size)   Out of 16 participants, 4 participants had new diagnoses within the first 6 months 🡪 bereaved parents health is at risk | “Bedtime was the most difficult time for bereaved parents and that parents’ social interactions with family, friends, and community in general has negatively affected.”  “Potential lack of sleep and decline in the quality of social relationships may be precursors that affect other health outcomes.” |
| Drew, D., et al., 2005 | Parental grieving after a child dies from cancer: is stress from a stem cell transplant a factor? | *Quantitative* | 13 |  | “Drew seeks to investigate psychological distress, family functioning and complicated grieving in parents whose child had died from cancer, and as a function of whether (a) the deceased child had also received stem cell therapy at any time during curative treatment and (b) the place of the child’s death (home or hospital).” | Cross sectional case-match design.  “56 Australian bereaved parents in two groups: 28 whose child had also received SCT, matched with 28 (on deceased patient variables) whose children had not received SCT.”  Measures used:   1. Depression, Stress and Anxiety scale (DASS) 2. Core Bereavement Inventory (CBI) 3. Inventory of Complicated Grief (ICG)   Family Assessment Device – General functioning scale | “Parents in the SCT groups reported relatively higher levels of depression, anxiety and stress. For those whose child had died in hospital, a greater likelihood of meeting criteria for traumatic grief than those parents who deceased child had not received SCT.” | “Routine psychosocial screening, especially for families undergoing SCT may contribute usefully to a proactive model of palliative care in identifying parents at risk for complicated bereavement outcomes.” |
| Dutta, O., et al., 2019 | Lived experience of a child’s chronic illness and death: A qualitative systematic review of the parental bereavement trajectory | *Descriptive (Review)* |  |  | “The present authors believe that a consolidated investigation, which summarizes findings from various studies, would provide a stronger foundation to understand the experience of parental bereavement due to chronic illness and death of one’s child, the typical challenges and the potential for growth following this trauma.” | “To understand the lived experience of parents who have lost their child to a chronic life limiting condition, six major databases were searched by adhering to the Preferred Reporting Items for Systematic Review and Meta-Analyses guidelines. Articles were screened for appropriateness using the Sample, Phenomenon of Interest, Design, Evaluation, Research type tool, and relevant qualitative studies were selected for full-text data analysis using Thematic Synthesis.” | “Findings were categorized into 13 themes that were further organized into a four-phase trajectory of parental bereavement experience of child loss, namely: Liminal Margin, Holding Space, Navigating Losses, and Reconstructing Lives” | “The reviewed literature places considerable emphasis on (1) continuing bonds with a deceased child, (2) constructing a renewed sense of meaning following the loss, (3) social isolation following the death of a child, (4) feelings of regret towards neglected healthy children, and (5) relationships with medical professionals, which can be noted from the content density of the themes.” |
| Dutta, O., et al., 2020 | Trauma to transformation: the lived experience of bereaved parents of children with chronic life-threatening illnesses in Singapore | *Qualitative* |  | 21 | “Chronic illnesses are prevalent causes of child mortality around the world. Despite growing research that examines the lived experience of parents bereaved by their child’s chronic life-threatening illness, there is no such study within the Asian context.” | “Meaning-oriented, strength-focused interviews were conducted with 25 parental units (i.e. 6 couples, 13 lone mothers, 4 lone fathers, and 2 primary parental figures) who lost their child to chronic life-threatening illness in Singapore (N = 31), including those of Chinese (n = 17), Malay (n = 10) and Indian ethnicities (n = 4), between August 2017 and April 2018.” | “7 themes and 25 sub-themes that were organized into a Trauma-to-Transformation Model of Parental Bereavement. Model shows the major milestones in participants’ lived experience of their child’s chronic life-threatening illness and death”:   1. Diagnosis of illness and emotional turmoil 2. Mourning of child’s death and losses which accompanied the death 3. Participants’ experiences of posttraumatic growth through reflection of their journey of caregiving and child loss 4. Behaviors or rituals that helped participants to regain power over their lives 5. Sustaining an intime bond with child beyond death 6. Transcend their loss by deriving positive outcomes from their experience  - Experiences and well-being embedded within the health and social care ecosystem, and in turn impacted by it | “Asian parents in this study de- scribed their disenfranchised grief following the loss of their child whereby family members did not adequately acknowledge their grief and the intensity of their suffering.”  “Grief counselling with Asian populations must emphasize familial and social connections, which play a critical role in providing support in end-of- life care.”  “Could also be useful for clinicians to draw upon novel psycho-socio-spiritual interventions such as Family Dignity Intervention (FDI) which aim to enhance palliative care in the Asian context.” |
| Elizabeth Kacel, B.A., et al, 2011 | Understanding Bereavement: What Every Oncology Practitioner Should Know | *Descriptive (Review)* |  |  | “Bereavement deserves special attention in oncology practices because of the frequency with  which providers, patients, and their families encounter death. The biological, psychological,  and socio-cultural demands of a potentially terminal illness such as cancer put all involved parties at risk for experiencing grief at many points throughout the continuum of care, both  before and after death.  A vast body of research has examined grief in terms of risk factors, clinical presentations,  and treatment options. In this literature, many terms have been employed to discuss these aspects of the experience of loss.” | Review paper. | Clinical presentations of grief:   - Anticipatory grief - Grief surrounding time of death - Maladaptive Responses to Bereavement - Prolonged Grief Disorder   Interventions and treatment for grief:   - Clinical support from hospital staff - Psychodynamic and Interpersonal Treatments - Cognitive Behavioral Therapy - Group-oriented Therapeutic Approaches - Family Therapy - Internet-based Therapy - Combined psychopharmacological treatments - Treatments for Prolonged Grief Disorder   Hospice care | “Many of the theories of grief contain overlapping elements.These elements may be considered and integrated from within a biopsychosocial framework which takes into account the biological, psychological, and socio-cultural ramifications of losing a loved one.  Many treatment models have been proposed for grief, and although the literature still suggests mixed results, further research into specific sub-groups of bereaved individuals may be helpful in developing reliable and valid targeted interventions.  Overall the literature indicates that the role of oncology staff in helping families cope with grief is immeasurable. Medically and psychologically, clinicians are well positioned to reach out to patients and prevent post-loss suffering.” |
| Eva Bergstraesser, 2012 | Pediatric palliative care—when quality of life becomes the main focus of treatment | *Descriptive (Review)* |  |  | “Despite great advances in medicine, children suffering from life-limiting illnesses and their families may still be confronted with the unnatural fact of a shortened lifespan. Pediatric palliative care (PPC), a relatively young discipline, focuses on this patient group and strives to ease suffering and enhance quality of life. Many readers may think that this is an inherent part of medicine and does not need speciali-zation. I agree to some point, however, there might be a specific role for specialized physicians in PPC, as in other specialties. This review article aims to provide an overview of current pediatric palliative care.” | Narrative Review | “Pediatric palliative care (PPC) focuses on children and adolescents with life-limiting diseases. It may be initiated at various points of the disease trajectory, if possible early enough to support living with the best possible quality of life despite a limited lifespan. From birth to adolescence, children with a broad spectrum of diseases may benefit from PPC. Since 50% of deaths in childhood occur within the first year of life, PPC is just as relevant to neonatology. Causes of death in the neonate and young infant are due to perinatal conditions such as preterm birth and congenital disorders and syndromes; in older children, external causes, such as traumatic injuries, outweigh disease-related causes of death. PPC may last from a few hours or days for neonates to many years for children with complex chronic conditions. For neonates, PPC often has the character of end-of-life (EOL) care followed by bereave-ment care for the family. For older children, PPC can clearly be differentiated from EOL care; its indications include progress or deterioration of disease, marked instability of the child’s condition, increase in the need for technical or medical support, increase in suffering, or failure of treatment. If a child’s need for palliative care is established, useless and potentially harmful treatments may be withheld and informed choices can be made about treatment, care, and the remaining life of the child.” | “This review aims to provide knowledge for clinicians who care for children and adolescents at risk of dying from their disease. PPC can improve the child’s remaining lifetime by focusing on quality of life and goals that are defined by the child and his or her family.” |
| Falkenburg, J.L., et al. (2018) | The Importance of Parental Connectedness and Relationships with Healthcare Professionals in End-of-Life Care in the PICU | *Qualitative* |  | 25 | “Support from healthcare professionals in a PICU is highly valuable for parents of dying children. The way they care for the patients and their families affects the parents’ initial mourning process. This study explores what interaction with hospital staff is meaningful to parents in existential distress when their child is dying in the PICU.” | “Qualitative interview study on 36 parents of 20 children who had died in PICU (Erasmus University Medical Center – Sophia Children’s Hospital, Rotterdam and the Netherlands) 5 years previously.”  “Parents participated in audio-recorded interviews in their own homes. Interviews were transcribed and analyzed using qualitative methods.” | “Parents’ narratives of their child’s EOL stage in the PICU were experiences of estrangement, emotional distancing and loneliness.”  “Significant moments shared with hospital staff that remained valuable even after 5 years”:   1. Personal connectedness, reflected in frequent informational updates  - Parents of children with long admission said that they sometimes felt that they were forgotten because their child’s medical situation remained unchanged and thus apparently did not warrant any communication  1. Personal commitment of professionals  - Nonverbal acts and gestures: doctor not showing up when parents had asked for him/ nurses who said that they would come to the funeral but did not show up were painfully remembered  1. Interpersonal contact with doctors and nurses  - Had helped them to have normal conversations with the HCPs   Personal interaction also reflected in the sharing of motions, like crying together | “Importance of relationship-centered care. Interpersonal sensitivity, and experiencing a bond with staff members conveying emotions and concern help families in bereavement process.”  “Everyday conversations are important for both professionals and families in ICUs 🡪 make it easier to communicate in more complicated situations such as bringing of bad news”  Recommendations:   1. Make contact with parents in all interactions 2. Offer parents opportunity to speak about their fears and hopes   Practical training to help create awareness of parental existential needs and perspectives. |
| Falkenburg, J.L., et al. (2020) | The fragile spirituality of parents whose children died in the pediatric intensive care unit | *Qualitative* |  | 20 | “Spiritual care is recognized as a relevant dimension of health care. In the context of pediatric palliative end-of-life care, spirituality entails more than adhering to a spiritual worldview or religion.” | “Qualitative interview study with 36 parents of 20 children who had died some 5 years earlier in the 34-bed multidisciplinary tertiary care PICU of the Erasmus MC-Sophia Children’s Hospital, Rotterdam, the Netherlands. Children’s age at the time of death varied from 2 weeks to 14 years.” | Themes raised:   1. Parental interpretations of the child’s condition in the PICU 2. Spiritual features of connectedness: transcending ordinary events 3. Receptiveness 4. Ambivalence 5. Vision on death 6. Affinity to spirituality and faith | 1. “People who do not necessarily adhere to or seek for a spiritual worldview – let alone use terms like ‘spiritual’ to describe their experiences – may still foster a sense of spirituality” 2. “There is also a vagueness surrounding the interpretation of events. The fragile spirituality we discovered does not depend on (prior) spiritual or religious affinity; the transcending meaning of lasting connectedness suffices to parents.” 3. “The moments of experiencing connectedness, however fragmentary, signify that the experience of loss is not total and indefinite; an inkling of hope of contact remains – like the hope of the child’s recovery in the PICU – even if it is unlikely, even to the parents themselves.”   “Health care professionals would do well to acknowledge the existential dimension which is part of the human condition.” |
| Fortney, C. A., et al., 2020 | Bereaved parents' perceptions of infant suffering in the NICU | *Qualitative* |  | 21 | “It is challenging to provide supportive intensive care to infants in the neonatal intensive care unit (NICU), giving them every chance for survival, while also trying to minimize suffering for both the infant and parents. Parents who believe their infant is suffering may alter treatment goals based on their perceptions; however, it is unknown how parents come to believe that their infant may be suffering.” | “Parents completed a qualitative interview exploring their perceptions of the level of suffering that their infant experienced at the end of life. Parents whose infant died in a large Midwestern Level IV regional referral NICU from July 2009 to July 2014 were invited to participate. Thirty mothers and 16 fathers from 31 families (31 of 249) participated in telephone interviews between three months and five years after their infant’s death.” | “Four themes emerged from the qualitative analysis: 1) the presence/absence of suffering, 2) indicators of suffering, 3) temporal components of suffering (trajectory), and 4) influence of perceived suffering on parents, infants, and clinical decision making.” | “Parents used signs exhibited by infants, as well as information they received from the health care team to form their perceptions of suffering. The results highlight that there is variability in the way parents perceive suffering in their critically ill infants with life-threatening or life-limiting conditions.”  “Perceived suffering followed different trajectories and influenced the decisions that parents made for their infant.”  “Soliciting parent perspectives may lead to improvements in the understanding of infant well-being,particularly suffering, as well as how parents rely on these perceptions to make treatment decisions for their infant.”  “Suffering is reported to have an effect on parents as well. In this study, some parents reported shared forms of suffering, including infant suffering that caused parent suffering, instances when the infant did not suffer, but the parent did, and when parent suffering was projected onto the infant.” |
| Gear, R., 2014 | Bereaved parents’ perspectives on informal social support: ‘What worked for you?’ | *Qualitative* |  | 18 | “Gear aims to identify and explore the informal social supports that bereaved parents found helpful following the death of their primary school-aged child.” | “Semi-structured interviews. Data transcribed using content and thematic analysis. 10 parents were recruited using the snowball sampling technique. Participants had lost a primary school aged child 4 or more year prior to the study, fluency in spoken English and willingness to participate”.  All parents lived in Australia.  5 areas explored:   1. Personal details 2. Identification of sources of informal social support 3. Description of any helpful characteristics of informal social support 4. What did that mean to you? 5. What advice would you give to help strengthen the informal support of bereaved parents? | 9 common characteristics of helpful informal social support   1. Naturally occurring: pre-existing relationships, familiar places, life activities, contact through surviving siblings’ activities 2. Continuing the bond with deceased child 3. Commitment to the bereaved parent 4. Practical help: showing initiative in identifying possible needs, presenting the bereaved parent with specific offers of assistance, respecting the parent’s choice to accept or reject it, a willingness to be financially generous 5. Gift of time: employer relationships 6. Compassionate transgressions 7. Authenticity 8. Being in tune 9. Keeping the mind occupied   5 themes to strengthen informal social support: challenging society’s ‘me’ attitude, address community awkwardness and misconceptions, recognize informal supporters need support, instigate informal support infrastructure, and strengthen the family unit | “Implications for HP practice in relation to parental bereavement include reorienting health (and other) services to value and support such transgressions, building public policies that do likewise, and strengthening community efforts towards this end.”  “Each parent holds unique values and understandings.” |
| Goldstein, R. D., et al., 2018 | The grief of mothers after the sudden unexpected death of their infants | *Quantitative* | 12.5 |  | “The loss of a child is associated with elevated grief severity, and sudden infant death syndrome (SIDS) is the leading cause of postneonatal mortality in the United States.”  “Lacking preparation or warning, a seemingly healthy infant is discovered dead without apparent cause.”  “The diagnosis of prolonged grief disorder (PGD) has gained broader acceptance and use. Little is known about PGD in mothers after SIDS.” | “Between May 2013 and July 2016, we assessed 49 SIDS-bereaved mothers living in informal settlements near Cape Town, South Africa, and on the Pine Ridge Indian Reservation and 359 SIDS-bereaved mothers affiliated with SIDS parent-support organizations in the United States, United Kingdom, Australia, New Zealand, and the Netherlands.”  “We examined PGD symptom severity and diagnostic prevalence rates between the samples and other significant grief indicators during the period 2 to 48 months after the deaths of their infants.”  “For a PGD scale, we used the Parental Bereavement Questionnaire (PBQ), which is a modification of the Prolonged Grief Disorder 13-Item Scale (PG-13)‍35 that has been adapted for language specific to the loss of an infant.” | “Extremely high, persistent, and similar rates of PGD were found in both samples at every time interval.”  “Daily, intrusive emotional pain or yearning was found in 68.1% of subjects; yearning was significantly associated with emotional pain (P < .0001).”  “Role confusion and anger were the most prevalent symptoms, reported by the majority at every time interval. Rates of role confusion, anger, and diminished trust in others remained constant.”  “The prevalence of avoidance, feelings of meaninglessness, shock, and numbness decreased over time.”  “Acceptance was less prevalent than other grief indicators at every interval.” | “Severe symptoms and heightened risk for PGD was seen in mothers after their infants died of SIDS, with discernible symptom profiles.”  “Separation distress illustrates the intrusive, ongoing consequences of grief in these women. These are painful pangs of grief, and although not a mental disorder per se, they are a cardinal feature of pathologic grief.”  “Although persistent yearning and emotional pain has been found in other research on bereaved parents, the high rates of PGD observed here reveals its prominence during adaptation to this loss.”  “The general emphasis in grief counseling on acceptance as an indicator of the resolution of grief may be problematic for mothers who experience a SIDS loss - because there was no time when acceptance exceeded negative grief indicators, whereas anger and yearning related to their infants’ deaths and absence were found to be present at least weekly throughout the study periods in most of the mothers.” |
| Greg Adams et al, 2013 | Bereaved Caregivers as Educators in Pediatric Palliative Care: Their experiences and impact | *Qualitative* |  | 22 | :With the continuing growth of pediatric palliative care, there is an increasing need to develop  effective training for health care professionals. Bereaved parents have participated in the training of health care  professionals utilizing curriculum from the Initiative for Pediatric Palliative Care (IPPC), but the experience of  bereaved parents as educators has not been studied.” | “Nine bereaved parents and eleven health care professionals were interviewed about their experiences  in a pediatric palliative care education program utilizing the IPPC curriculum. The interviews were recorded,  transcribed, coded and analyzed for themes and subthemes. All interviews were conducted in a private conference room in the hospital. The RA dictated field notes into a recorder  immediately after each interview with parents and  health care professionals. The field notes, transcribed for review and reference, included observations, impressions, and  reactions during the interview.”  Each parent and health care professional completed a corresponding demographic form and data was segregated by code; comparing and contrasting of datum with other data to yield conceptual understanding. | “More benefits than burdens were experienced by both parents and health care professionals from the participation of bereaved parents in the palliative care trainings”.  Benefits :   1. The experience for parents contributed to their meaning-making for both their children’s lives and deaths. 2. Parents and professionals identified mutual learning and increased mutual understanding.   Challenges:   1. Some professionals noted that the presence of parents may have limited the openness of discussion of the professionals 2. Parents acknowledged challenges of emotional management in their participation in the educational program. 3. Both parents and professionals recognized and described challenges involved in working sensitively with patients and families without being overwhelmed by the intensity of situations where children die. | “Parents brought depth and reality to the educational experience and contributed insights about family perspectives that would not otherwise have been present.”  “In addition, this study suggested that not only can the participation of bereaved parents expand and deepen the educational experience for health care professionals, parent participation can also contribute to positive grief adjustment, especially in the area of meaning-making, for the parents.” |
| Haas F , 2003 | Bereavement care: seeing the body | *Non empirical* |  |  | “Generally, it is now accepted that the long term outcomes are better for those who are able to see the body of a loved one as it helps people who are grieving to come to terms with the death.” | Authors opinion using literature review from other studies. | “In general, it is natural to view the body and relatives should be encouraged to do so. Seeing and holding or being allowed to touch and hold helps the bereaved person adapt to the loss and say goodbye.  However, special circumstances should be considered when making this decision- (1) whether or not to show to children (2) if the body is mutilated in a disaster (3) absence of body and associated emotional difficulties (4) neonatal death.” | “More research is needed for certain situations eg mothers’ reactions after stillbirths.The article suggests holding the dead baby prevents bonding with a child born later.  In addition the wishes of those who do not want to vie the body should be respected.” |
| Hechler, T., et al., 2008 | Parents’ Perspective on Symptoms, Quality of Life, Characteristics of Death and End-of-Life Decisions for Children Dying from Cancer | *Quantitative* | 12 |  | “Hechler sought to investigate the situation of children who had succumbed to their malignancy in Germany as perceived by their parents. Interested in 5 areas: 1) symptoms and qual- ity of life, 2) characteristics of the child’s death, 3) anticipation of their child’s death and care delivery, 4) end-of-life decisions and 5) impact of the child’s death on the parents and perceived social support by the health care team.” | “Contacted all existing departments for paediatric oncology in the German federal state of Nordrhein Westfalen and asked them to contact all parents for participation in our study who had lost their child to cancer in 1999 and 2000.  Parents of 48 children (35%) out of 136 agreed to participated in the study and were interviewed using a validated semi-structures interview.” | 1. Symptoms of quality of life in the end-of life care period  - Fatigue and pain were the most common - Fatigue, loss of appetite, dyspnoea, and anxiety were not successfully treated according to parents  1. Characteristics of the child’s death  - 88% of parents chose ‘home’ to be the most appropriate location for their child’s death - Only 48% of the children died at home  1. Anticipation of their child’s death  - 64% discussed end of life decisions with the healthcare team - 50% of parents reported that upon discussion with the health care team did they anticipate that their child would not have a realistic chance of surviving. 13 observed new symptoms in children, 6 reported to have had a sense of impending doom, while 2 observed a change in child’s behaviour.  1. Impact of the child’s death on parents  - 15% of parents were not contacted by the healthcare team following child’s death - 29 observed significant changes in religious preference, employment, partnership or marital status, social contacts to friends or relatives   15 reported significant financial burdens | “Psychological symptoms (e.g. anxiety) are frequent symptoms and cause severe suffering for children.”    Need to investigate potential barriers in the communication between parents and the team to optimize end of life decisions and hence parents’ long term distress.  There is still a lack of contact from HCPs following child’s death, hence there is a need to have guidelines. |
| Hedayat, K. (2006) | When the spirit leaves: childhood death, grieving and bereavement in Islam | *Non empirical* |  |  | “It is important for healthcare workers to understand the dynamics associated with bereavement, especially when the family comes from a non-Western culture. Hedayat seeks to explain what Islam is, who is a Muslim, where they live, and what they believe and practice. It also explains how Islamic beliefs contextualize the meaning of life and death for Muslims and how they exhorted to grieve upon a child’s death.” | NIL | - Customs before, during and after death were discussed in detail - Death of child may have one of 3 meanings: test of patience for parents, a divine desire of a more pious child to be born in place of the cur- rent child, or, as an act of mercy to parents and the child with a guarantee of entry into Heaven for all three without standing for judgment. - Islamic prescriptions for grief include holding the deceased child, crying for them, and acknowledging that they were a gift from God that God has reclaimed   No concept of pathologic bereavement or grief in Islam as long as the person can engage in daily tasks of living 🡪 those who are unable to are encouraged to seek solace in religious teachings and seek appropriate counselling and medication | “There is a need to understand how different cultures have different impact on how a family grieves. HJCPs should be aware of these differences and respect the culture.” |
| Hilkka Laakso et al., 2001 | Mothers' grief following death of child | *Qualitative* |  | 17 | “Research has shown that caring for a dying child is among the hardest and more demanding tasks in nursing, because the staff are forced to manage their heavy work with inadequate skills and experience. This article deals with the findings of a recent study, the purpose of which was to analyse the mother's grief and coping with grief following the death of a child under the age of 7 years.” | “Data were collected from mothers using a survey (n = 91) and an interview (n=50). As the topic was very sensitive ethically and emotionally, survey data were collected first the mothers were asked to give their consent to taking part in an interview. The study employed both quantitative and qualitative methods. The data were analysed using statistical methods and content analysis. However, only the qualitative part of the study is presented in this article.” | “The findings show that nursing staff had skills to support grieving mothers, but that there were many feelings and experiences of grief that remained unidentified by staff. The staff's ability to meet the mothers' individual needs while the child was in hospital and after the child's death was inadequate. The information received from staff was perceived to be insufficient or offensive to mothers.” | “The development of basic and further education and of various support measures would enable the staff to better cope with their work. Focusing on interactive skills and meeting the patient's individual needs using reflective practice would improve the quality of care. Communication and collaboration between different occupational groups should be promoted, because mothers were dissatisfied with dissemination of information, and ambiguous responsibilities between different occupational groups hampered the acquisition of information.” |
| Holston, J. T. 2015 | Supporting families in neonatal loss: relationship and faith key to comfort | *Descriptive* |  |  | “Around 20,000 neonatal deaths occur each year, many from congenital heart defects such as hypoplastic left heart syndrome. Nurses are on the frontline of caring for families experiencing neonatal loss. Careful spiritual and cultural assessment, attention to beliefs, focusing on relationship, and helping families create legacy can assist with grieving and making meaning out of loss.” | Case Report | NIL | NIL |
| Ivana M. M. van der Geest, et al, 2015 | Talking about Death with Children with Incurable Cancer: Perspectives from Parents | *Qualitative* |  | 18 | “To investigate the rationale and consequences associated with a parent’s decision to discuss death  with a child with incurable cancer.” | “All parents who lost a child to cancer from 2000 through 2004 either during or after receiving treatment at the Erasmus Medical Center-Sophia Children's Hospital were invited to complete questionnaire. In addition to the questionnaire, parents were asked open-ended questions about the reason for not talking/ talking to their child about death. Parents were asked whether they had discussed the impending death with their child, whether they reflected on this discussion positively, their reasons for not discussing death with their child, and the manner in which the conversation regarding death occurred. Themes were identified using a framework approach.” | Approximately two-thirds of parents in our Dutch cohort chose not to talk with their terminally ill child regarding the child’s impending death. The following themes were identified:   1. The parents’ inability to discuss the impending death; 2. The parents’ desire to protect their child; 3. Views regarding talking with children 4. Parents’ views of child characteristics 5. The child’s unwillingness to discuss the subject 6. Lack of opportunity to talk 7. The child’s disability.   The parents who did discuss death with their child generally used symbolic and/or religious narratives, or they had brief, direct conversations regarding death. The majority of parents felt positive regarding their decision about whether to talk with their child about his/her impending death. | “There are sensitive and complex issues surrounding these conversations, indicating that there may be a role for clinicians in supporting parents.”  “Parents should not avoid discussing death with the child simply to avoid an emotional response by the terminally ill child, particularly when the child and family have adequate support.”  “However, it should be noted that not discussing the subject of death with their parents may be the best option for some children, and the decision regarding whether—and how—to talk with a child about death should be tailored based on individual context.” |
| Jay M. Milstein 2003 | Detoxifying Death in the Neonate: in Search of Meaningfulness at the End of Life | *Commentary* |  |  | “At the end of life, when curative measures have failed to conquer the terminal nature of a disease, it is imperative to introduce healing measures that deal with the human suffering associated with illness. When dealing with critically ill neonates with lethal conditions, it may be necessary to institute healing measures at the beginning of life. In such cases, healing measures must incorporate customary palliative measures but should not be limited to them.” | Commentary | “The purpose of this paper is to describe two families’ approaches to palliation at the end of life in their newborn infants with Trisomy 18, a genetic abnormality usually associated with a markedly decreased life expectancy. We shall consider the concepts of interference versus intervention as we examine subtle medical differences between the two cases. We will address medical, legal, and ethical issues in each case, but special attention will be given to the provision of compassionate care. People face physical, mental, emotional, and spiritual challenges as they go through life.” | “The families’ approaches with their infants at the end of their lives may support the premise that the latter two challenges, emotional and spiritual, confront us the most at the end. Encouraging families to engage with their dying infants helps detoxify the experience and make it more meaningful.” |
| Jay M. Milstein, & Bonnie Raingruber 2007 | Choreographing the End of  Life in a Neonate | *Commentary* |  |  | “Mothers and fathers experiencing losses in the perinatal period may manifest different degrees of resolution of their bereavement even after the subsequent birth of a healthy infant. These differences may relate to the different degrees of physical, emotional,and cognitive attachment to the lost child  that the mothers and fathers may have.” | Commentary – A recollection of interactions between healthcare team and parents | “Providing some guidance to patient/family dyads, while simultaneously respecting their autonomy, may be helpful to facilitate healing and meaning construction during the process of bereavement. In applying an integrative universal paradigm of care when curative measures elude us, healing measures become of paramount importance.” | “With respect to Figure 2: In the yin/yang duality model of care, the white area, yang, represents the curing elements of care, and the black area, yin, represents the healing elements of care. The course through a disease and illness is represented by the scroll running from the top that corresponds to the onset to the bottom that corresponds to the end. The 2 horizontal lines demonstrate the shifting balance or duality between curing and healing at different stages of the disease and illness.” |
| Jayne Price et al., 2011 | Living through the death of a child: A qualitative study of bereaved  parents’ experiences | *Qualitative* |  | 19 | “Losing a child is a traumatic and life-changing event for parents. The way a child dies is critical to parental adaption.Caring for their child at the end-of-life is complex and multidimensional .This paper explores parental responses to their childs’ illness. “ | “Cross-sectional qualitative study. Semi-structured in-depth interviews with a purposeful sample of 25 recently bereaved parents.” | - Parents’ responses to their child’s illness are driven by their overwhelming need to actively ‘do’ something purposeful for the child, wider family and themselves. - Four analytically distinct processes were identified in the responses of parents to the death of a child. These are referred to as ‘piloting’, ‘providing’, ‘protecting’ and   ‘preserving’. These are central to parents’ achievement of this active ‘doing’, enabling coping.   - Parental focus on piloting, providing, protecting and preserving is evident throughout the entire trajectory of their child’s illness, death and beyond. | “Acknowledgement of their centrality of ‘doing’ through piloting, providing, protecting and preserving to parents’ coping, can help nurses and other health and social care professionals better understand parents’ responses, enabling them to ensure that the provision of appropriate and timely support is a pivotal part of their practice. Facilitating the capacity of parents to ‘do’ is central to coping with the stress and uncertainty of living through the death of a child. The provision of informational, instrumental and emotional support by health care professionals in the context of ‘doing’ is core to quality palliative care.” |
| JoAnne M. Youngbluta et al., 2017 | Parent Grief 1–13 Months After Death in Neonatal and  Pediatric Intensive Care Units | *Quantitative* | 13.5 |  | “Objective: Describe changes in mothers’ and fathers’ grief from  1 to 13 months after infant or child neonatal or pediatric intensive  care unit death and identify factors related to their grief.” | “Mothers (n = 130) and fathers (n 1=52) of 140 children (newborn–18 years) completed the Hogan Grief Reaction Checklist at 1, 3, 6, and 13 months postdeath.” | “Grief decreased from 3 to 13 months for mothers and from 3 to 6 months for fathers.  Grief intensity of mothers of deceased adolescents was consistently greater than that of mothers of deceased infants.  Mothers of children with brain death reported greater despair at all time points and greater panic, blame and anger, and detachment at 6 months than mothers whose child died after unsuccessful CPR efforts or withdrawal of treatment. Research in this area has not been reported.” | “In this study, panic, despair, and disorganization were more intense for mothers than fathers in the same family, which is consistent with findings of others. In some studies, mothers and fathers report that they manifest their grief differently. Perhaps this finding is related to mother–father differences in degree of openness or level of denial.  In conclusion, mothers’ and fathers’ grief intensity may not coincide, resulting in different needs during the 13 months after infant or child death.” |
| Jodi E. Mullen et al., 2015 | Caring for Pediatric  Patients’ Families at the  Child’s End of Life | *Descriptive* |  |  | “Nurses play an important role in supporting families who are faced with the critical illness and death of their child. Grieving families desire compassionate, sensitive care that respects their wishes and meets their needs.  Families often wish to continue relationships and maintain lasting connections with hospital staff following their child’s death. A structured bereavement program that supports families both at the end of their child’s life and throughout their grief journey can meet this need.  The practical strategies outlined in this article may enable nurses to confidently communicate with the child’s family during the dying process and after the child’s death, while providing a profound and meaningful experience for the family.” | Case study – “We begin by presenting a case that illustrates a family’s response when faced with such a life-altering event and the challenges nurses face in caring for and communicating with the family throughout the end-of-life experience. Next, we review practical strategies that nurses can use to communicate with patients’ families at the end of a child’s life. We also provide additional strategies to provide compassionate, sensitive care that respects a family’s wishes and attends to the needs of the family. Finally, we describe a bereavement program designed to facilitate lasting communication with the grieving family.” | Support during illness and at the time of death:   - Communication: establish rapport, establish a relationship with the family through inquiry and reflection; Nonverbal communication is a critical element in supporting grieving families - Nurses can acknowledge a family’s hope by sharing a commitment to the child’s well-being with a wish-worry statement, rather than discounting the family’s feelings. - Early integration of palliative care resources - Special consideration of a patient’s siblings, who are markedly affected by the critical illness and death of their brother or sister.   Support after childs’ death   - Asking open-ended questions such as “What can I do for you at this moment?” encourages family members to express what is immediately important to them. - Family members should be offered the opportunity to   view the body   - Nurses can provide an appropriate space for grieving families - Giving a family tangible mementos   Ongoing Support and Lasting Communication | “A bereavement program that supports the hospital staff ’s connection with the family after the child’s death attends to the family’s grief journey and acknowledges the value of their child’s life and unique legacy.” |
| Jonas, D., et al., 2018 | Bereavement After a Child's Death | *Descriptive* |  |  | “The death of a child can have an impact on various members of a child’s family and community.Often this loss has the most direct impact on the child’s primary caregivers, immediate family members, siblings, and peers. It may also have an impact on health care providers as well as additional members of the child’s community, such as those within their school, church congregation, and peers.” | “Terms used: Parent bereavement, Sibling bereavement , Palliative care, Pediatrics, Grief, Pediatric death Provider grief, Memory making” | “Family experience   - Family Modeling of Emotional Processing: Creating the space for honest and loving language, even when the words are imperfect and difficult to find. - Holidays/Anniversaries: As a family reconstructs the meaning of their child’s life and death, they can create lasting legacy through the addition of new ways to honor their child in the hopes of off-setting the potential of complicated grief.”   “Parent experience   - Spiritual impact: To optimize the spiritual and religious needs of patients/families at end of life and in bereavement, a thorough assessment of spiritual needs and coping strategies is imperative. - Unhelpful Spiritual Responses: Often deeply religious friends or family members encourage parents to pray constantly and not give up, making promises that if they are faithful enough, God will heal their child. This unhelpful spiritual belief/support often causes major spiritual distress if a child dies and often negatively affects spiritual coping in bereavement. - Emotional responses: Guilt, self-blame, regret, evolving perspectives/ relationships, parenting additional children”   Sibling experience, family coping mechanisms and provider experience was also expounded on in this paper. | “Grief after the death of a child can have an impact on bereavement in a multitude of ways, including spiritually, emotionally, developmentally, and functionally.” |
| Karen S. Heller, et al , 2005 | Continuity of Care and Caring: What Matters to Parents of Children with Life-Threatening Conditions | *Qualitative* |  | 23 | “This article presents parents’ perceptions regarding continuity and coordination of care of children with life-threatening conditions as revealed through qualitative analysis of interviews with 36 bereaved parents of children who died after receiving care at three geographically dispersed teaching hospitals in the United States.” | “This was a interview study of bereaved parents using a 23 item guided interview protocol. Transcripts of the tape-recorded interviews were analyzed for themes and topics mentioned by parents in the interviews using a well-established qualitative research approach to data analysis.” | “There were five overarching themes regarding continuity of care that emerged from analysis of the interviews.”  There were:   - Continuity Builds Relationships and Promotes Caring - Parents Want Clinicians to Know Them and Their Children as Individuals. Being Known Increases Confidence About The Quality of Care and May Reduce Hypervigilance - Continuity Encourages Sharing of Expertise and Information About the Child - Poor Continuity of Care Within and Across Settings Creates Confusion and Frustration - Being There Through the Child’s Death and Parents’ Bereavement Provides Great Comfort   Parental concerns about and experience of continuity of care were framed primarily in terms of the quality and continuity of relationships with healthcare providers throughout a child’s illness and death and the continuity and consistency of information that they received about their child’s condition and care. | “Continuity in relationships was perceived as key in ensuring that clinicians knew and cared about the child and parents, which in turn contributed to parents’ confidence that their child would receive the best possible care. In the absence of continuous, caring relationships with staff, parents reported frustration, hypervigilance, and mistrust about the quality of care that their child received.” |
| Karin Zimmermann, Eva Bergstraesser, 2016 | When parents face the death of their child: a nationwide cross-sectional survey of parental perspectives on their child’s end-of life care | *Quantitative* | 15 |  | “Parents facing the death of their child have a strong need for compassionate professional support.  Care services should be based on empirical evidence, be sensitive to the needs of the families concerned, take into account the heterogeneity within the medical field of paediatrics, and fit into the local health care system. We need to better understand the perspectives of parents facing the death of their child in order to guide further development and evaluation of specialised paediatric palliative and end-of-life (EOL) care services.” | “Questionnaire survey to assess the EOL care perspectives of a Swiss population-based sample of bereaved parents who had lost a child due to a cardiac, neurological or oncological condition, or during the neonatal period in the years 2011 or 2012. A survey instrument, the Parental PELICAN Questionnaire (PaPEQu) was developed by the PELICAN study group. Four slightly different versions for the four diagnostic groups (cardiology, neonatology, neurology, and oncology) were created to account for differences in illness trajectories between the groups. The parental perspective was assessed with a newly developed and tested instrument that was structured according to six evidence-based quality domains.The six domains are in accordance with existing evidence and include: support of the family unit, communication, shared decision making, relief of pain and other symptoms, continuity of care, and bereavement support. For experience-related scale items, the response option was either a 7-point (0 to 6) with varying end-point anchors (“never-always” , “not clear at all-very clear” , “not honest-honest”) , or a 5-point Likert-type (1 to 5), where respondents indicated the extent to which they agreed with the statement.” | “There was a questionnaire response rate of 89 %.  Parents of deceased neonates represented the largest group, followed by parents from the neurology, oncology and cardiology group.”  “Parents rated experiences with their child’s EOL care as generally positive.”  “Parents of children with cancer rated their experiences during their child’s EOL care highest, while parents of children with a neurological condition rated their overall experiences lowest.”  “The cardiology, neurology and oncology groups all showed the same pattern in experience scores across the six quality domains.”  “Parents of neonates showed a different pattern, with a significantly lower score in the domain “shared decision making” and a high score in the domain “relief of pain and other symptoms”.” | “Parents of children with neurological impairments face many challenges. Symptom management can be a source of distress for parents, as the children are mostly non-verbal and the potential for suffering is high due to a variety of impairments. This makes them dependent on a variety of different care services, which creates a highly complex care environment with a great need for continuity and coordination.”  “Parents of neonates reported significantly lower experience ratings related to shared decision-making. As these parents mostly face a decision to withdraw life-sustaining measures, particular attention should be paid to shared decision-making processes.  The integration of specialised paediatric palliative care has the potential to minimise lost opportunities to support and assist parents.” |
| Kathless O'Connor and Maru Barrera, 2014 | Changes in Parental Self-Identity Following the Death of a Child to Cancer | *Qualitative* |  | 21 | “This study explored parental self-identity at 6, 12, and 18 months following the death of a child to cancer.” | “Semi-structured interviews were analyzed using qualitative methodology.” | “Two patterns of parental self-identity emerged: identity reintegration, characterized by 6 associated themes (e.g., positive reframing, focusing on surviving children); and identity disintegration, characterized by 7 associated themes (e.g., negative perceptions of social support, self-destructive thoughts and behaviors). Patterns were stable from 6 to 12 months, but a shift towards identity disintegration was observed at 18 months.” | “A unique and important contribution of this study was its capacity to document fluctuations in self-identity over time. This feature proved especially important at the 18-month mark, when most parents originally displaying characteristics of reintegration shifted to present with characteristics of disintegration. These changes over the first 18 months postdeath appear consistent with Romanoff and Terenzio’s (1998) proposal that the period of time immediately after parents assume bereaved status is frequently characterized by a transitional phase in which the parent may adopt or experiment with different identities.  In this study, the social context emerged as a key factor that could facilitate or prevent development of reintegration during the first 18 months postdeath.  These findings suggest a need to support bereaved parents’ well-being beyond the first year post-death.” |
| Keim, M. C. et al, 2017 | Parent Distress and the Decision to Have Another Child After an Infant’s Death in the NICU | *Mixed Methods* | 15.5 | 18 | “To examine associations among parent perceptions of infant symptoms/suffering, parent distress, and decision making about having additional children after an infant’s death in the NICU.” | “Design: Mixed-methods pilot study incorporating mailed surveys and qualitative interviews.  Setting: Midwestern Level IV regional referral NICU.  Participants: Participants were 42 mothers and 27 fathers whose infants died in the NICU.  Methods: Parents reported on infant symptoms/suffering at end of life and their own grief and posttraumatic stress symptoms. Qualitative interviews explored decision making about having additional children.” | “Approximately two thirds of bereaved parents had another child after their infant’s death (62% of mothers, 67% of fathers). Mothers who had another child reported fewer infant symptoms at end of life compared with mothers who did not (p ¼ .002, d ¼ 1.28). Although few mothers exceeded clinical levels of prolonged grief (3%) and posttraumatic stress symptoms (18%), mothers who had another child endorsed fewer symptoms of prolonged grief (p ¼ .001, d ¼ 1.63) and posttraumatic stress (p ¼ .009, d ¼ 1.16). Differences between fathers mirrored these effects but were not signiﬁcant. Parent interviews generated themes related to decision making about having additional children, including Impact of the Death, Facilitators/Barriers, Timing/Trajectories of Decisions, and Not Wanting to Replace the Deceased Child.” | “Having another child after infant loss may promote resilience or serve as an indicator of positive adjustment among parents bereaved by infant death in the NICU. Prospective research is necessary to distinguish directional associations and guide evidence-based care.” |
| Knighting, K., et al., 2019 | A Network Approach to Neonatal Palliative Care Education: Impact on Knowledge, Efficacy, and Clinical Practice | *Technical report* |  |  | “This article presents the evaluation of the workshops including their impact on the participants’ perceived efficacy in caring for the dying neonate (self-efficacy) and perceived attitudes and outcomes in caring for the dying neonate (thanatophobia/effectiveness), along with any longitudinal impact on their clinical practice.” | “This article presents a mixed methods, sequential, explanatory design evaluation of 1 day palliative care education workshops delivered using a network-wide approach to multidisciplinary professionals.  Workshops were delivered by healthcare professionals and bereaved parents and evaluated using questionnaires, adapted for neonatal staff from standardized measures, and follow-up interviews.” | One of the mums said how important she felt seeing all of those faces that had cared for her child… following which one participant said ‘do you know what, this lady said how valuable she found to see all those faces, obviously we can’t allow every member of staff to go to a child’s funeral but I think we need to you know try wherever possible for the people who have had an impact on that family to be able to attend.’ | “Parent stories were identified as a very powerful component of the training, with lasting impact on participants.”  It also highlighted the Importance of understanding parents’ needs and wishes for palliative and end-of-life care, including the opportunity to make memories. |
| Leemann, T., et al., 2020 | Differing needs of mothers and fathers during their child's end-of-life care: Secondary analysis of the "paediatric end-of-life care needs" (PELICAN) study | *Quantitative* | 15 |  | “Mothers and fathers are severely challenged when providing care for their terminally ill child at the end of life. Caregiving needs have been studied predominantly in mothers. Differences in caregiving needs between mothers and fathers during their child’s end of life have not, however, been explored so far. This knowledge is of importance to best meet individual parental needs in paediatric end-of-life care.” | “Secondary analysis of a quantitative survey on parental needs during their child’s last 4 weeks of life, collected in the Swiss multicentre “Paediatric End-of-Life Care Needs” (PELICAN) study. Caregiving needs of mothers and fathers (parental dyad) who had lost a child due to a cardiological, neurological or oncological disease or during the neonatal period in the years 2011–2012 were retrospectively assessed using a questionnaire representing six evidence-based quality domains of paediatric palliative and end-of-life care. Seventy-eight parental dyads were included in this analysis. This study questionnaire was developed to assess bereaved parental experiences and needs related to their deceased child’s EOL care.” | “Differences between mothers and fathers were mostly found around needs to be supported as a family. In all, 28 out of 34 needs-related questionnaire items were scored higher by mothers than by fathers, indicating higher importance for that need to be met.” | “The results indicate that these differences might relate to different caregiving roles and gender-specific coping strategies.”  To mothers it is more important to be able to stay close to their child at night, to be able to ask questions all the time and to have the same nurses providing care. Fathers, facing conflicts between work and care requirements, have a greater need for respite from care.  “To best meet parental needs in paediatric end-of-life care, particular attention should be paid to both mothers and fathers and their specific caregiving roles, and should be supported in their individual coping strategies.” |
| Leigh A Donovan et al, 2015 | Hospital-based bereavement services following the death of a child | *Descriptive (Review)* |  |  | “There has been a breadth of research on the grief experience of parents following the death of a child. However, the role and impact of hospital-based bereaved services remain unclear.  The paper aims to identify services offered to bereaved families in perinatal, neonatal, and pediatric hospital settings and summarize the  psychosocial impact of these services and published recommendations for best practice hospital-based bereavement care.” | “Systematic review of qualitative, quantitative, and mixed method studies guided by the Preferred Reporting Items for Systematic Reviews and Meta-Analyses checklist and methodological quality appraised in accordance with the Mixed Method Appraisal  Tool, MEDLINE, EMBASE, Cumulative Index to Nursing and Allied Health, and PsychINFO were searched to find studies describing hospital-based bereavement services/interventions for parents, siblings, and grandparents.  In all, 14 qualitative, 6 quantitative, and 10 mixed method studies were identified. Nine descriptive articles were also included.” | Qualitatively, family members described feeling cared for and supported by staff, a reduction in sense of isolation, and improved coping and personal growth.  Quantitatively, bereavement services have most effect for parents experiencing more complex mourning.  Recommendations for best practice:   - Develop a formal model of care that is theoretically driven and evidence based - Provide effective communication and continuity of care through diagnosis, treatment, palliative,   and bereavement care   - Provide a range of interventions for the “whole   family” and flexibility in service delivery   - Ensure collaboration between family,   community, and hospital treatment unit   - Provide support, supervision, and education for staff | “There is a role for transitional hospital-based services/interventions for families in the lead up to and following the death of a child.  A flexible menu of interventions with collaboration between hospital, community, and families was most likely to meet the unique needs of parents, siblings, and grandparents. Timeframes for the offering of and withdrawal of bereavement care remain ambiguous due to the unique nature of parental grief. A lack of dedicated funding and a clear evidence base from which healthcare professionals guide development of bereavement support programs inhibits best practice.  Further mixed method research is required to inform best practice bereavement care guidelines in the perinatal, neonatal, and pediatric hospital settings.” |
| Levick, Judy et al, 2017 | NICU Bereavement Care and Follow-up Support for Families and Staff | *Qualitative* |  | 16 | “Background: Experiencing the death of an infant in the neonatal intensive care unit (NICU) affects both families and staff, creating challenges and opportunities for best practices.  Purpose: This practice-based article describes a comprehensive approach to delivering bereavement services to NICU families, as well as education and support to NICU staff.” | “Bereaved NICU parent and staff survey feedback, including quotes describing individual experiences and suggestions for improved service delivery.” | “Bereaved NICU families and caregivers find meaning and purpose in the act of creating keepsake memories at the time of the infant’s death. Mutual healing takes place with subsequent, individualized follow-up contacts by staff familiar to the bereaved parents over the course of a year.” | “Implications for Practice: Those staff involved in the care of a NICU infant and family, during and after the infant’s death, attest to the value in providing tangible keepsakes as well as continuing their relationship with the bereaved parents. An effective administrative infrastructure is key to efficient program operations and follow-through.  Implications for Research: Studying different methods of in-hospital and follow-up emotional support for NICU bereaved families. Identifying strategies for staff support during and after NICU infant loss, and the impact a formal program may have on staff satisfaction and retention.” |
| Lewis-Newby, M., et al., 2018 | When a Child Dies in the PICU Despite Ongoing Life Support | *Non empirical* |  |  | “This article explores death and end-of-life (EOL) care in PICUs specifically in the setting of ongoing life-saving or life-prolonging interventions. In relatively resource available PICUs, advances in treatment of critically ill children have led to greater than 97% survival (1). In the context of this relatively low mortality rate, PICU clinicians and families may choose to remain focused on life-saving goals even when the child is facing a poor prognosis and high risk of death (2). This focus on curative goals not uncommonly inhibits provision of high-quality EOL care.” | “In this article, we describe three common clinical scenarios when curative goals of care are pursued despite a high likelihood of death and explore the challenges to providing high-quality EOL care in each setting.” | First scenario deaths occur in the setting of unlimited life-saving interventions with the family and medical team in agreement about the goals of care. The highest levels of life support available are employed - Death commonly occurs after unsuccessful cardiopulmonary resuscitation (CPR).  In this second scenario, deaths occur after withdrawing or forgoing some, but not all, potentially life-saving interventions. EOL care practices and palliative care consultation may or may not be merged concurrently with life-prolonging and attempted curative interventions.  In the third scenario, the family and the medical team are not aligned regarding goals of care. In recent decades, most commonly the family requests continued life-prolonging therapies despite great certainty in a poor prognosis and high likelihood of death. Despite strong recommendations from the medical team to forgo or withdraw life support, the family typically requests that “everything be done” to prolong life. | Reasons parents may chose to pursue ongoing life-sustaining efforts despite poor prognosis.   - More Time for Decision-Making - More Time With Child - Cultural and Religious Values - Values and Perspectives on a “Good Death” - Uncertainty in Prognostication - Perception of Parental Role - Perception of the Do Not Resuscitate or LimitationOrder - Unclear Communication From Clinical Team - Lack of Trust in Medical Team   Through an intentional exploration of self and others’ perspectives, values, and goals, and working toward finding commonality in order to align with each other, conflict in EOL care may lessen, allowing the central focus to remain on providing optimal support for the dying child and the family.  Merging curative interventions and optimal end-of life care is possible, important, and can be enabled when clinicians use creativity, explore possibilities, remain open minded and maintain flexibility in the provision of critical care medicine. |
| Lord, S., et al., 2020 | Assessment of Bereaved Caregiver Experiences of Advance Care Planning for Children With Medical Complexity | *Qualitative* |  | 21 | “Advance care planning (ACP) is the process of discussing values and preferences for care to help inform medical decision-making. Children with medical complexity (CMC) often have a shortened life span with an unpredictable clinical course and timing of death; however, there is a paucity of literature that describes the experience of ACP from the perspective of bereaved family caregivers of CMC.  Objective: To explore the experiences of bereaved family caregivers with ACP for CMC.” | “This qualitative study included 12 interviews with 13 bereaved family caregivers of CMC whose deaths had occurred in the 5 years before study commencement (2013-2018). Participants were recruited at a single tertiary care pediatric center; CMC were treated by the Complex Care or Long-term Ventilation clinic in Toronto, Ontario, Canada. Data were collected from July to October 2018. Thematic analysis with an inductive approach was used.  A total of 13 family caregivers were interviewed in 12 interviews, all of whom were parents (12 [92%] women, 1 [8%] man) of a deceased child (aged 7 months to 12 years).” | “Themes were divided in the 3 following categories, which align with the Donabedian model for health service quality: (1) structure of care, (2) ACP process, and (3) end-of-life experience. Notable subthemes for this population included the importance of accounting for parental expertise in the child’s care, recurrent experiences with life-threatening events, relative shock of the timing of death, and the multiple losses that caregivers experienced.” | Examples of themes:  Health care team support (asking about who their daughter was beyond her condition; having members of the team that are going to be involved in the lifetime involved early)  ACP process - understanding family and patient context, appropriate approach to ACP discussions (sooner and smaller chunks of conversation instead of having one big one; appropriate setting; same person continuing the conversation so that parents do not need to repeat themselves; compassionate approach)  End of Life care outcomes - relative shock, location of death, multiple losses (of medical family as well), grief and bereavement  Participants emphasized the importance of involving trusted health care professionals and incorporating parental expertise to guide advance care planning. The relative shock parents experienced regarding the timing of the child’s death, despite recurrent experiences with life threatening events, and the multiple losses they experienced when the child with a large health care team died were important themes. |
| Lykke, C., et al., 2019 | Anxiety and Depression in Bereaved Parents After Losing a Child due to Life-Limiting Diagnosis: A Danish Nationwide Questionnaire Survey | *Quantitative* | 14.5 |  | “To investigate anxiety and depression in bereaved parents during their child’s life-limiting illness and imminent death and three to five years after the loss to target future interventions.” | “A Danish nationwide cross-sectional questionnaire survey. From 2012 to 2014, a register-based study identified causes of deaths of 951 children aged zero to 18 years. Potential palliative diagnoses were classified according to previously used classification. A total of 402 families were included. A modified version of the self-administered questionnaire ‘‘To lose a child’’ was used. Non-response surveys identified reasons for lack of response.” | Anxiety:  “Sixty five percent of mothers and 63% of fathers reported moderate-to-severe anxiety during the child’s illness. However, three to five years after their loss anxiety had decreased markedly. High prevalence of anxiety may likely be associated with increased distress because of the child’s end-of life trajectory. Some of the parents may even have developed posttraumatic stress disorders.”  Depression:  “Thirty-five percent of mothers and 39% of fathers reported moderate-to-severe depression during the child’s illness; three to five years after the loss they were suffering equivalently from depression. Anxiety was reduced substantially at the time of the survey, whereas depression remained unchanged”  “The Center for Epidemiologic Studies Depression Scale indicated that severe depression was significantly associated with lower education and being unmarried.” | “The reporting of anxiety during the child’s illness and prolonged depression in bereaved parents three to five years after the loss indicates a potential need for psychological interventions. In the process of implementing specialized pediatric palliative care in Denmark, our findings should be considered for future treatment programs.”  “The reported experience of anxiety and prolonged depression years after loss indicates the need for interventions to support and treat parents during their child’s life-limiting illness and after the loss. Future studies should inform who is at risk and may identify resilience factors that protect parents from psychosocial comorbidities and potential family disruption.” |
| Malin L€ovgren et al., 2016 | Parents' Experiences and Wishes at End of Life in Children with Spinal Muscular Atrophy Types I and II | *Quantitative* | 11 |  | “Spinal muscular atrophy (SMA) is a rare, autosomal-recessive disorder characterized by slowly progressive muscle weakness.1 SMA is classified into 4 grades of severity in which the most severe form, type I, presents by definition during the first 6 months of life, and death usually occurs within the first 2 years of life if there is no aggressive respiratory support.  Type II presents slightly later, at 6-18 months, and shortens life expectancy. There is presently no cure for any form of SMA.  Objective: to explore experiences and wishes of bereaved parents concerning end-of-life care for their child with severe spinal muscular atrophy.” | “This study is based on a nationwide survey conducted in 2013 on parents of deceased Swedish children who were born between 2000 and 2010 and later diagnosed with spinal muscular atrophy type I or II (n = 48). The questions used in this study covered location of death (LoD), support from health care staff, and parents’ wishes and concerns about their child’s end-of-life care.” | “An almost equal number of mothers and fathers participated in the study. Parents’ mean age was close to 41 years at time of follow-up and all were married or cohabiting with the child’s mother or father. All but 3 children had SMA type I and the mean age at time of death was 9.5 months.  One-half of those who had wishes about LoD (16/32) wanted their child to die at home, rather than at the hospital. All of those who wanted the child to die at the hospital had their wishes fulfilled. Among those who wanted the child to die at home, 10 of 16 got their wish. Among parents who talked with a physician about how they wanted their child to pass away (n = 26), all but 2 had their wishes fulfilled.  Thirty-six parents (75%) reported that their child had siblings: 12 reported that the sibling was too young for professional psychological support, and only 4 of the remaining 24 siblings received such support after the death of their brother or sister.” | “Parents’ communication with the physician about their wishes and concerns regarding their child’s end-of-life care and preferred LoD contributed to their wishes being fulfilled. The wish of hospital death was fulfilled more often than the wish of home deaths.  Other factors that health care staff need to consider when planning for end-of-life care are whether the child is enrolled in home care, if the ill child has siblings, and the ill child’s well-being. Most parents in this study perceived health care staff as supportive in end-of-life care and after death care.  However, there is still room for improvement; for example, healthy siblings need to be offered psychological support.  A vast majority of siblings did not receive psychological support after death of their brother or sister.  Health care staff need to be more sensitive, listen more attentively to the parents, and give the child with SMA adequate pain relief.” |
| Maria Bjork et al., 2016 | Like being covered in a wet and dark blanket- Parents' lived  experiences of losing a child to cancer | *Qualitative* |  | 23 | “The aim of this study was to illuminate parents' lived experiences of losing a child to cancer.” | “Interviews and a narrative about parents' experiences of losing a child to cancer were gathered from six parents of children whom had participated in a longitudinal study across the child's illness trajectory. The analysis of the data was inspired by van Manen's hermeneutic phenomenological approach.” | “One essential theme emerged: Like being covered in a wet and dark blanket, as well as six related themes: Feeling conflicting emotions, Preparing for the moment of death, Continuing parenting after death, Recollecting and sharing memories, Working through the sorrow and New perspectives in life.” | “There is a need for good palliative care. If not, there is a risk that the parent will perseverate and blame themselves for not being a good parent during the suffering child's last time in life. Meetings with the parents six months and two years after the child's death might facilitate healing through the grief process.  Different supporting interventions can be suggested based on our findings. One intervention is based on the parents' need for support after the child had died.”  “In the future, additional important insights could be provided by focusing qualitatively on whether mothers and fathers experience losing a child to cancer in different ways, as it may lead to more nuanced care for families.” |
| McCarthy, M.C., et al. (2010) | Prevalence and predictors of parental grief and depression after the death of a child from cancer | *Quantitative* | 16 |  | “After the death of a child, parents experience significant and enduring psychological distress. Studies have shown parental bereavement to be associated with more intense and prolonged grief than other types of bereavement. McCarthy seeks to investigate patterns of grief and depression in a sample of parents who child had died of cancer, and to examine factors related to burden of illness and end of life care as potential predictors of parental grief and depression outcomes.” | “Eligible parents were those who child had died between 1996 and 2004, who were English speaking and had no known major psychiatric illnesses. Study conducted at the Royal Children’s Hospital in Melbourne. 65 out of 193 eligible families were approached by completing questionnaires. 58 (89%) completed the questionnaires”:   1. Beck Depression Inventory-Second Edition 2. Inventory of Complicated Grief-Revised 3. Structured interview and self-report questionnaire. Rated on a 4 to 5 point scale, assessing perception of oncologist care, perception of child’s QOL, preparedness of child’s death, economic hardship and patient education levels. | Grief and bereavement-related depression   - Rates of prolonged grief disorder were similar to those reported in other bereaved populations. - 41% of parents met diagnostic criteria for grief-related separation distress - 22% reported clinically significant depressive symptoms   Predictive factors for complicated grief and bereavement-related depression   - Time since death and parental perception of oncologist’s care predicted grief symptoms but not depressive symptoms  1. Perceptions of child’s QOL during the last month, preparedness for child’s death and economic hardship predicted grief and depression outcomes | Parental grief is intense and long lasting hence level of separation distress was extremely high. |
| Meert, K.L., et al., 2001 | Parental coping and bereavement outcome after the death of a child in the pediatric intensive care unit | *Quantitative* | 15 |  | “Parental grief after the death of a child may be influenced by the chronicity of the child’s illness, the quality of care provided, and the parent’s ability to cope. Meert aims to identify aspects of pediatric intensive care and parental coping that have a favourable effect on parental bereavement outcome.” | “Parents who experienced the death of their child in the PICU between January 1 1995 and June 30 1998 were asked to participate in a telephone interview. 57 out of 78 (73%) agreed to follow up survey.” | - Parents’ whose child died acutely had greater intensity of early and long-term grief than those whose child died of chronic illness (p < 0.05, Mann-Whitney U test) - Parents’ physical coping resources and the emotional attitudes of pediatric intensive care unit staff predicted the intensity of early grief   Parents’ cognitive coping resources, the emotional attitudes of staff, and the adequacy of information provided to parents predicted the intensity of long-term grief | “A caring emotional attitude displayed by the PICU staff has beneficial short term and long term effects on parental bereavement.” |
| Meert, K.L., et al., 2008 | Parents’ perspectives on physician-parent communication near the time of a child’s death in the pediatric intensive care unit | *Qualitative* |  | 18 | “Communicating bad news about a child’s illness is a difficult task commonly faced by intensive care physicians. Greater understanding of parents’ scope of experiences with bad news during their child’s hospitalization will help physicians communicate more effectively. Meert seeks to describe parents’ perceptions of their conversations with physicians regarding their child’s terminal illness and death in the PICU.” | Secondary analysis of a qualitative interview study.  Audio recorded semi structured telephone interviews.  “Six children’s hospitals in the National Institute of Child Health and Human  Development Collaborative Pediatric Critical Care Research Network.  56 parents of 48 children who died in the PICU 3-12 months before the study (out of 161 deceased children) were interviewed.” | “40 (71%) wanted to provide feedback on the way information about their child’s terminal illness and death was communicated by PICU physicians”   - Most common communication issue identified by parents was the physicians’ availability and attentiveness to their informational needs - Others: honesty and comprehensiveness of information, affect with which information was provided, withholding of information, provision of false hope, complexity of vocabulary, pace of providing information, contradictory information, and physicians’ body language. | “Parents want physicians to be accessible and to provide honest and complete information with a caring affect, using lay language, and at a pace in accordance with their ability to comprehend.”  “Withholding information from parents often lead to false hopes and feelings of anger.” |
| Melin-Johansson, C., et al., 2014 | When a child dies: parents’ experiences of palliative care – an integrative literature review | *Descriptive (Review)* |  |  | It is stressful for pediatric palliative care staff to care for a child with terminal illness; Care requires a great deal of knowledge, understanding and compassion for the child and the parent’s situation.    “Melin-Johansson aims to increase knowledge about parents’ experiences of palliative care when their child is dying or has died due to illness.” | “Using Whittemroe and Knafl analysis process  Scientific papers were searched for in the PubMed, PsycInfo and CINAHL databases. Searches in The Cochrane Library yielded no relevant results. All searches were performed with the keywords Pediatrics and Child or using the filter ‘child 0–18 years'. No time limits for years were used. Total of 52 papers.  9 papers met the inclusion criteria. Analysis was guided by the research question: “What does this study tell us about parents' experiences when a child in the family is dying or has died?”” | 5 categories   1. Genuine communication  - Delivery of information in everyday language - Bad news to be conveyed directly and honestly from a person known by the parents - HCPs ask the same question several times: documentation and reporting of care plan between providers - Conflicting or mixed information from different HCPs were stressful - Conversations about death - Involved in decision making at the end of life  1. Sincere relationships  - Empathy and presence - Secure interaction: staff there for family from the beginning to the end and into the mourning period - Uncertainty and abandonment: staff were abrupt or did not listen to concerns; avoided parents - Child centred care: spoke to their child as a unique individual and not as a disease. Respect and meet child’s desires. Include children who were old enough into discussions. - Continuity and availability  1. Respect as an expert  - Important caregivers: equip parents with told and knowledge to continue being the caregivers for their child - Assessment affirmed: staff to listen to these and take them seriously.  1. Alleviation of suffering  - Pain relief  1. Need of support  - Care at home: assistance from staff to feel supported - Respite and relief: temporary respite had to be balanced with time spent with their child - Maintaining normalcy  1. Dealing with grief: creating of mementos after child died, continuing relationships with HCPs | “Parents valued open and honest communication, true relationships with nursing staff, being respected as experts in the care of their child, and the recognition that they needed support.” |
| Meyer, E. C., et al., 2002 | Parental Perspectives on end-of-life care in the pediatric intensive care unit | *Quantitative* | 10.5 |  | “Understanding the dynamics of the family during the dying process is a necessary first step in improving terminal care for children. Meyer seeks to identify priorities for quality end-of-life care from the parents’ perspective in order to incorporate these.” | “Samples parents of children who had died between 1994 and 1996 in the PICUs at Children’s hospital, Massachusetts General Hospital, and Tufts-New England Medical Center in Boston. Patients died after the foregoing of life-sustaining treatment. Parental Perspectives Questionnaire consisted of 28 Likert questions and 5 open -ended questions. Questionnaire elicited parental ratings about quality of communication, adequacy of pain management, end-of-life decision making and social support network. Open-ended question elicted what was most useful and least helpful to parents at the end of their children’s lives, parents’ suggestions to improve the quality of family-staff communication, and their advice for hospital staff members and other parents who face similar situations.” | Of 96 eligible households, 56 completed questionnaires (58%).   1. Among decision-making factors, parents rated the quality of life, likelihood of improvement, and perception of their child’s pain as most important. 2. Twenty percent of parents disagreed that their children were comfortable in their final days. 3. Fifty-five percent of parents felt that they had little to   no control during their child’s final days, and nearly a quarter reported that, if able, they would have made decisions differently. | Families facing medical crises tend to rely heavily on a select social network because it can be too burdensome to keep everyone involved during the child’s illness.  Clinicians can be quickly drawn into the family’s inner circle, especially nurses. After the child’s death, the primary social support available to parents shifted abruptly from that dominated by staff to that provided mostly by family members, friends, and religious support persons.  Reluctance and anxiety on the part of the staff to prognosticate to impart bad news can further limit parents’ opportunities to understand their child’s likelihood of survival.  Parents make decisions about withdrawal of life support based, in part, on how they perceive their child’s pain level. Parents may be more likely to withdraw support, for example, if they perceive their child to be in pain.  Parents who harbour feelings of loss of control and regret about their child’s hospitalizations and circumstances may be at increased risk of unresolved grief. |
| Mitchell, S., et al., 2019 | Parental experiences of end of life care decision-making for children with life-limiting conditions in the paediatric intensive care unit: a qualitative interview study | *Qualitative* |  | 15 | “To provide an in-depth insight into the experience and perceptions of bereaved parents who have experienced end of life care decision-making for children with life-limiting or life-threatening conditions in the paediatric intensive care unit (PICU).” | “An in-depth qualitative interview study with a sample of parents of children with life-limiting or life threatening conditions who had died in PICU within the previous 12 months. A thematic analysis was conducted on the interview transcripts.” | Five interconnected themes were identified related to end of life care decision-making:(1) parents have significant knowledge and experiences that influence the decision-making process.(2) Trusted relationships with healthcare professionals are key to supporting parents making end of life decisions.(3) Verbal and non-verbal communication with healthcare professionals impacts on the family experience.(4) Engaging with end of life care decision-making can be emotionally overwhelming, but becomes possible if parents reach a ‘place of acceptance’. (5) Families perceive benefits to receiving end of life care for their child in a PICU. | “The expertise and previous experience of parents is highly relevant and should be acknowledged. Learning from the experiences and perceptions of families should inform improved policy and practice.”  “End of life care decision-making is a complex and nuanced process; the information needs and preferences of each family are individual and need to be understood by the professionals involved in their care.ACP is not well understood by parents, and appears to be more helpful for some than others.”  “This study highlights the critical importance of a trusted relationship between families and their HCPs. While trust is vital to the relationships between families and HCPs, it is also fragile, and can be easily lost.” |
| Monterosso, L., and Kristjanson, L.J., 2008 | Supportive and palliative care needs of families of children who die from cancer: an Australian study | *Qualitative* |  | 17 | “To obtain feedback from parents of children who died from cancer about their understanding of palliative care, their experiences of palliative and supportive care received during their child’s illness, and their palliative and supportive care needs.” | “Qualitative study with semi-structured interviews. 24 parents were recruited from 5 Australian tertiary pediatric oncology centers. Interview touched on the following major issues which were previously identified in Phase I of study (face to face or telephone interviews)”:   - Parents’ understanding of the concepts of palliative and supportive care - Realization by parents that his/ her child was no longer responding to curative care - Carers who were involved in the provision of palliative care - Most helpful aspects of palliative care provision - Things of greatest importance during the last few months of a child’s life   Hopes for differences for future families | Parents’ understanding of the concepts of palliative care   - Viewed as the ‘beginning of the end’ or a replacement of the unknown - Parents felt that HCPs should explain what the palliative care approach means in practical terms as well as reassurance that the child’s primary carers will continue to be involved in the palliative care process   Practical aspects of palliative care provision and priorities in end of life care   - Authentic and honest relationships between HCPs and parents were valued   Need to include children and adolescents in decision making | “Parents construed palliative care negatively as an independent process at the end of their children’s lives rather than as a component of a wider and continuous process where children and their families are offered both curative and palliative care throughout the cancer trajectory.”  HCPs need to better understand the concept of palliative care, and factors that contribute to honest, open, authentic and therapeutic relationships of those concerned in the care of the dying child 🡪 acceptance of the integration of palliative and supportive care in routine cancer care |
| Morris, A.T., et al., 2016 | The indirect effect of positive parenting on the relationship between parent and sibling bereavement outcomes after the death of a child | *Quantitative* | 12.5 |  | “Morris aims to explore the relationship between parents’ and surviving sibling’s mental health symptoms (i.e., post-traumatic stress disorder [PTSD], prolonged grief disorder (PGD), and depression symptoms) after a child’s death. Additionally, extent to which parent functioning indirectly impacted sibling functioning through parenting behaviors (i.e., positive parenting and parent involvement) was also examined, with a specific focus on differences based on parent gender.” | 60 bereaved parents and siblings (aged 8-18) / families our of 110 families of children who had died between years 2008-2013. Measures:   1. DSM-IV 2. PTSD-RI 3. Caregiver depression 4. Prolonged grief disorder (PG-13) | - Maternal, but not paternal, symptoms of PTSD and PGD were directly associated with sibling outcomes. - Paternal symptoms were associated with sibling symptoms indirectly, through parenting behaviors (i.e., via decreasing positive parenting) | Differential impact of maternal vs paternal symptoms on siblings, stressing the importance of addressing postloss symptoms from a family perspective |
| Niang-Huei Peng et al 2012 | Cultural Practices and End-of-Life Decision Making in the Neonatal Intensive Care Unit in Taiwan | *Quantitative* | 9 |  | “The purpose of this research was to describe conditions of decision making for dying infants and cultural effects on the process of infant death in the neonatal intensive care unit.” | “A retrospective chart review was used in this research. Fifty charts were reviewed; the major cause of death for the research subjects was complications of prematurity (52%).” | “Eighty-two percent of the charts documented a do not resuscitate order, and 16% of parents allowed discontinuation of ventilator support when they realized the futility of continued care. In 30 nursing records, parents and their dying infants were offered a quiet place in which to grieve. In 10 cases, nurses accompanied these parents and allowed them to express their emotions.” | Research findings showed great outward expression of religion at the time of death. Various cultural issues affected both the grieving process of the families and the dying process of their infants. |
| Nuss, S.L., 2014 | Redefining parenthood: surviving the death of a child | *Qualitative* |  | 21 | “Although dying children are often aware of their impending death, parents are reluctant to communicate with their dying child about death. Nuss seeks to examine how parents of children in the advanced stage of a life-threatening disease trajectory communicated about death.” | “Grounded theory methods  Data collected via interviews with 18 parents of children who had died of an advanced life-threatening disease. Of 34 families invited, 15 (44%) agreed to participate.” | “From onset of their child’s illness, sense of parental self was threatened and parents confronted a process of ‘Redefining Parenthood’. 10 subprocesses”:   1. Protecting from fears 2. Protecting Normalcy 3. Protecting faith 4. Experiencing protection from their child 5. Bookmarking memories   After child’s death:   1. Telling the story 2. Making meaning 3. Protecting child’s memory 4. Defining a new normal 5. Learning to live with regret | “The communication style (open, semiopen, or closed) used by parents depended on several factors, including their existing communication style, the parental perception of how much their child could comprehend, and the need to stay positive.”  When children are diagnosed with a life-threatening illness, healthcare professionals often encourage new patients and families to talk to experienced patients and families to hear a first-hand account of what to expect during treatment and beyond. 🡪 HCPs could arrange for these interactions before the child’s death to facilitate the supportive network. |
| Patricia O' Malley et al, 2014 | Death of Child in the Emergency Department | *Technical report* | NA | NA | “The death of a child in the emergency department (ED) is one of the most challenging problems facing ED clinicians. This revised technical report and accompanying policy statement reaffirm principles of patient- and family-centered care. Recent literature is examined regarding family presence, termination of  resuscitation, bereavement responsibilities of ED clinicians, support of child fatality review efforts, and other issues inherent in caring for the patient, family, and staff when a child dies in the ED.” | Technical report. | Results of parent surveys confirm that the delivery of the news of their child’s death is extremely important to the long-term well-being of family members.   - The importance of communication was emphasized. Skill and compassion in conveying bad news may be the most powerful therapeutic tool clinicians can offer affected families. | “Studies in children with known lifespan–limiting conditions report that between 3% and 20% of deaths in that population will occur in the ED. Explicit and anticipatory collaboration between pediatric palliative care services and their corresponding EDs will likely improve care for such children. Many children receiving palliative care have had the opportunity to develop advance care plans.  Family presence has received widespread endorsement. Initial resistance to allowing family presence during attempted resuscitation was based on fears of litigation and concerns that the emotional burden for family members of watching resuscitation would create situations that would distract ED personnel, potentially interfere with effective resuscitation efforts, and only add to a family’s burden of grief. These fears have been systematically studied and for the most part clarified or eliminated.”  The question of when to stop resuscitation efforts- at least 2 studies in adult patients indicate that families may in fact adjust better after pronouncement on scene than with transport to a hospital. However, hall et al noted that paramedics are far more uncomfortable with termination of efforts in the field for a child than for an adult. , a child or infant may be transported to the hospital even though the resuscitative efforts may be futile, in order to provide a setting with better resources for support of the family and providers.  The healthcare system should reduce the workload on the family as much as possible by helping them to do somethings like-  The development of a policy and procedure for handling of the body may include the following:   - death packet and checklist to ensure that all appropriate notifications are accomplished; - documentation of release of valuables; - documentation of release of the body; - notification of a funeral home; • completion of the death certificate in accordance with state law, as applicable; and - notification of the child’s primary care provider. (Families expect that their primary care provider will be aware of their child’s death, and the task of notifying the medical home and others of a child’s team should not fall to the family.) |
| Penson, R.T., et al. (2002) | When does the responsibility of our care end: bereavement | *Non empirical* | NA | NA | “The Schwartz Center Rounds, a monthly mutli-disciplinary forum where caregivers reflect on important psychosocial issues faced by patients, families and caregivers.” | “Two vignettes presented during the forum in the form of a caregiver’s response to the death of a patient.” | Optimal end of life care: anticipating bereavement   - ‘good’ death based on physical comfort, quality of personal relationships, finding meaning in their life and death, feeling some sense of control in the situation, and active preparation for death   Counselling   - Over the first month, expression of grief should be encouraged and the person reassured that it is a normal human reaction - Screen of psychosocial domains   Complicated grief   - When normal bereavement is associated with psychiatric sequelae and overlaps with an adjustment disorder   Condolence letter (support from the HCPs and medical staff) and provision of bereavement resources  Condolence letters gives a personal touch, communicating compassionate care. | “Cancer care professionals are confronted with the limits of modern medicine when faced with the death of their patient.  There is responsibility to provide grieving families with support and care.  Medical staff have a responsibility to provide grieving families with support and care.” |
| Pritchard, M., et al. (2009) | Bereaved parents’ perceptions about when their child’s cancer-related death would occur. | *Qualitative* |  | 22 | “Parents of terminally ill children with cancer frequently ask clinicians when their child will die. Such information helps parents prepare for the child’s death. Pritchard seeks to identify how parents perceived when their child’s cancer-related death would occur.” | “Parents of patients from the St. Jude Children’s Research Hospital in the United States were approached. Secondary analysis of telephone interviews with 49 bereaved parents (out of 65 parents participating in a larger study) 6-10 months after their child’s death. Qualitative method of semantic content analysis was used to review all transcribed interviews and identified segments in which parents described their perceptions about when their child’s death would occur.” | Parents knew in advance that their child was going to die, but described their child’s death in 3 different ways:   1. Anticipated (52.4%) 🡪 observed changes that alerted them that death was imminent 2. Surprising (31%) 🡪 surprised that child died on that particular day   Overdue (16.7%) 🡪 had been waiting for the end of their child’s apparent suffering | “Parents of children with terminal cancer can perceive when their child’s death would occur very differently: some are surprised, whereas others feel that they have waited too long for their child to be released from suffering.  Clinicians can use these descriptions and the associated symptom patterns to help families prepare for their child’s last week and day. Parents are likely to find comfort in knowing what the experience was like for other parents, or when child’s clinicians are willing to discuss how and when their child’s death might occur.” |
| Reder, E.A., and Serwint, J.R. (2009) | Until the last breath: exploring the concept of hope for parents and health care professionals during a child’s serious illness | *Qualitative* |  | 20 | “A common situation seen: family’s desire to maintain hope in the face of a poor prognosis. Bereaved parents express maintaining hope as an important factor in how they coped with their child’s illness. HCPs are concerned that a family’s expression of hope may be related to their inability to accept the reality of the situation, which could lead to continued aggressive treatment that may cause patient needless suffering. Reder and Serwint hence seek to investigate the concept of hope for families and pediatric HCPs during a child’s serious illness.” | “8 focus groups were held with bereaved parents, residents, physicians and nurses at the Johns Hopkins Children’s Center in Baltimore from 2005 to 2006. Total of 39 participants. Audio transcripts were recorded and later transcribed for qualitative analysis.” | 2 themes emerged:   1. Balancing hope with accepting the reality of prognosis  - Parents identified their role as bearers of hope; was a cornerstone of decision making - Parents felt that maintaining hope and accepting the outcome were not mutual exclusive. - HCPs tend to view hope as related to a positive outcome, and some had difficulty in maintaining hope in face of worsening prognostic data - Nurses were worried that parents were hopeful as they had not been given honest information  1. Balancing hope without prolonging the patient’s suffering   Parents noted the tension specifically in terms of selfishness | “Hope is a survival tool in coping with serious illness. Parents spoke about their role as bearers of hope and protectors of their children, which could indicate that they are operating from the concept of hope as an innate aspect of humanness and parenthood.”  “Crucial for HCPs to understand how each individual family approaches hope.”    “One strategy could be to communicate with parents about hoping for the best but preparing for the worst.  Communication is key.” |
| Rhona, R., et al., 2012 | Bereaved Parents' Perspectives on Pediatric Palliative Care | *Qualitative* |  | 21 | “This study’s goal was to describe and begin to understand the experience of bereaved parents whose deceased child had received pediatric oncology services at a tertiary comprehensive cancer center.” | “Focus groups were conducted with parents whose children were age 10 years and older at the time of death. Potential participants were contacted by mail and telephone. Sessions were audiotaped and transcribed verbatim. The ATLAS.ti qualitative software program was used to identify and analyze dominant themes.” | “Fourteen parents identified four major themes: standards of care, emotional care, communication, and social support. Bereaved parents discussed the challenges associated with institutional procedures and interpersonal aspects of care in anticipation of and following their child’s death.”  Themes:  Standard of care- Themes that emerged from further analysis of standards of care included: (a) knowledge of processes and negotiation within the institution; (b) development of trusted relationships with treatment providers; (c) personalized patient accommodation; and (d) accommodation for caregivers and visitors, including young children.  Emotional care- Parents described the child’s ambivalence to talk about death and the importance of the child having control regarding end-of-life discussions. Parents described their need and their family’s need for anticipatory grief counseling.  Communication- Participants had appreciated the provider’s time for discussion and understood that a provider’s availability fluxed in accordance with the child’s health status. Participants needed providers to lead end-of-life conversations.  Social support -Parents emphasized the importance of discussing social support needs withproviders and maximizing social connections in the treatment plan. | “Of the emergent domains, two had been identified in the literature review and through expert consultations: the emotional care of family members and communication between family members and providers. Two primary areas of interest to participants of this study were standards of care and social support.  Parents in this study stressed the importance of establishing long-term relationships and effective communication with their child’s health care providers. Many of the suggestions made by these parents applied to both primary oncology and palliative care and were not limited to one time segment in the child’s life. The level of importance ascribed to particular aspects of care varied according to time, experience, and health status. A trajectory of serious illness may be unpredictable and the needs of children and families are dynamic. Priorities of care should be reassessed as the child’s health condition changes.  The results of these personal narratives may be used to guide care plans and deliver pediatric palliative and end-of-life interventions.” |
| Rosenbaum, J. L. et al, 2011 | Neonatal End-of-Life Spiritual Support Care | *Descriptive* |  |  | “The death of an infant is a profound loss that may complicate, disrupt, or end relationships between parents; and lead to maladaptive grieving, long-term decreased quality of life, and symptoms related to psychological morbidity. Facing neonatal loss is frequently experienced as traumatic assault on parents’ spiritual and existential world of meaning. This article highlights the importance of supporting parents through loss by providing comprehensive care that focuses not only on the neonate’s physical needs, but also addresses parents’ and families’ spiritual, religious, and existential needs. Our objective is to increase practitioners’ awareness of spiritual and existential distress and to provide strategies to address such needs, particularly at the end of life.” | Case series | Themes identified through the case reports are:   - Search for meaning and existential answers - A higher power - Religion as a source of comfort and hope - Hope as a necessary ingredient for healing - Strategies and continuing education for providing spiritual care support and meaningful communication - Incorporating hope - Spiritual needs of parents at time of infant death   Continuing education: clinical pastoral education | “The majority of families rely on their faith and spirituality to assist them through their end-of-life journey. Health-care professionals need to become acquainted with the meanings of spiritual and religious concepts that emerge for patients and families in their clinical area or specialty. Comprehensive care of the whole person, particularly at the end of life or in critical care settings, not only in-cludes physical care but also includes sensitivity to spir-itual, religious, and existential concerns. As healthcare professionals become more aware of families’ religious and spiritual beliefs, they will likely feel more equipped to incorporate spiritual care at the end of life to sup-port families in the NICU. Although further research is needed in this area, incorporating spiritual support into end-of-life care in the NICU can facilitate a family’s grief journey.” |
| Rosenberg, A.R., et al. (2012) | Systematic review of psychological morbidities among bereaved parents of children with cancer | *Descriptive* |  |  | Bereaved parents have the most intense grief reactions, with increased risks of anxiety, depression, prolonged grief, and poor quality of life. The objective of this review was to comprehensively summarize existing studies utilizing validated instruments to measure psychological outcomes among bereaved parents of children with cancer. | Medline, Embase, CINAHL and PsychInfo were searched using the term “[(bereaved parent*) AND (neoplasm* OR cancer*)]. Studies were eligible if they included a population of bereaved parents of children with cancer, if the children were younger than 20 years-old at the time of death, and if the study utilized validated instruments to measure psychosocial outcomes.  Of the 121 initially identified studies, 102 failed to meet inclusion criteria and 19 were potentially eligible. Of these, 6 were ultimately excluded. 13 studies remained for qualitative synthesis. | - child’s duration of illness related to ultimate parental distress; parents of children whose cancer experience was shorter than 6 months or longer than 18 months had poorer adjustment. Previous loss was associated with worse outcomes and anticipatory grief was inversely related to abnormal grief responses. | There is less research on long term bereavement adaptation compared with that regarding the immediate end of life experience.  Parents of children with cancer have a unique experience which incorporates their child’s prolonged physical and emotional suffering and the possibility of preparative time before their child’s death. |
| Rosie Midson et al, 2010 | Addressing end of life care issues in a tertiary treatment centre: lessons learned from surveying parents' experiences | *Qualitative* |  | 14 | “Much of the work in children’s hospitals is rightly focused on treatments aimed towards cure but this means that death is often seen as a failure and, as such, it may not be discussed or acknowledged as a possibility until very late in a child’s stay in hospital. However, this reluctance can deny the child and their family the opportunity to be informed, prepare and make choices. A survey of the care received by parents whose child had died in a children’s tertiary treatment centre led to a greater understanding of the parents’ experiences and the ways in which care could be enhanced.” | “Parents were approached between 18 months and 1 year after child's death'; 'conducting the interview by telephone interview, home visit, or at the hospital in a room away from main building'. There were 2 surveys done - the 2006/7 survey and the 2008 survey. The data from both surveys were collated and analysed using descriptive statistics and thematic analysis.” | “2006/2007 survey: The qualitative data were grouped into three main themes which broadly reflected three aspects of the parents’ experiences: the impact of the environment; aspects of communication and information; and their feelings and emotions.”  Key points:   - Environment - This sense of fundamental geographical dislocation added to the parents’ distress. - Communication - both positive and negative reviews by the parents for both care during child stay and also care at the follow up visit after childs’ death. - Feelings and emotions - The parents’ emotions and feelings were diverse as expressed throughout their responses, and included feeling ‘lost’, ‘grateful’, ‘alone’, ‘shocked’, ‘frustrated’, ‘unprepared’, ‘exposed’, ‘on display’, ‘baffled’, ‘stunned’ and ‘stressed’.   Conducting the survey gave a sense of the experiences the families had undergone and identified areas for improvement through the development and implementation of the End of Life Care Pathway.  2008 survey:  The 2008 survey was triggered by concern that the Pathway Guidance was not being fully utilized.The themes of environment, communication, and emotions and feelings were identified in these data and the responses had similarities to those in 2006 – a mix of responses, with some reflecting that parents had been cared for by supportive, kind staff who had cared for them and their child as an individual, and some that reflected that more could or should have been done. | Three areas (education, awareness and empowerment) have been identified for specific attention with the aim of improving end of life care practice.   1. Continuing education of clinical staff, particularly in relation to communication, is a crucial element in bringing about change. Staff such as chaplains, psychologists, ethicists, social workers and members of the Palliative Care and End of Life Care teams whose work means that they have a familiarity with death and dying contribute to the ongoing education of clinical staff. Members of the PALS (patient advocacy and liaison service) team also provide valuable insights from their experiences of listening to families and are able to convey key messages. 2. Raising awareness of resources that are available to staff is important as this can help to support them in caring for children and families. This is particularly important when the goal of treatment changes from curative intervention to end of life support. The available resources can help support the staff after the death of a child. 3. Enabling families to be empowered by providing information on what they might expect, who is available to offer help and how they can become involved is important. The production of a family guide to the End of Life Care Pathway will provide one aspect of support. |
| Santos, M. R. D., et al., 2019 | From hospitalization to grief: meanings parents assign to their relationships with pediatric oncology professional | *Qualitative* |  | 19 | “To understand the meanings assigned by bereaved parents to their relationships with healthcare professionals during the end-of-life hospitalization of their child.”  “Considering that the parents’ impressions on the care they received may last for many years after the treatment ends(10), and that grief is not something that simply disappears over time – rather, it involves a psychic process – this study sought to understand the meanings assigned by bereaved parents to their relationships with healthcare professionals during their child’s hospitalization.” | “Qualitative-interpretative study based on hermeneutics. Data were collected from interviews with parents who were grieving the death of a child with cancer in the hospital and participant observation in an oncology ward. Deductive thematic analysis for data interpretation ensued.” | “The experience of parents is the sum of all relationships during treatment. Therefore, meanings form a tangle of interrelated senses built not only in the interaction with these professionals, but also with the child and with grief itself. In relationships with professionals, meanings related to the memories of the child, negative emotions and regret were identified.” | “Bereaved parents construct meanings throughout the entire disease trajectory in face of the multiple losses experienced.”  “The experiences and meanings of grief are shaped by the social processes and interactions experienced by the family in the hospital. These data show that the relationships established in the hospital at the end of a child’s life become part of the expressions of the family’s bereavement process.”  “The relationship with the professionals represents part of the support in coping with the grief after the child’s death in the hospital. It showed that the relationship with the professionals involves the continuity of the bond with the deceased child and the construction of a social space that keeps the child’s identity alive.” |
| Schaefer, M. R., et al., 2019 | Legacy Artwork in Pediatric Oncology: The Impact on Bereaved Caregivers' Psychological Functioning and Grief | *Mixed Methods* | 11 | 17 | “Legacy-making is rising in popularity in palliative medicine, although only one study has examined its impact in a pediatric population.”  “Legacy-making (e.g., memory books and recordings), is defined as a way for patients with terminal illness to create or do something for others as a means of remembrance.”  “***Objective:*** In response to the gaps in literature, this study (1) examines the impact of legacy artwork on bereaved caregivers' psychological functioning and grief and (2) compares caregivers' perceptions of support provided by the hospital throughout their child's cancer journey between the intervention and control groups.” | “Forty-four caregivers whose children died of cancer completed a demographic questionnaire specifically created for this study, the Brief Symptom Inventory-18, and the Prolonged Grief Disorder-13. They also answered questions regarding supportive services provided to them toward the end of the child's life, at the time of death, and after the child's death. Those caregivers who endorsed participating in legacy artwork were identified as the intervention group, whereas those who did not were classified as the control group.” | “There were no significant differences in psychological functioning among caregivers who participated in legacy artwork versus those who did not participate. However, caregivers who created legacy artwork with their child reported significantly less symptoms of prolonged grief and a greater perception of support from health care providers compared with caregivers who did not engage in this activity.” | “Although preliminary, these findings suggest that legacy artwork may have the potential to improve grief and overall satisfaction of support from the hospital in bereaved caregivers.”  “Owing to the nonsignificant differences in overall psychological functioning between the groups, it appears that legacy making may be better suited to address caregiver grief rather than general psychological distress.” |
| Sedig, L. K., et al., 2020 | Experiences at the End of Life From the Perspective of Bereaved Parents: Results of a Qualitative Focus Group Study | *Qualitative* |  | 18 | “There is little knowledge of the parental perceptions of care delivered and gaps experienced by families receiving end-of-life care.”  “This study reports the most helpful aspects of care provided during the end of life and identify opportunities to improve care delivery during this critical time.” | “This study consists of 2 one-hour focus group sessions with 6 participants each facilitated by a clinical psychologist to explore the experiences of bereaved parents of pediatric oncology patients at the end of their child’s life. The data were transcribed and coded using constant comparative analysis and evaluated for inter-rater reliability using intraclass correlation coefficient.” | “Four common themes were identified through qualitative analysis: (1) valued communication qualities, (2) valued provider qualities, (3) unmet needs, and (4) parental experiences. The most prevalent of these themes was unmet needs (mentioned 51 times). Subthemes were identified and evaluated.” | “Parents described struggling with communication from providers, loss of control in the hospital environment, and challenges associated with transition of care to hospice services.”  “The experiences bereaved parents of these pediatric oncology patients described were complex and unique to each individual.” |
| Sedig, L. K., et al., 2020 | Supporting pediatric patients and their families at the end of life: Perspectives from bereaved parents | *Qualitative* |  | 17 | “Cancer remains the leading cause of death by disease for children in the United States. It is imperative to optimize measures to support patients and families facing the end of a child’s life. This study asked bereaved parents to reflect on their child’s end-of-life care to identify which components of decision-making, supportive services, and communication were helpful, not helpful, or lacking.” | “An anonymous survey about end-of-life experiences was sent to families of children treated at a single institution who died of a malignancy between 2010 and 2017.” | “Most of the bereaved parents (61%) reported a desire for shared decision-making; this was described by 52% of families at the end of their child’s life. There was a statistically significant association between how well death went and whether the parental perception of actual decision-making aligned with desired decision-making (P ¼ .002). Families did not utilize many of the supportive services that are available including psychology and psychiatry (only 22% used). Respondents felt that additional services would have been helpful.” | “Health care providers should strive to participate in decision-making models that align with the preferences of the patient and family and provide excellent communication. Additional resources to support families following the death of a child should be identified for families or developed and funded if a gap in available services is identified.” |
| Seecharan, G.A., et al., 2004 | Parents’ assessment of quality of care and grief following a child’s death | *Qualitative* |  | 17 | “Deaths among children are rare, but the effect on family members is profound. Compared with adult deaths, information about grief, recovery, and quality of care is sparse. Seecharan aims to describe aspects of bereavement for parents who had experienced the death of a child and to compare these aspects by parent sex, type of death, and overall experience.” | “Of 193 families contacted, 59 (30.6%) were interviewed. In person interviews were conducted with these families, primarily parents (79 parents), a mean of 21.8 months after the child’s death.  3 components to the interview”:   1. Demographic questions about the child and the parent respondent 2. 2 existing questionnaires: Texas Revised Inventory of Grief, and the Comprehensive Assessment of Satisfaction with Care- Short Form   Series of questions about the quality of the child’s palliative care | Parent sex:   - Fathers and mothers had similar levels of grief   Type of death:   - Mothers who experienced the sudden death of their child had somewhat more intense grief reactions than those whose child died of a chronic condition   Overall experience   - Grief scores did not vary according to satisfaction with treatment | Other research has shown that mothers have a more intense grief reaction than fathers |
| Sieg, S. E., et al., 2019 | The Best Interests of Infants and Families During Palliative Care at the End of Life: A Review of the Literature | *Descriptive (Review)* |  |  | “Palliative care is an integral element of care provision in neonatal intensive care units (NICUs). Healthcare providers working in NICUs are likely to provide palliative care at some point in their career.”  **“Purpose:** This article examines what neonatal palliative care entails, how parents perceive healthcare providers' actions, what they potentially need at the end of their infant's life, and what bereavement interventions are most supportive for parents.” | “We conducted a search of full-text articles published in English in PubMed and CINAHL using the following key words: "NICU bereavement care," "end-of-life care," "infant loss," and "palliative care."” | “Healthcare providers should consider alleviation of the infant's pain and suffering when discussing whether to provide or continue aggressive medical interventions. The timing of these discussions is important.”  “Parents appear to be most comforted by compassionate, caring healthcare providers who show competence and knowledge in the provision of medical/nursing and palliative care.”  . | “Healthcare providers working in NICUs require specific training in bereavement/palliative care for infants.”  Families facing the death of their infant must receive support from qualified providers both during and after that death.  Furthermore, the infant’s quality of life must be considered when discussing withholding or withdrawing care.  “Memory boxes containing mementos of the infant have been found to be an effective intervention to facilitate the bereavement process by providing the parents with something they can take home. Such pictures should be more than just the traditional birth images—they should also include images of the infant with the parents and/or other family members.” |
| Sirki, K., et al., 2000 | Coping of parents and siblings with the death of a child with cancer: death after the terminal care compared with death during active anticancer therapy | *Qualitative* |  | 17 | “Traditionally, the primary emphasis has been on the parent-to-parent or parent-to-child relationship. How- ever. serious attention has also to be paid to the needs of the siblings.  Siriki aims to characterize the main problems and their occurrence during the mourning process in order to improve the terminal care programme and to learn how to help the families in this process.” | “Parents of 60/70 children after terminal care, and parents of 26/30 children who died during active anticancer therapy. were interviewed. Children were being treated at the Hospital for Children and Adolescents, University of Helsinki in Finland.  Structured interview to analyse occurrence of physicial and mental problems, return to work, changes in relationship between parents, self-reported recovery time and outlook of siblings.” | - Parents reported physical and/or mental problems with similar frequency (39% and 34%) - Average self-reported recovery times were similar (14 and 16mo) - pronounced differences were observed between the mothers and the fathers; the mothers requiring longer recovery times and returning to work later - Of the siblings, 18% in the terminal care group had problems compared with 32% in the active therapy group. These included fear, behavioral problems, problems with friends and school-related problems | Ability of the family to cope does not differ whether the child dies after terminal care or during active anticancer therapy. |
| Smørholm, Sesilie, 2016 | Suffering peacefully: experiences of infancy death in contemporary Zambia | *Qualitative* |  | 15 | “The article is a response to Wikan’s plea for “indepth studies that focus more on emotional experience in loss than on ritualized mourning” (1988:451) and has been inspired by her  descriptions of how silence may be part of a deliberate control of outward emotional expressions of grief and despair (1988, 1989, 1990, 1992).” | “This article is based on two years of anthropological fieldwork in Ng’ombe Township, in the capital Lusaka.” | “Based on long-term ethnographic fieldwork, this article explores how the bereaved mother’s silence is guided by wider cultural norms and values associated with death, by complex notions of what it means to be a person, and by local perceptions of mental health and well-being. To enhance the complexity of the mother’s silences, it also explores how structures  of poverty manifest in mothers’ experiences of loss and how silence may hold feelings of inadequacy but also of care and compassion. Finally, the article aims to provide a counterweight to the predominant assumption that mothers in poor communities, who experience high levels of infant mortality, fail to mourn the death of their babies, as well as to psychological theories that assumes verbal expressions as vital for the mourner’s mental recovery after loss.” | “The main focus of this article has been how mothers’ bereavement in Ng’ombe is guided  by norms of silence and how this silence is experienced and interpreted by the mothers themselves and those who care for them. Understanding the mother’s silence must, I suggest,  be grounded in comprehensive insights in local perceptions of health and well-being and also  of life and death, spirituality and personhood.” |
| Snaman, J., et al., 2020 | Reconsidering early parental grief following the death of a child from cancer: a new framework for future research and bereavement support | *Descriptive (Review)* |  |  | “Parents of children that die from cancer are at increased risk of significant long-term psychosocial and physical morbidities. Less, however, is known about the experience of parents early in the grief process.” | “Through review of the literature, previously conducted qualitative work, and extensive clinical experience working with bereaved parents, we developed a new framework for understanding, assessing, and studying parental grief during the first 2 years following the death of a child from cancer.” | “Our novel longitudinal framework hypothesizes that short- and long-term psychosocial sequalae in parents following the death of a child from cancer depend not only on pre-death factors but on the support present through the disease experience and the oscillation between protective factors and risk factors in the post-death period. We further hypothesize that protective factors and risk factors may be modifiable, making them key potential targets for supportive interventions aimed at augmenting protective factors and diminishing the effect of risk factors.” | “This is a new framework for understanding and assessing the grief experience of parents within the first 2 years of a child’s death.”  “This framework is adapted from a previously developed integrative risk factor framework for bereavement outcomes.”  Article talks about risk factors, protective factors, |
| Snaman, J.M., et al., 2016 | Helping parents live with the hole in their heart: the role of health care providers and institutions in the bereaved parents’ grief journeys | *Qualitative* |  | 17 | “Bereaved parents experience significant psychosocial and health sequelae, suggesting that this population may benefit from the ongoing extension of support and resources throughout the grief journey. The interaction of hospital staff with patients and families at the end of a child’s life and after death profoundly affects parental grief, offering a unique opportunity for the medical community to positively impact the bereavement experience. Snaman aims to explore the role of the health care team and medical institutions in the grief journey of parents whose child died a cancer-related death” | “Total of 13 parents whose child died during or after receiving medical care at St Jude Children’s Research Hospital were recruited to participate in the study. 11 (85%) of the recruited parents participated in 2 focus groups. 3 main prompts given”:   1. Tell me about your grief journey before your child’s death. What are some things that helped you as you went through this? 2. Tell me about your grief journey/ bereavement experience after your child’s death. What are some things that helped you? 3. What services can we offer to bereaved parents that would help them in their grief journey, and what is the appropriate time to offer them?   Responses were coded and analysed independently using semantic content analysis techniques. | 4 main concepts identified:   1. Importance of strong and ongoing relationships between providers and bereaved families 2. Importance of high quality communication 3. Effect of negative experiences between providers and bereaved families on parental grief   Importance of the institution’s role in the grief journeys of bereaved parents | “Bereaved parents consistently identified HCPs and the institution as integral aspects of their grief journeys. Need for formalized bereavement support to parents of children with life limiting conditions beginning during the child’s illness trajectory and extending well beyond the child’s death.” |
| Snaman, J.M., et al., 2017 | Empowering bereaved parents through the development of a comprehensive bereavement program | *Technical report* | NA | NA | “Parents who experience the loss of a child have a unique and valuable insights into the grief journey and can help health care providers identify key components intrinsic to the development, implementation and maintenance of a comprehensive bereavement program. Bereavement program at St. Jude Children’s Research Hospital was developed by pediatric palliative care experts in collaboration with bereaved parents to standardize and improve the institutional support provided to families around and after the death of a child.”  Article describes the component of a parent derived bereavement programme and presents early results on the effects of specific program components. | Review of the parent-inspired and parent-driven bereavement program at St Jude. 3 primary pillars of bereavement programme:   1. Clinical and supportive interventions 2. Parent-designed bereavement support materials   Research and educational opportunities involving bereaved parents | 1. Clinical and supportive interventions  - When a child has a poor prognosis or experiences a clinical decline near the end of life, anticipatory bereavement services are provided to the family by both the QOL team and bereavement coordinator to strengthen those supportive relationships that will continue beyond death. - long-standing parent mentor program, in which parents of a child with newly diagnosed cancer are paired with a trained parent mentor whose child is off active treatment.  1. Parent-designed bereavement support materials  - Initial condolence card   Bereaved parents also identified certain period or events as having a heightened risk of triggering significant grief reactions | “Parents and other family members who have experienced the death of a child require bereavement support. This support should be provided throughout the illness journey and extend into the bereavement period. Bereaved parents are an invaluable resource in the development of such a programme. The innovative nature of this parent-driven bereavement programme may serve as a paradigm for the development of other programmes.” |
| Sperandeo, D. D. S., 2020 | Post-decisional conflict in selecting cancer treatments: Perception of information disclosure may influence decisional conflict, decisional regret, and self-acceptance in bereaved parents of children with cancer | *Qualitative* |  | 18 | “This study aimed to establish a connection, if any, between perceptions of information disclosure about medical treatment and decisional conflict in bereaved parents of children with cancer. Decisional regret was an important theme in this exploration because decisional conflict strongly aligns with the propensity to mentally redo past events, thereby forming counterfactual alternatives to reality. People generate counterfactuals to hypothesize a more favorable outcome subsequent to a negative event or the death of a child as applicable to this study. A secondary objective was to investigate the potential influence of counterfactual processing and regret on the construct of self-acceptance: a phenomenon researchers have rarely studied in the population of interest.” | “Study participants included parents who lost a child to cancer in the United States after participating in medical treatment prescribed by a licensed oncologist. Cluster and convenience sampling were employed to recruit 92 participants. Quantitative methods were used in obtaining data samples through validated instruments for each independent and dependent variable.” | “The responses collected indicate that a perceived lack of information disclosure about treatment risks and efficacy, yield a positive influence on decisional conflict after the death of a child. Similarly, decisional conflict positively correlates with decisional regret, while the latter negatively correlates with self acceptance in the bereavement process.” | “The research implications call for additional studies that further isolate factors that contribute to decisional conflict. This study advocates for decision making tools and collaborative processes that ensure parents are well informed and involved in making medical decisions from diagnosis through palliative care, if a cure is not possible.” |
| Stevenson, M., et al., 2017 | Understanding how bereaved parents cope with their grief to inform the services provided to them | *Qualitative* |  | 18 | “Stevenson aims to develop a rich description of how parents experience their grief in the first year after the death of their child, and how various bereavement follow up and support services helped them during this time, with the aim of informing follow up and support services offered to bereaved parents.” | “Interpretive description methodology.  16 interviews with parents and seven interviews with HCPs were conducted.  Parents recruited from two tertiary cate hospitals in Canada. 21 bereaved parents were recruited as to how they experienced their grief in the first year, what was helpful or not helpful during this time, and their perspectives on various bereavement follow up and support services.  Also interviewed hospital HCPs who conduct bereavement follow- up activities, with questions pertaining to how they go about following-up with parents and what issues (if any) hinder their ability to do that follow-up” | 4 broad categories:   1. How parents coped with emotional reactions to their loss and demands of daily living at individual level 2. Social context of parents’ grief affected individual coping processes 3. Using various services to cope (intrapersonal and institutional)   Time and timing effect of passing of time on grief, and how this affects when services should be offered to bereaved parents | Parents seem to go back and forth between actively processing their grief and focusing on other aspects of their lives (Dual Process Model of Coping in Bereavement) 🡪 oscillation between loss-oriented and restoration-oriented coping  Meaning making is also crucial.  Continuing bonds to deceased child is an important element of coping with grief. |
| Sturrock, Colleen &  Louw, Johann, 2013 | Meaning-Making After Neonatal Death: Narratives of Xhosa-Speaking Women in South Africa | *Qualitative* |  | 16 | “The death of a neonate can be traumatic for mothers, resulting in profound grief, which ruptures their sense of coherence and identity.” | “A narrative approach was used to explore how six Xhosa-speaking women tell stories about the death of their baby to help them understand the significance of the loss.” | The two main themes were restoring shattered meanings and factors influencing the loss.  Subthemes under restoring shattered meetings are:   - Acknowledging the baby as a person to be mourned - Revisiting pregnancy, motherhood, and identity - Finding reasons for the loss | They struggled to establish a sense of their baby as a person to be mourned, to redefine their own identity, and to find reasons for the death. Their meaning-making was influenced by the baby’s father, older women in their community, and the context of deprivation in which they live. |
| Tan, J.S., et al., 2012 | Addressing parental bereavement support needs at the end of life for infants with complex chronic conditions | *Qualitative* |  | 17 | “Health care providers’ understanding of parental bereavement needs before and in the acute period following the death of an infant with a complex chronic condition are based upon models that outline the process of grief and provide direction for possible points of intervention.  Tan seeks to prospectively describe the bereavement experience of parents whose infants die in acute care settings with a complex chronic condition.” | “Longitudinal, qualitative, descriptive design used to explore the process of parental bereavement. Extreme case sampling with variation on race, socioeconomic status, prenatal diagnosis, and multiple gestations was use to select 7 cases presented by over 72 narrative interviews with parents.  Narrative-style interviews were open-ended and allowed parents to tell their story including how the infant’s illness and treatment were progressing. Probe questions were then used to explore topics pertaining to experiences during their child’s illness trajectory, such as the decision making process, coping mechanisms, and bereavement.  Analysis was conducted using a content analysis technique.” | Organized into 5 categories   1. Having expectations  - Entertaining possible outcomes: parents who anticipated undesirable situations appeared to be coping better following infant death - Decisions they can live with: less distress during bereavement when they had been actively involved in care decisions and had considered all potential interventions that were offered - Speaking with other families: communication with parents of other infants helped them anticipate likely courses of illness and form realistic expectations  1. Continuity of care  - Provider care: change in providers caused sense of abandonment. Some patents also started to doubt the therapeutic motives. .  1. Memory making  - Remembering time spent together - Physical memory making  1. Wide network of support  - Support from strangers - Affirmation of their strength and decisions made  1. Altruism  - Helping others through their experience   Passing on personal items | “Importance of evaluating treatment options in the context of the goals of care, rather than availability before offering them to parents.”    Idea of continuity of care is crucial 🡪 identification of a central provider. Parents also need someone to talk to who does not come to the conversation with a caregiver role agenda.  Early intervention in preparation for the possible death of an infant and establishment of resources to prospectively support parent bereavement including memory making. |
| **Thornton, R., et al., 2019** | Scoping Review of Memory Making in Bereavement Care for Parents After the Death of a Newborn | *Descriptive (Review)* | NA | NA | “Objective: To summarize and synthesize extant literature on memory making in bereavement care for parents who experience the death of a newborn and to identify opportunities for future research.” | “We conducted a systematic search of four health-related databases (MEDLINE Complete, CINAHL Complete, Embase, and PsychINFO) for original research in January 2019. We then conducted a manual search of the reference lists of all included articles and a citation search via Scopus.” | “Available research was focused primarily on parents’ perceptions of care during and after the death of their newborns.”  “Memory making interventions emerged as significant elements of the experiences of bereaved parent. Several researchers examined parents’ perceptions of specific memory making interventions, such as bereavement photography.”  “Contact with the newborn, opportunities for caregiving, bereavement photography, and the collection or creation of mementos emerged as important elements of memory making.” | “We identified few studies focused entirely on memory making as an intervention in the context of bereavement care for parents. However, memory making emerged as a recurring theme throughout qualitative and mixed method studies on parents’ perceptions of perinatal or neonatal end-of-life care.” |
| Ulrika Kreicbergs, et al., 2005 | Care-related distress: A Nationwide Study of Parents Who Lost Their Child to Cancer | *Quantitative* | 8.5 |  | “Palliative care is an important part of cancer treatment. However, little is known about how care-related factors affect bereaved intimates in a long-term perspective.  The authors conducted a population-based, nationwide study addressing this issue, focusing on potential care-related stressors in parents losing a child to cancer.” | “In 2001, the author attempted to contact all parents in Sweden who had lost a child to cancer in 1992 to 1997. The parents were asked, through an anonymous postal questionnaire, about their experience of the care given and to what extent these experiences still affect them today.  The questionnaire, which included 129 questions with a total of 365 items concerning the child's medical and nursing care and the parents' mental health 4 to 9 years after the loss.” | “Information was supplied by 449 (80%) of 561 eligible parents. Among 196 parents of children whose pain could not be relieved, 111 (57%) were still affected by it 4 to 9 years after bereavement. Among 138 parents reporting that the child had a difficult moment of death, 78 (57%) were still affected by it at follow-up. The probability of parents reporting that their child had a difficult moment of death was increased (relative risk 1.4; 95% CI, 1.0 to 1.8) if staff were not present at the moment of death. Ten percent of the parents (25 of 251 parents) were not satisfied with the care given during the last month at a pediatric hematology/oncology center; the corresponding figure for care at other hospitals was 20% (33 of 168 parents; P .0163).” | “Physical pain and the moment of death are two important issues to address in end-of-life care of children with cancer in trying to reduce long-term distress in bereaved parents.  Good communication and honest information are probably of utmost importance, as when communicating about death with children aware of their imminent death. Our study suggests that improved pain control, improved care during the moment of death, and improved follow-up after the child’s death are factors that may be rewarding to focus on in clinical practice to reduce long-term distress in bereaved parents. In other words, further improvement in the care of the sick child may also be beneficial for the parents in the long term.  Although palliative care is an important part of many diseases, little is known on how the care itself affects bereaved intimates in a long-term perspective.” |
| Valerie Jennings, et al, 2014 | Bereavement support used by mothers in Ireland and following the death of their child from a life-limiting condition | *Qualitative* |  | 19 | “Children’s palliative care is a rapidly developing specialism internationally. Bereavement support is an integral component of children’s palliative care but to date little research has investigated the bereavement support that mothers in Ireland use following the death of their child.”  The author aim’s to explore mothers’ experiences of bereavement support in Ireland following the death of their child from a life-limiting condition. | “A descriptive qualitative design was used. The study sample was ten mothers who had been bereaved in the previous 5 years.  All mothers were recruited to the study by a gatekeeper from a voluntary organisation. Data were obtained through unstructured single interviews and analysed using conventional content analysis.” | “The findings indicate that the mothers relied on a combination of informal and formal bereavement support. In addition to depending on others to provide support, the mothers described their ability to self-support. These three types of support were closely related and sometimes overlapped. Three themes were identified in the data: keeping the memory of the deceased child alive, seeking help, and positive thinking.  The findings also show that mothers in Ireland use a variety of sources of support following the death of their child from a life-limiting condition. Health professionals involved in caring for families and children with a life-limiting condition should have an understanding of these sources.” | “Health professionals could therefore help mothers to self-support by reinforcing their memories of their child and providing them with the opportunity to remember the child.  Health professionals must respect each mother’s individual coping style and be non-judgemental regarding a mother’s desire to maintain their deceased child’s memory.  Further research into this area is required to clarify aspects of continuing bonds, in particular why bereaved parents choose one method over another and the effect of different methods on each mothers’ grief. Greeff et al (2011) added that adaptation after loss requires support and resources on three levels: personal, familial, and societal.” |
| van der Geest, I. M., et al.  (2015) | Parents’ faith and hope during the Paediatric Palliative Phase and the Association with Long-Term Parental Adjustment | *Quantitative* | 8 |  | “The loss of a child is associated with an increased risk for developing psychological problems. However, studies investigating the impact of parents’ faith and hope for a cure during the palliative phase on long-term parental psychological functioning are limited. Van der Geest seeks to explore the role of faith and hope as a source of coping and indicator of long-term parental adjustment.” | “Participants in this study were parents who lost a child to cancer between January 2000 and December 2004, during or after treatment at the Erasmus Medical Centre – Sophia Children’s Hospital, Department of Pediatric Oncology/ Hematology. 89 parents (out of the 246 fathers and mothers) completed the questionnaires”:   1. Inventory of Traumatic Grief 2. Depression subscale of the Brief Symptom Inventory   “Questionnaire investigating parents’ experiences of palliative care, especially wrt perception of faith and hope during palliative phase. Explored extent to which parents’ faith has been strengthened because of their child’s disease and the extent to which parents have faith in a God or higher power.” | 1. Faith and hope  - 39% parents reported a religious affiliation - 21% indicated that faith was very important for them in the palliative phase, while 51% disagreed - 15% revealed their faith had become stronger, while 58% disagreed - majority of parents remained hopeful for a meaningful time with their child (n = 68, 76%); a pain-free death (n = 58, 65%); and a cure (n=30, 34%).  1. Parents’ sources of coping  - Most parents stated that the child helped them cope during the palliative phase (n = 70, 79%), and more than half of the parents agreed that health care professionals (n=46, 52%) and family and friends (n=45, 51%) helped them cope.  1. Long-term parental adjustment  - Twelve parents (14%) suffered from traumatic grief, and 22 parents (25%) showed symptoms of depression.  1. Impact of faith and hope for a cure   “Parents’ faith was not associated with less long-term traumatic grief (OR=0.86, p=0.51) or symptoms of depression (OR=0.95, p = 0.74), and parents’ hope for a cure was not related to more long-term traumatic grief (OR = 1.07, p = 0.71) or symptoms of depression (OR = 1.12, p = 0.47).” | “One reason why parents’ faith was not associated with traumatic grief or symptoms of depression in the long-term could be that in a secular society, faith is less often used by parents as a coping factor.”  Hope for a cure may not necessarily mean that parents are not able to accept their child’s situation; Current evidence suggests that in clinical practice, it is necessary to be honest about the prognosis but there is no need to discourage parents from hoping for a cure.  “The approach of hope may differ between individuals, for parents, hope is considered as a protector of their child, while for HCPs, hope is associated with positive health-related outcomes.” |
| Wendy G. Lichtenthal, et al. , 2015 | Bereavement Follow-Up After the Death of a Child as a Standard of Care in Pediatric Oncology | *Descriptive (Review)* | NA | NA | “After a child’s death to cancer, families commonly want continued connection with the healthcare team that cared for their child, yet bereavement follow-up is often sporadic.” | Review paper. | “A comprehensive literature search found that many bereaved parents experience poor psychological outcomes during bereavement and that parents want follow-up and benefit from continued connection with their child’s healthcare providers.  A member of the healthcare team should contact the family after a child’s death to assess family needs, to identify those at risk for negative psychosocial sequelae, to continue care, and to provide resources for bereavement support.” | “Evidence suggests that the standard of care  should consist of at least one meaningful contact between the healthcare team and bereaved parents to identify those at risk for negative psychosocial sequelae and to provide resources for bereavement support.” |
| Wiener, L., et al. (2020). | Helping parents prepare for their child's end of life: A retrospective survey of cancer-bereaved parents | *Mixed methods* | 10 | 18 | “Most parents vividly recall the weeks, days, and moments preceding their child’s death for years to come. Dissatisfaction with communication about their child’s condition and lack of guidance can contribute to stress prior to a child’s death. Based on findings from a study assessing the degree of preparation bereaved parents received and our collective clinical experience, the authors provide suggestions on end-of-life communication and guidance for parents.” | “Caregivers of a child who died from cancer were invited to complete a 46-item survey through a closed social media (Facebook) group (“Parents who lost children to cancer”). In four months’ time, 131 bereaved caregivers completed the survey. Results were analyzed using descriptive statistics, chi-square analyses, and a thematic content analysis framework. The mean age of the child at the time of death was 12.” | “Approximately 40% of the parents in this study felt unprepared for both the medical problems their child faced and how to respond to their child's emotional needs; fewer than 10% felt very prepared for either. Parents were more likely to feel unprepared when perceived suffering was high, highlighting the critical importance of communication and support from the healthcare team as an adjunct to optimal symptom control.” | “This study identified specific medical and emotional issues about which parents wanted greater preparation. First, several parents reported inadequate communication about prognosis. Second, parents wanted to understand expected physical changes at the EoL. Third, parents expressed a need for guidance on talking with the child about death.”  “Although clinicians sometimes wait for parents to ask about these topics or look for signals that such a conversation would be welcomed, our findings suggest that these three topics should be offered to all parents.” |
